# Expanding the substrate scope of pyrrolysyl-transfer RNA synthetase enzymes to include non-α-amino acids in vitro and in vivo

**DOI:** 10.1038/s41557-023-01224-y

**Published:** 2023-06-01

**Authors:** Riley Fricke, Cameron V. Swenson, Leah Tang Roe, Noah Xue Hamlish, Bhavana Shah, Zhongqi Zhang, Elise Ficaretta, Omer Ad, Sarah Smaga, Christine L. Gee, Abhishek Chatterjee, Alanna Schepartz

**Affiliations:** 1 Department of Chemistry, University of California, Berkeley, CA USA; 2 Center for Genetically Encoded Materials, University of California, Berkeley, CA USA; 3 Department of Molecular and Cell Biology, University of California, Berkeley, CA USA; 4 Process Development, Amgen, Thousand Oaks, CA USA; 5 Department of Chemistry, Boston College, Chestnut Hill, MA USA; 6 Department of Chemistry, Yale University, New Haven, CT USA; 7 Howard Hughes Medical Institute, University of California, Berkeley, CA USA; 8 California Institute for Quantitative Biosciences (QB3), University of California, Berkeley, CA USA; 9 Molecular Biophysics and Integrated Bioimaging Division, Lawrence Berkeley National Laboratory, Berkeley, CA USA; 10 Chan Zuckerberg Biohub-San Francisco, San Francisco, CA USA

**Keywords:** Enzymes, X-ray crystallography, Synthetic biology

## Abstract

The absence of orthogonal aminoacyl-transfer RNA (tRNA) synthetases that accept non-l-α-amino acids is a primary bottleneck hindering the in vivo translation of sequence-defined hetero-oligomers and biomaterials. Here we report that pyrrolysyl-tRNA synthetase (PylRS) and certain PylRS variants accept α-hydroxy, α-thio and *N*-formyl-l-α-amino acids, as well as α-carboxy acid monomers that are precursors to polyketide natural products. These monomers are accommodated and accepted by the translation apparatus in vitro; those with reactive nucleophiles are incorporated into proteins in vivo. High-resolution structural analysis of the complex formed between one PylRS enzyme and a *m-*substituted 2-benzylmalonic acid derivative revealed an active site that discriminates prochiral carboxylates and accommodates the large size and distinct electrostatics of an α-carboxy substituent. This work emphasizes the potential of PylRS-derived enzymes for acylating tRNA with monomers whose α-substituent diverges substantially from the α-amine of proteinogenic amino acids. These enzymes or derivatives thereof could synergize with natural or evolved ribosomes and/or translation factors to generate diverse sequence-defined non-protein heteropolymers.

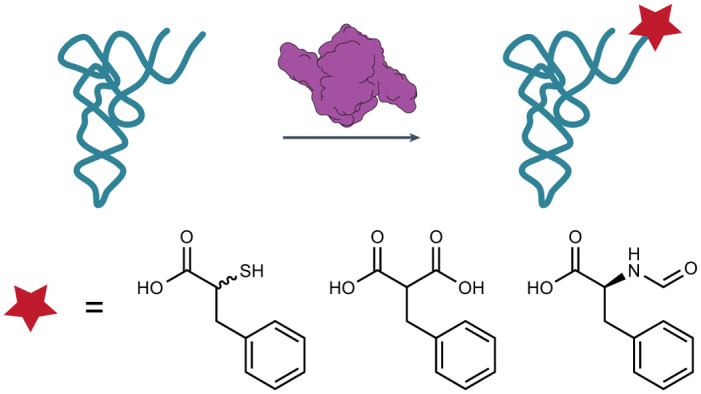

## Abstract (web summary)

Ribosomal incorporation of non-α-amino acid monomers into proteins is largely restricted to in vitro translation. Now, pyrrolysyl-transfer RNA synthetase variants have been shown to acylate tRNAs with α-thio acids, malonic acids, and *N*-formyl amino acids. This work represents a key step towards the programmed ribosomal synthesis of sequence-defined non-protein polymers in cellulo.

## Main

Extant organisms biosynthesize polypeptides in a messenger RNA template-dependent manner using a translation apparatus composed of ribosomes, aminoacyl-transfer RNA (tRNA) synthetases, tRNAs and multiple ancillary factors. Repurposing this translation apparatus for the templated in vivo synthesis of mixed-sequence hetero-oligomers—especially those containing non-l-α-amino acids—would provide a biological route to sequence-defined non-peptide polymers with original, tunable and evolvable properties and protein therapeutics with improved stability and expanded function. Chemical methods support the synthesis of sequence-defined non-peptide polymers^1,2^, but are limited to a small scale and produce considerable waste. Polymerization methods support the synthesis of sequence-controlled materials, but without rigorous sequence definition. By contrast, cellular methods generate little waste, especially on a large scale, achieve longer chain lengths and are scalable for industrial production.

It has been established over the past decade that many non-l-α-amino acids, including α-hydroxy acids^3,4^, d-α-^5^, linear^6–8^ and cyclic^9^ β-, γ-^10,11^, and long-chain amino acids^12^, α-aminoxy and α-hydrazino acids^13^, α-thio acids^14^, pyridazinone^15^, aminobenzoic acids^16,17^, and even 1,3-dicarbonyl monomers that resemble polyketide precursors^17^, are accepted by natural *Escherichia* *coli* ribosomes in small-scale in vitro reactions when acylated on tRNAs^18^. The structural and electronic diversity of these monomers emphasizes the important role of proximity in promoting bond-forming reactions within the *E. coli* peptidyl transferase centre (PTC)^19^. However, there are scant examples of non-l-α-amino acids having been incorporated into proteins in vivo^20–27^. The absence of orthogonal aminoacyl-tRNA synthetase (aaRS) enzymes that accept non-l-α-amino acid substrates is a primary bottleneck limiting the production of sequence-defined, non-peptide heteropolymers in vivo using wild-type (WT) or engineered ribosomes.

**Fig. 1:**
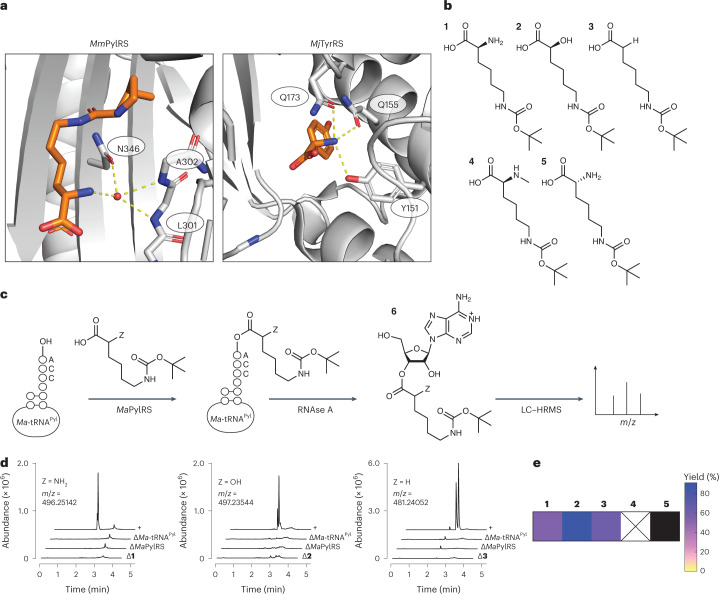
Promiscuous activity of WT *Ma*PylRS. **a**, The α-amines of l-α-amino acids are recognized differently by *Mm*PylRS (left)^37^ and *Mj*TyrRS (right)^36^. **b**, Analogues of l-BocK **1** evaluated as substrates for *Ma*PylRS. **c**, RNAse A assay used to detect the acylation of *Ma*-tRNA^Pyl^ with the BocK analogues shown in **b**. **d**, LC–HRMS analysis of *Ma*-tRNA^Pyl^ acylation reactions after RNAse A digestion. The peak masses correspond to adenosine nucleoside **6** acylated on the 2′- or 3′-hydroxy by the indicated monomer. The bottom three traces in each plot are negative controls that lack either the small molecule substrate (∆**1**, ∆**2**, or ∆**3**), enzyme (∆*Ma*PylRS), or tRNA (∆tRNA^Pyl^), while the top trace labeled ‘+’ contains all three necessary components for the acylation reaction. Two isobaric peaks are observed because the sample consists of adenosine acylated on either the 2′- or 3′-hydroxy group. **e**, Heat map illustrating the relative activities of substrates **1**–**5** for *Ma*PylRS, as determined by intact tRNA analysis (Supplementary Figs. 3–6) as described in Methods. The yields are reported as percentages based on intact tRNA analysis. The percentage yields were calculated as the ratio of tRNA acylated with the monomer of interest divided by the total tRNA detected by the LC–MS analysis. The black box indicates that no reaction product was detected; the box marked X indicates that the substrate was not investigated. Source data

In *E. coli*, two families of orthogonal aaRS enzymes have been widely employed to introduce hundreds of different non-canonical α-amino acid monomers^28–32^ into protein. The first includes pyrrolysyl-tRNA synthetase (PylRS) enzymes from methanogenic archaea and bacteria whose natural substrate is pyrrolysine^33^, an l-α-amino acid found in the active sites of certain enzymes involved in methane metabolism^34^. The second includes a large family of enzymes derived from *Methanocaldococcus jannaschii* tyrosyl-tRNA synthetase (*Mj*TyrRS)^28,35^. These two enzyme classes differ in how they recognize the α-amine of a bound substrate. While *Mj*TyrRS recognizes the substrate α-amine via multiple, direct hydrogen bonds to the side chains of Gln173, Gln155 and Tyr151 (Protein Data Bank (PDB): 1J1U)^36^, *Methanosarcina mazei* PylRS (*Mm*PylRS) instead uses water-mediated hydrogen bonds to the Asn346 side chain and Leu301 and Ala302 backbone amides (PDB: 2ZCE; Fig. 1a)^37^. These differences were exploited to acylate the cognate tRNA of *Mm*PylRS (*Mm*-tRNA^Pyl^) with a series of conservative *N*^ε^-*tert*-butoxycarbonyl-l-lysine (l-BocK) analogues containing -OH, -H and -NHCH_3_ in place of the α-amine (Fig. 1b)^22^.

We hypothesized that the α-amine recognition mode employed by *Mm*PylRS could provide sufficient space for substrates with less conservative substituents than the α-amine if the water molecule in the active site were displaced. Here we report that the tolerance of *Mm*PylRS for substrates with multiple alternative substituents in place of the α-amine extends to *Methanomethylophilus alvus* PylRS (*Ma*PylRS)^38,39^ as well as *Ma*PylRS variants (N166A V168L, *Ma*FRS1 and N166A V168K, *Ma*FRS2) that recognize diverse l-phenylalanine derivatives^40^. *Ma*FRS1 and *Ma*FRS2 also accept phenylalanine derivatives with α-thio, *N*-formyl-l-α-amino as well as an α-carboxy substituent: 2-benzylmalonic acid. A final variant, *Ma*FRSA (N166A V168A) (ref. ^41^), shows selectivity for ring-substituted 2-benzylmalonate derivatives over l-Phe.

Malonates contain a 1,3-dicarbonyl unit that represents the defining backbone of polyketide natural products, and after decarboxylation they have the potential to support Claisen-type condensation within the PTC to form a carbon–carbon bond. Structural analysis of *Ma*FRSA complexed with a *m*-substituted 2-benzylmalonate derivative and a non-hydrolysable ATP analogue revealed how the enzyme uses a unique pattern of hydrogen bonds to differentiate the two prochiral carboxylates in the substrate and accommodate the larger size and distinct electrostatics of an α-carboxy substituent. In vitro translation studies confirmed that tRNAs carrying α-thio, α-carboxy and *N*-formyl-l-α-amino acid monomers are effectively delivered to and accommodated by *E. coli* ribosomes. In vivo studies using traditional and engineered *E. coli* strains confirmed that *Ma*FRSA supports the biosynthesis of model proteins containing internal, aromatic, α-hydroxy acid monomers. This work describes the first aaRSs that accept α-thio acids and α-carboxy acids that could support carbon–carbon bond formation within the ribosome. These activities emphasize the potential of PylRS as a scaffold for evolving new enzymes that act in synergy with natural or evolved ribosomes to generate diverse sequence-defined non-protein heteropolymers.

## Results

### *Ma*PylRS retains much of the promiscuity of *Mm*PylRS

**Extended Data Fig. 1:**
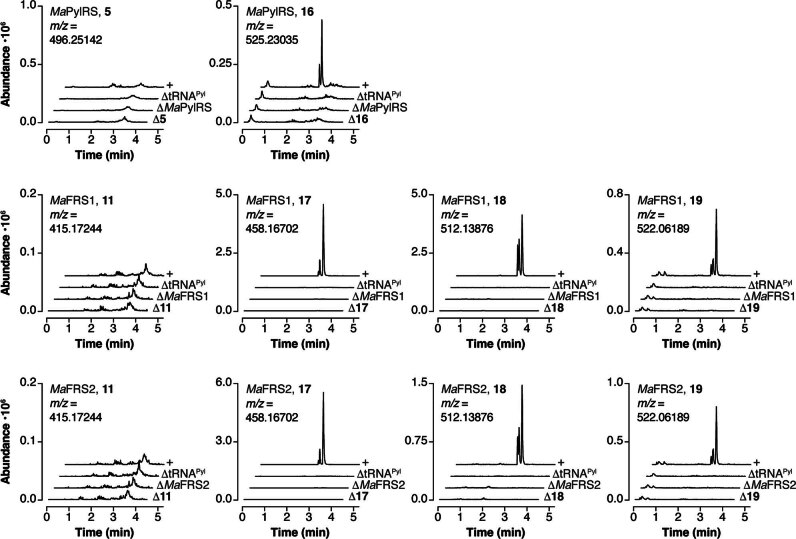
Additional RNAse A assay experiments analyzed by LC-HRMS. The enzyme and substrate are noted in the top left of each plot. These data provide evidence that *Ma*PylRS accepts **16** as a substrate but not **5**. Similarly, *Ma*FRS1 and *Ma*FRS2 accept **17**, **18**, and **19**, but not **11**. Source data

First, we set out to establish whether the expanded substrate scope of *Mm*PylRS reported for l-BocK analogues (Fig. 1b)^22^ was retained by *Ma*PylRS, which offers advantages over *Mm*PylRS because it lacks the poorly soluble amino-terminal tRNA-binding domain^42^ and is easier to express and evaluate in vitro^39^. The carboxy-terminal catalytic domain of *Mm*PylRS (ref. ^37^) is 36% identical to *Ma*PylRS and the structures are largely superimposable (Supplementary Fig. 1). To evaluate whether d-BocK as well as l-BocK analogues with -OH or -H in place of the α-amino group were substrates for *Ma*PylRS, we made use of a validated ribonuclease A (RNAse A) liquid chromatography (LC)–high-resolution mass spectrometry (HRMS) assay^17,43^. This assay exploits RNAse A to cleave the phosphodiester bonds following pyrimidine residues to generate 2′,3′-cyclic phosphate products^44^. As a result, the residue at the tRNA 3′ terminus is the only mononucleoside product lacking a phosphate (Fig. 1c). The incubation of l-BocK 1 (2 mM) with purified *Ma*PylRS (2.5 µM) and *Ma*-tRNA^Pyl^ (25 µM; Supplementary Fig. 2) at 37 °C for 2 h led to a pair of RNAse A digestion products whose expected mass (496.25142 Da) corresponds to the adenosine nucleoside **6** as a mixture of 2′- and 3′-acylated species (Fig. 1d) Although there is evidence that PylRS from *Methanosarcina barkeri* and *Desulfitobacterium hafniense* add Pyl to only the 3′-hydroxy group^45^, isomerization between the 2′ and 3′ isomers occurs rapidly^46^ and we were not able to identify which peak corresponds to which isomer. No products with this mass were observed when the reaction mixture lacked *Ma*-tRNA^Pyl^, l-BocK or *Ma*PylRS, and mass analysis of the intact tRNA product confirmed a 53.8% yield of acylated tRNA (**1-tRNA**^**Pyl**^; Supplementary Fig. 3). Under these conditions, l-BocK analogues with either -OH (**2**) or -H (**3**) in place of the α-amino group were also substrates for *Ma*PylRS, as judged by RNAse A (Fig. 1d) and intact tRNA mass spectrometry (MS) assays, with acylated tRNA yields of 90.5% (**2-tRNA**^**Pyl**^) and 61.6% (**3-tRNA**^**Pyl**^; Supplementary Figs. 4 and 5, respectively). No reactivity was detected with d-BocK (Supplementary Fig. 6 and Extended Data Fig. 1), perhaps because of differences in PylRS from *M. alvus* and *M. mazei*^47^. We conclude, therefore, that *Ma*PylRS retains much of the previously reported^22^ promiscuity of *Mm*PylRS. We note that certain non-natural monomers with relatively high activity, including **2** and **3**, led to measurable levels (2.5–13.7%) of diacylated tRNA (Supplementary Table 3). Diacylated tRNAs have been observed as products in cognate reactions of *Thermus thermophilus* phenylalanyl-tRNA synthetase (PheRS) (ref. ^48^), and they have been reported to support prokaryotic translation^49^.

### *Ma*PylRS variants retain activity for phenylalanine derivatives

**Fig. 2:**
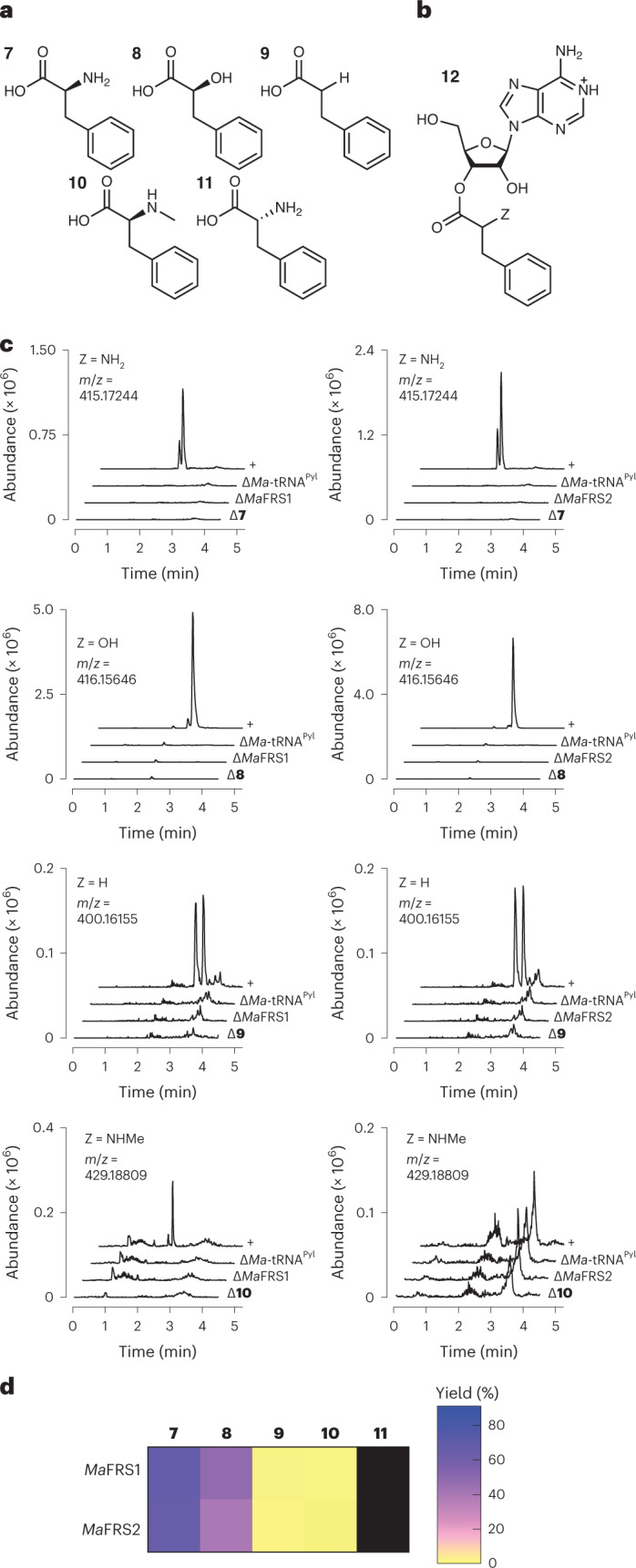
*Ma*FRS1 and *Ma*FRS2 process phenylalanine analogues with substitutions at the α-amine. **a**, Phenylalanine analogues evaluated as substrates for *Ma*FRS1 and *Ma*FRS2. **b**, Adenosine nucleoside **12** formed during RNAse A digestion of acyl-tRNA. **c**, LC–HRMS analysis of *Ma*-tRNA^Pyl^ acylation reactions after RNAse A digestion. Adenine nucleoside **12** acylated on the 2′- or 3′-hydroxy of the 3′-terminal ribose of *Ma*-tRNA^Pyl^ could be detected in *Ma*FRS1 and *Ma*FRS2 reactions with l-Phe (**7**) and substrate **8**; substrates **9** and **10** (Z = H, NHCH_3_) showed more modest reactivity. **d**, Heat map illustrating the relative yields with substrates **7**–**11** for *Ma*FRS1 and *Ma*FRS2, as determined by intact tRNA analysis (Supplementary Figs. 8–12 and 20–24) as described in Methods. The reported yields are percentages based on intact tRNA analysis. The black boxes indicate no reaction product was detected. Source data

PylRS is a subclass IIc aaRS that evolved from PheRS (ref. ^50^). *Mm*PylRS variants with substitutions at two positions that alter the architecture of the side-chain-binding pocket (Asn346 and Cys348; Supplementary Fig. 1) accept l-phenylalanine and its derivatives in place of pyrrolysine^29,40,50^. We integrated the mutations in the two variants that accept unsubstituted l-Phe (*Mm*FRS1 and *Mm*FRS2)^40^ into the *Ma*PylRS sequence to generate *Ma*FRS1 (N166A and V168L) and *Ma*FRS2 (N166A and V168K) (Supplementary Fig. 2). We then used RNAse A and intact tRNA MS assays to determine whether *Ma*FRS1 or *Ma*FRS2 retained the activity for l-phenylalanine (**7**) and its analogues in which the l-α-amino group was substituted by -OH (**8**), -H (**9**), -NHCH_3_ (**10**) or d-NH_2_ (**11**; Fig. 2a).

The incubation of l-Phe (**7**; 10 mM) with purified *Ma*FRS1 or *Ma*FRS2 (2.5 µM) and *Ma*-tRNA^Pyl^ (25 µM) at 37 °C for 2 h led in both cases to a pair of products whose expected mass (415.17244 Da) corresponds to adenosine nucleoside **12** (Z = l-NH_2_; Fig. 2b) as the expected mixture of 2′- and 3′-acylated products (Fig. 2c). No product with this mass was observed when the reaction mixture lacked *Ma*-tRNA^Pyl^, l-Phe, or *Ma*FRS1 or *Ma*FRS2, and mass analysis of the intact tRNA product confirmed yields of 66.1% (*Ma*FRS1) and 65.4% (*Ma*FRS2) of acylated tRNA (**7-tRNA**^**Pyl**^; Supplementary Figs. 8 and 20, respectively). l-Phe analogues **8**–**10** were also substrates for both *Ma*FRS1 and *Ma*FRS2, as judged by both RNAse A (Fig. 2c) and intact tRNA analysis (Supplementary Figs. 8–11 and 20–23), with reactivities decreasing in the order l-α-amino **7** > α-OH **8** ≫ α-H **9** ≈ *N*-Me-l-α-amino **10** based on intact tRNA yields (Fig. 2d). Mono- and diacylated tRNA products were observed for substrates **7** and **8** (Supplementary Table 3). Despite the fact that the desamino-BocK analogue **3** was a strong substrate for WT *Ma*PylRS, the desamino-Phe analogue **9** showed only modest activity with *Ma*FRS1 and *Ma*FRS2, as observed in the RNAse assay and intact tRNA analysis (Fig. 2c and Supplementary Figs. 10 and 22). Again, no reactivity was detected with d-Phe (Supplementary Figs. 12 and 24 and Extended Data Fig. 1).

### *Ma*FRS1 and *Ma*FRS2 process substrates with distinct α-substituents

**Fig. 3:**
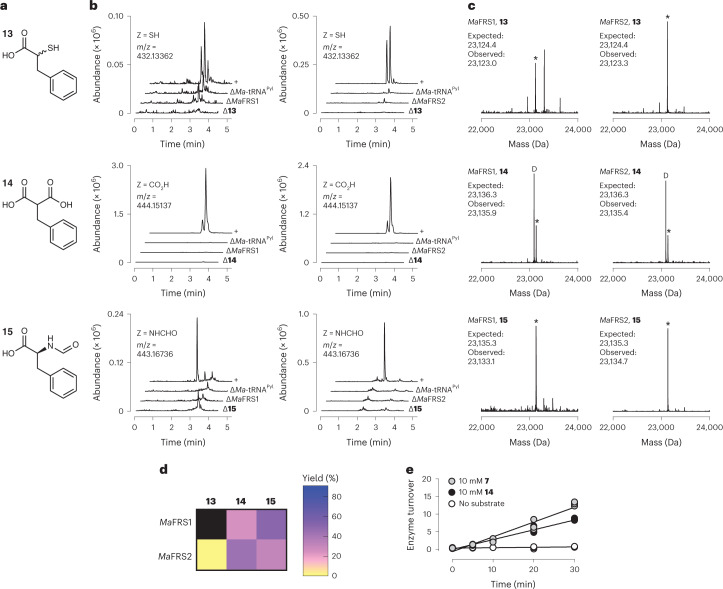
*Ma*FRS1 and *Ma*FRS2 process substrates bearing novel distinct α-substituents. **a**, Phenylalanine analogues **13**–**15** evaluated as substrates for *Ma*FRS1 and *Ma*FRS2. **b**, LC–HRMS analysis of *Ma*-tRNA^Pyl^ acylation reactions using *Ma*FRS1 or *Ma*FRS2 following RNAse A digestion. Adenosine nucleoside **12** acylated on the 2′- or 3′-hydroxy of the 3′-terminal ribose of *Ma*-tRNA^Pyl^ could be detected in *Ma*FRS1 and *Ma*FRS2 reactions with α-thio acid **13**, α-carboxy acid **14** and *N*-formyl-l-Phe **15**. **c**, LC–MS analysis of intact tRNA products confirms that monomers **13**–**15** are substrates for *Ma*FRS1 and *Ma*FRS2. An asterisk indicates the peak that corresponds to the indicated acyl-tRNA while a D indicates the peak that corresponds to the decarboxylation of the malonyl-tRNA. **d**, Heat map illustrating the relative activities of substrates **13**–**15** with *Ma*FRS1 and *Ma*FRS2, as determined by intact tRNA analysis (Supplementary Figs. 13–16 and 25–30) as described in Methods. The reported yields are percentages based on intact tRNA analysis. The black box indicates that no reaction product was detected. Initially no acyl-tRNA was detected when *Ma*FRS1 was incubated with **13**, but when the enzyme concentration was increased fivefold, acyl-tRNA was detected with mono- and diacyl yields of 0.7 and 9.7%, respectively. **e**, Turnover of *Ma*FRS1 over time with l-Phe (**7)** and 2-benzylmalonic acid (**14)** using the malachite green assay. A control with no substrate is shown for comparison. Data from three replicates are shown. Source data

We then began to explore phenylalanine analogues with larger and electrostatically distinct functional groups at the α-carbon: α-thio acids, *N*-formyl-l-α-amino acids and α-carboxy acids (malonates; Fig. 3a). α-Thio acids are substrates for extant *E. coli* ribosomes in analytical-scale in vitro translation reactions with yields as high as 87% of the corresponding α-amino acids^14^, and thioesters can persist in *E. coli* for more than 36 h (ref. ^51^). Peptides and proteins containing thioesters could also act as substrates for polyketide synthase (PKS) modules^52^, to generate unique keto-peptide natural products, or protein splicing reactions^53^. Formylation of methionyl-tRNA is important for initiating complex formation, and formylation could enhance initiation with non-methionyl α-amino acids in vivo^54,55^. Moreover, *E. coli* ribosomes incorporate monomers containing a 1,3-dicarbonyl moiety at the peptide N terminus to produce keto-peptide hybrids^17^. To our knowledge, there are currently no aaRS enzymes, orthogonal or otherwise, that accept α-thio, *N*-formyl-l-α-amino or α-carboxy acid substrates to generate the acylated tRNAs required for in vivo translation (when extant ribosomes are compatible) or ribosome evolution (when extant ribosomes are incompatible).

**Extended Data Fig. 2:**
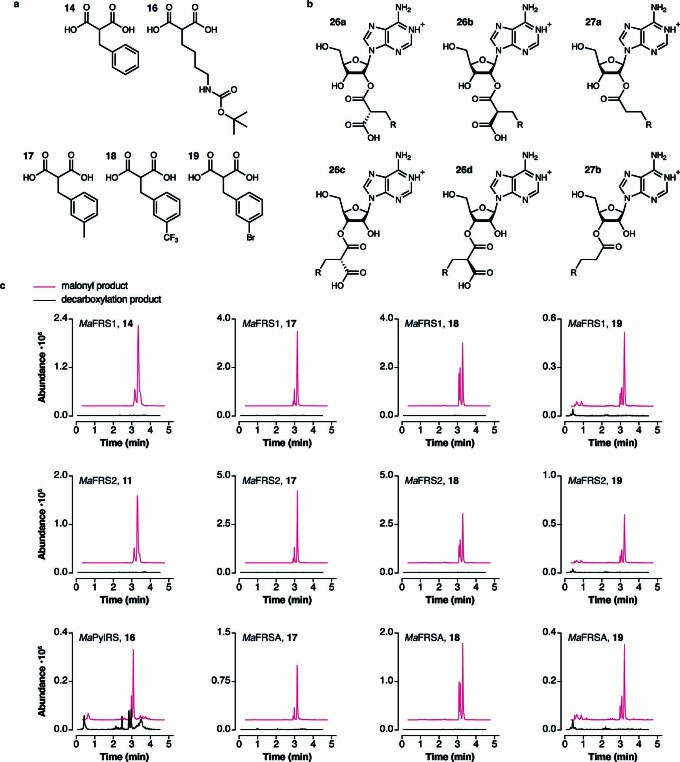
RNAse A assay analyzing decarboxylation of malonyl-tRNAs. **a**, Substituted malonic acid monomers used in this study. **b**, Left, **26a-d**: malonyl adenosine nucleosides formed when malonyl-tRNA is digested by RNAse A; right, **27a** and **27b**: decarboxylation products of malonyl-adenosine nucleosides. **c**, LC-HRMS analysis of *Ma*-tRNA^Pyl^ acylation reactions after RNAse A digestion. Reactions were performed as described in **Methods**. The EIC for the malonyl product **26** as a mixture of 2’ and 3’ isomers (two pairs of diastereomers) (pink, top) shows the expected peaks whereas the EIC for the decarboxylation product **27**, also as a mixture of 2’ and 3’ isomers (black, bottom) shows that the decarboxylation product is absent in all cases except the *Ma*PylRS-catalyzed acylation of *Ma*-tRNA^Pyl^ with monomer **16**. These data provide evidence that the decarboxylation of malonates charged to *Ma*-tRNAPyl observed in the intact tRNA mass analysis (Supplementary Figs. 7, 15, 17-19, 26, 28-30 and 32-35) occurs during either the workup or the LC-MS itself. Source data

We found that l-Phe analogues in which the α-amino was substituted with an α-thio (substrate **13)**, α-carboxy (substrate **14)** or *N*-formyl-l-α-amine (substrate **15)** moiety were all processed by *Ma*FRS1 and *Ma*FRS2 (Fig. 3a–c). In particular, α-carboxy acid **14** and *N*-formyl-l-α-amino acid **15** were excellent substrates. The incubation of α-carboxy acid **14** (10 mM) with *Ma*FRS1 or *Ma*FRS2 (2.5 µM) and *Ma*-tRNA^Pyl^ (25 µM) at 37 °C for 2 h, followed by digestion by RNAse A led to the formation of a pair of products whose expected mass (444.15137 Da) corresponds to the adenosine nucleoside **12** (Z = COOH). LC–MS analysis of the intact tRNA products confirmed yields of 24.4% (*Ma*FRS1) and 43.7% (*Ma*FRS2) of acylated tRNA (**14-tRNA**^**Pyl**^) (Supplementary Figs. 15 and 26, respectively). Because the α-carbon of substrate **14** is prochiral, mono-acylation of *Ma*-tRNA^Pyl^ can generate two diastereomeric product pairs—one pair in which the 3′-hydroxy group is acylated by the *pro-S* or *pro-R* carboxylate and another in which the 2′-hydroxy group is acylated by the *pro-S* or *pro-R* carboxylate. These diastereomeric products result from alternative substrate orientations within the enzyme active site (vide infra). We note that intact tRNAs acylated with 2-benzylmalonic acid (**14**) showed evidence of decarboxylation (indicated by a D in Fig. 3c). No evidence for decarboxylation was observed when the same acyl-tRNAs were evaluated using the RNAse A assay (Extended Data Fig. 2), suggesting that decarboxylation occurs either during work-up or during the LC–MS injection of intact tRNA.

The incubation of *N*-formyl-l-α-amino acid **15** under identical conditions led to the expected adenosine nucleoside **12** (Z = NHCHO), and LC–MS analysis confirmed yields of 50.0% (*Ma*FRS1) and 31.1% (*Ma*FRS2) of acylated tRNA (**15-tRNA**^**Pyl**^; Supplementary Figs. 16 and 27, respectively). The α-thio l-Phe analogue **13** was also a substrate for *Ma*FRS1 and *Ma*FRS2, although higher concentrations of *Ma*FRS1 were necessary to observe the acyl-tRNA product (**13-tRNA**^**Pyl**^) using intact tRNA LC–MS (Supplementary Figs. 13, 14 and 25). These results illustrate that the active-site pocket that engages the α-amine in PylRS can accommodate substituents with substantial differences in mass (-NHCHO) and charge (-COO^−^). Despite these differences, 2-benzylmalonic acid (2-BMA, **14**) is an excellent substrate for *Ma*FRS1 and *Ma*FRS2: kinetic analysis using the malachite green assay^56^ revealed a rate of adenylation by *Ma*FRS1 that was 66% of the rate observed for l-Phe (Fig. 3e). The tolerance for malonate substrates extends to WT *Ma*PylRS itself: the α-carboxylate analogue of l-BocK **16** was also a measurable substrate for WT *Ma*PylRS (Supplementary Fig. 7 and Extended Data Fig. 1).

### *Ma*FRSA processes *m*-2-benzylmalonic acids but not l-Phe

**Fig. 4:**
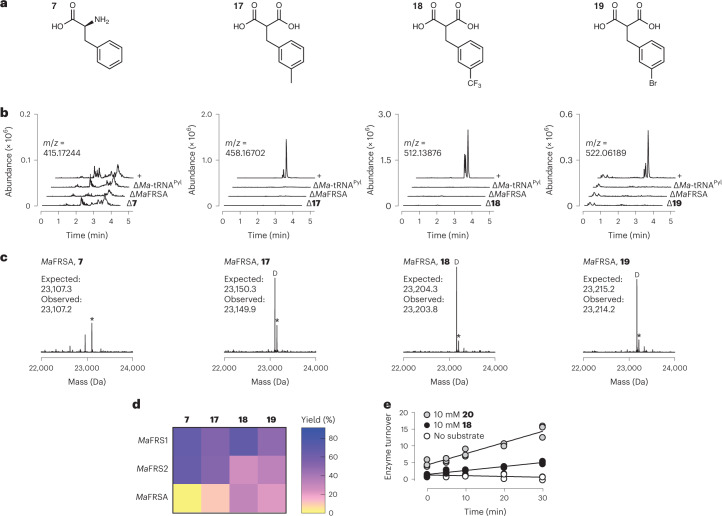
*Ma*FRSA selectively acylates *Ma*-tRNA^Pyl^ with *m*-substituted 2-BMA derivatives. **a**, Phenylalanine analogues **17**–**19** evaluated as substrates for *Ma*FRSA. **b**, LC**–**HRMS analysis of *Ma*-tRNA^Pyl^ acylation products after digestion with RNAse A. **c**, LC–MS analysis of intact tRNA products confirms that *m*-substituted 2-BMAs **17**–**19** are substrates for *Ma*FRSA. An asterisk indicates the peak that corresponds to the indicated acyl-tRNA while a D indicates the decarboxylation product of the malonyl-tRNA. **d**, Heat map illustrating the relative activities of l-Phe (**7**) and substrates **17**–**19** with *Ma*FRS1, *Ma*FRS2 and *Ma*FRSA. The reported yields are percentages based on intact tRNA analysis. **e**, Turnover of *Ma*FRSA over time with *m*-CF_3_-l-Phe (**20**) and *m*-CF_3_-2-BMA (**18)** using the malachite green assay. A control with no substrate is shown for comparison. Data from three replicates are shown. Source data

We have demonstrated that *Ma*FRS1 and *Ma*FRS2 have the ability to process substrates with unusual α-substituents, but they can also process l-Phe with comparable efficiency (Fig. 2d), which would interfere with selective charging of the non-l-α-amino acid. Variants of *Mm*PylRS that accept *p*-, *o-* and *m*-substituted l-Phe derivatives have been reported^40,41,57–59^. In particular, *Mm*PylRS containing two active-site mutations (N346A and C348A; henceforth referred to as FRSA) shows high activity for l-Phe analogues with bulky alkyl substituents and low activity towards l-Phe (ref. ^41^). We expressed and purified an *Ma*PylRS variant containing these mutations (*Ma*FRSA with N166A and V168A; Supplementary Fig. 2) and demonstrated using RNAse A (Fig. 4b) and intact tRNA analysis (Fig. 4c and Supplementary Figs. 31–34) that it shows high activity for derivatives of malonic acid **14** carrying *m*-CH_3_ (**17**), *m*-CF_3_ (**18**) and *m*-Br (**19**) substituents and low activity for l-Phe. Again, intact tRNA LC–MS showed evidence of decarboxylation, but this was not observed in the RNAse A assay (Extended Data Fig. 2). Of the *m*-substituted 2-BMAs, *Ma*FRSA showed the highest activity for *m*-CF_3_-2-benzylmalonate (*m*-CF_3_-2-BMA, **18**). Kinetic analysis^56^ revealed a rate of adenylation that was 36% of the rate observed for its l-α-amino acid counterpart *m*-trifluoromethyl-l-phenylalanine (*m*-CF_3_-Phe, **20**; Fig. 4e). Although derivatives of malonate **14** carrying *m*-CH_3_ (**17**), *m*-CF_3_ (**18**) and *m*-Br (**19**) substituents are also excellent substrates for *Ma*FRS1 (Supplementary Figs. 17–19) and *Ma*FRS2 (Supplementary Figs. 28–30), *Ma*FRSA shows substantially lower activity for l-Phe, allowing the selective acylation of tRNA with *m*-substituted 2-BMAs without interference from l-Phe (Fig. 4d). Indeed, when *Ma*FRSA was incubated with an equal concentration (10 mM) of l-Phe (**7**) and *m*-CF_3_-2-BMA (**18**), only the malonyl product (**18**-**tRNA**^**Pyl**^) was observed (Supplementary Fig. 35).

### Structure of the *Ma*FRSA/*m*-CF_3_-2-BMA complex

**Fig. 5:**
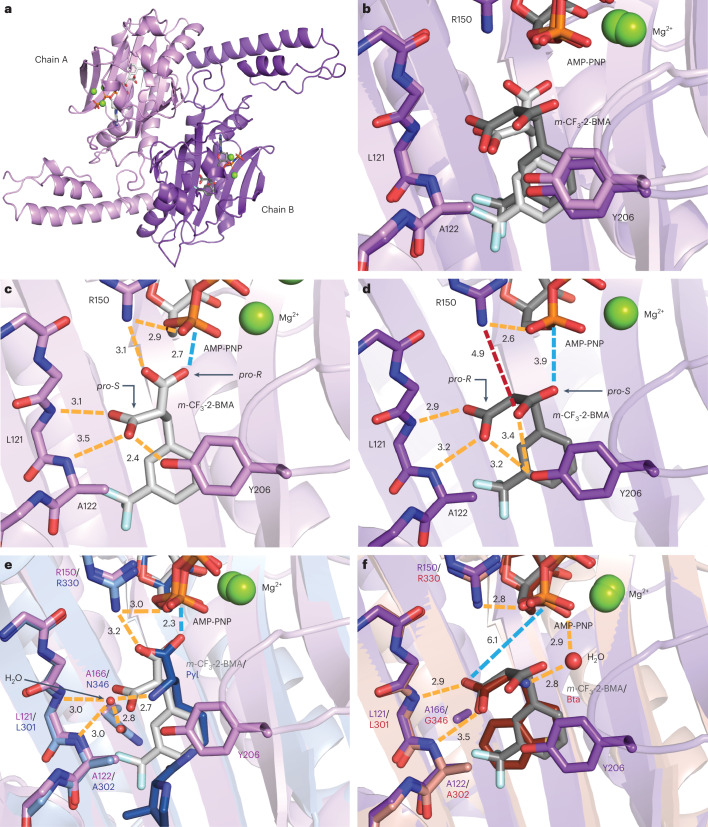
Structure of *Ma*FRSA bound to *m*-CF_3_-2-BMA and AMP-PNP reveals basis for distinct reactivity at *pro*-*R* and *pro*-*S* substrate carboxylates. **a**, Structure of the *Ma*FRSA dimer containing two non-identical chains in the asymmetric unit. **b**, Alignment of the active sites of chains A (light purple) and B (dark purple) reveals *m*-CF_3_-2-BMA (grey) bound in two alternative conformations. **c**, In chain A, *m*-CF_3_-2-BMA is coordinated by an extensive hydrogen bond network (orange dashes) that positions the *pro-R* carboxylate oxygen for nucleophilic attack (blue dashes). Interatomic distances (in Å) are shown alongside the dashed lines. **d**, In chain B, *m*-CF_3_-2-BMA is coordinated by similar hydrogen bonds, but in this case the *pro*-*S* carboxylate is rotated away from AMP-PNP with a loss of the hydrogen bond to Arg150 (red dashes) and a longer distance between the *pro*-*S* carboxylate nucleophile and the α-phosphate of AMP-PNP. **e**, Alignment of active site A with WT *Mm*PylRS bound to Pyl and AMP-PNP (PDB: 2ZCE, blue)^37^ illustrates the difference between the water-mediated hydrogen bonds (orange dashes) to the α-amine of Pyl in PylRS compared with the direct carboxy-to-backbone hydrogen bonding of *m*-CF_3_-2-BMA bound to *Ma*FRSA. **f**, Comparison of active site B with *Mm*BtaRS (N346G, C348Q) bound to Bta (PDB: 4ZIB, red)^62^ reveals similar binding modes with hydrogen bonds (orange dashes) between the substrate carboxylate and backbone amide of the enzyme when Asn166/Asn346 (*Ma*/*Mm* numbering) is mutated.

**Extended Data Fig. 3:**
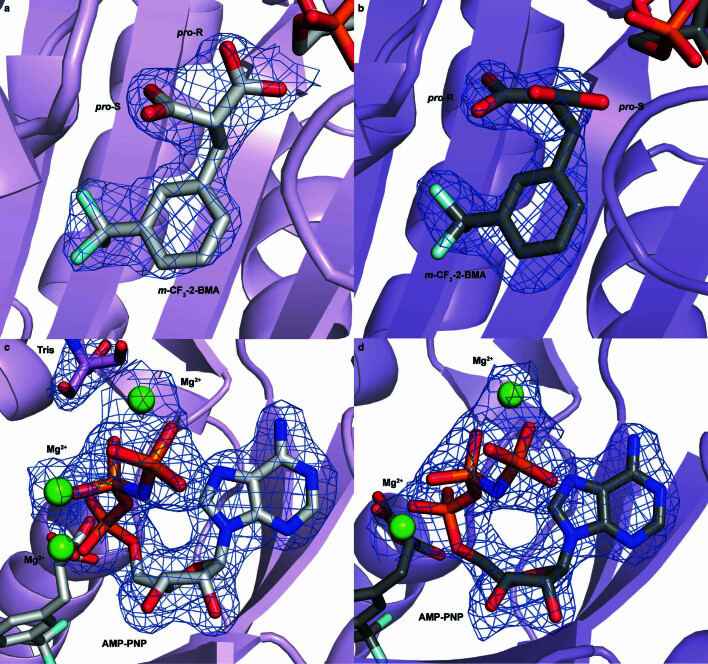
Ligand electron densities of *m*-CF_3_-2-BMA (**18**) and AMP-PNP bound to *Ma*FRSA. Electron density shown from *2F*_*0*_*-F*_*c*_ map contoured at *1σ* for *m*-CF_3_-2-BMA bound to chain A (**a**) and chain B (**b**) and AMP-PNP bound to chain A (**c**) and chain B (**d**).

To better understand how 2-BMA substrates are accommodated by the *Ma*FRSA active site, we solved the crystal structure of *Ma*FRSA bound to both *m*-CF_3_-2-BMA and the non-hydrolysable ATP analogue adenosine 5′-(β,γ-imido)triphosphate (AMP-PNP). The structure was refined at 1.8 Å resolution with clear substrate density for *m*-CF_3_-2-BMA visible in the electron density map (Extended Data Fig. 3 and Supplementary Table 4). *Ma*FRSA crystallized with two protein chains in the asymmetric unit and an overall architecture resembling previously published PylRS structures (Fig. 5a)^37,60,61^. The two protein chains in the asymmetric unit are not identical and interact with different orientations of *m*-CF_3_-2-BMA (Fig. 5b). One orientation of *m*-CF_3_-2-BMA (chain A, light purple) mimics that of l-pyrrolysine (Pyl) bound to *Mm*PylRS^37^ and would result in adenylation of the *pro*-*R* carboxylate (Fig. 5c); the other orientation (chain B, dark purple) would result in adenylation of the *pro*-*S* carboxylate (Fig. 5d).

**Extended Data Fig. 4:**
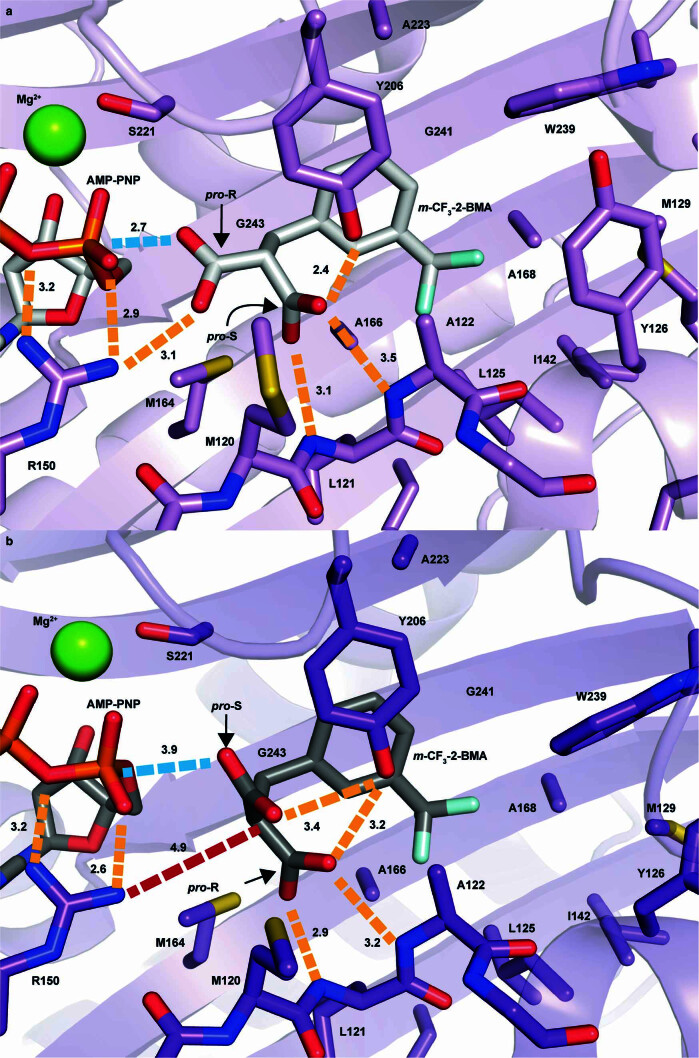
Expanded view of *Ma*FRSA recognition of *m*-CF_3_-2-BMA (**18**). Additional residues comprising the active site shown in chain A (light purple, **a**) and chain B (dark purple, **b**). Dashed lines indicate hydrogen-bonding interactions (orange), path of nucleophilic attack (blue), or missing hydrogen bonds (red).

Regardless of orientation, discrete networks of hydrogen bonds are used by *Ma*FRSA to discriminate between the *pro*-*R* and *pro*-*S* carboxylates of *m*-CF_3_-2-BMA. In chain A, the *pro*-*S* carboxylate accepts a hydrogen bond from the backbone amides of Leu121 and Ala122 and the phenolic -OH of Tyr206 (Fig. 5c and Extended Data Fig. 4a). A hydrogen bond from the Arg150 guanidinium positions the *pro*-*R* carboxylate for nucleophilic attack with a distance of 2.7 Å between the carboxy oxygen and the α-phosphorus of AMP-PNP. In chain B, the orientation of *m*-CF_3_-2-BMA is flipped relative to the conformation bound to chain A (Fig. 5d and Extended Data Fig. 4b). The *pro*-*R* carboxylate accepts a hydrogen bond from the backbone amides of Leu121 and Ala122 and the phenolic -OH of Tyr206, as seen for the *pro*-*S* carboxylate in chain A. However, in chain B, the *pro*-*S* carboxylate is rotated away from AMP-PNP and towards Tyr206, resulting in loss of the hydrogen bond to Arg150 and a longer distance of 3.9 Å between the carboxy oxygen and the α-phosphorus of AMP-PNP. RNAse A analysis of *Ma*-tRNA^Pyl^ acylation by *m*-CF_3_-2-BMA showed more than two peaks of identical mass (Fig. 4) that likely correspond to the two diastereomeric pairs formed from attack of the tRNA 2′- or 3′-hydroxy group on the activated *pro*-*R* or *pro*-*S* carboxylate. More than two peaks with identical mass were also observed as RNAse A digestion products in *Ma*-tRNA^Pyl^ acylation reactions of other *m-*substituted 2-BMAs (Fig. 4b). While the *m*-CF_3_-2-BMA conformation in chain A seems to be more favourably positioned for catalysis, suggesting that the *pro*-*R* carboxylate is acylated preferentially, the appearance of more than two peaks in the RNAse A assay suggests that both conformations are catalytically competent.

**Extended Data Fig. 5:**
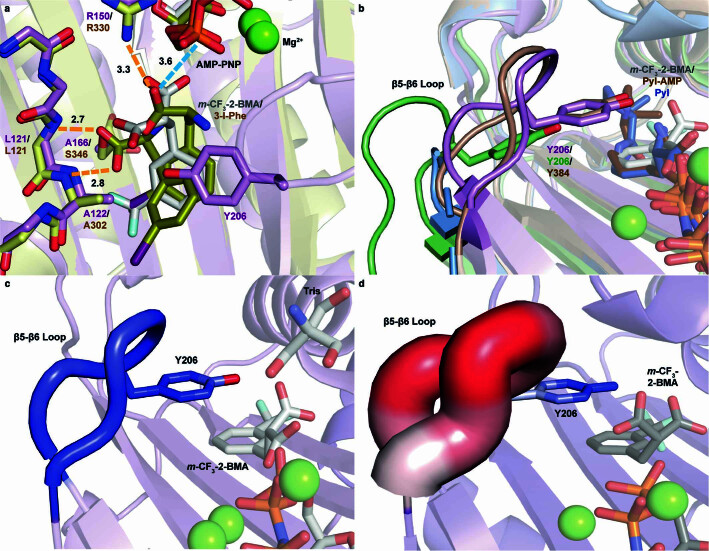
Structural recognition of *m*-CF_3_-2-BMA (**18**) by *Ma*FRSA and β7-β8 loop positioning in comparison with published PylRS structures. **a**, Alignment of *Mm*IFRS (N346S/C348Q, yellow, PDB: 4TQD) bound to 3-iodo-L-phenylalanine and AMP-PNP and *Ma*FRSA bound to *m*-CF_3_-2-BMA (**18**) and AMP-PNP chain A (light purple) illustrating similar interactions between substrate carboxylate and backbone amides. **b**, The flexible β7-β8 loop ranges between unstructured, an open conformation, and a closed conformation across PylRS structures. *Ma*FRSA bound to *m*-CF_3_-2-BMA and AMP-PNP (light purple), wild-type *Ma*PylRS apo (green, PDB: 6JP2), wild-type *Mm*PylRS bound to pyrrolysine and AMP-PNP (blue, PDB: 2ZCE), and wild-type *Mm*PylRS bound to pyrrolysyl-adenylate (brown, PDB: 2Q7H). Chain A (light purple, **c**) exhibits lower B-factors in the β5-β6 loop than chain B (dark purple, **d**) indicated by dark blue and thin ribbon for lower B-factors and dark red and thick ribbon for higher B-factors.

As anticipated, the non-reactive, *pro*-*S* carboxylate of *m*-CF_3_-2-BMA is recognized by *Ma*FRSA chain A using interactions that are distinct from those used by *Mm*PylRS to recognize the Pyl α-amine. In the *Mm*PylRS/Pyl/AMP-PNP complex (PDB: 2ZCE)^37^, the Pyl α-amine is recognized by water-mediated hydrogen bonds to the backbone amides of Leu301 and Ala302 and the side-chain carbonyl of Asn346 rather than by direct hydrogen bonds to the backbone amides of Leu121 and Ala122, as seen for the recognition of the non-reacting carboxylate of *m*-CF_3_-2-BMA by both chains of *Ma*FRSA (Fig. 5e). The interactions used to recognize the non-reacting carboxylate of *m*-CF_3_-2-BMA are, however, similar to those used to recognize the single carboxylate of diverse α-amino acids by other PylRS variants with mutations at Asn166/Asn346 (*Ma*/*Mm* numbering). For example, the structures of *Mm*PylRS variants with mutations at Asn346, such as *Mm*IFRS and *Mm*BtaRS bound to 3-iodo-l-phenylalanine (3-I-F, PDB: 4TQD)^58^ and 3-benzothienyl-l-alanine (Bta, PDB: 4ZIB)^62^, respectively, show the substrate bound with the carboxylate directly hydrogen bonded to the Leu301 and Ala302 backbone amides, as seen for *m*-CF_3_-2-BMA bound to *Ma*FRSA (Fig. 5f and Extended Data Fig. 5a). In these cases, the bound water seen in the *Mm*PylRS/Pyl/AMP-PNP complex is either absent or displaced. The mutation of Asn166/Asn346 may destabilize the water-mediated hydrogen bonding between the substrate α-amine and backbone amides seen in WT PylRS and promote alternative direct hydrogen bonding of a substrate carboxylate to backbone amides, as seen in *Ma*FRSA, *Mm*IFRS and *Mm*BtaRS.

The largest differences between *Ma*FRSA and other reported PylRS structures are localized in a six-residue loop that straddles β-strands β5 and β6 and contains the active-site residue Tyr206. In the *Mm*PylRS/Pyl/AMP-PNP (PDB: 2ZCE)^37^ and WT *Ma*PylRS apo structures (PDB: 6JP2)^60^, the β5–β6 or corresponding loop is either unstructured or in an open conformation, positioning Tyr206/Tyr384 away from the active site, respectively (Extended Data Fig. 5b). Among WT PylRS structures, the Tyr206/Tyr384-containing loop exists in the closed conformation only in the structure of *Mm*PylRS bound to the reaction product Pyl-adenylate (PDB: 2Q7H)^61^. In this structure, Tyr384 accepts and donates a hydrogen bond to the Pyl-adenylate α-amine and pyrrole nitrogen, respectively, and forms a hydrophobic lid over the active site. In both chains of substrate-bound *Ma*FRSA, the non-reacting carboxylate of *m*-CF_3_-2-BMA forms similar hydrogen bonds with Tyr206. These hydrogen bonds effectively close the β5–β6 loop to form a hydrophobic lid over the active site, which may contribute to the high acylation activity observed for *m*-CF_3_-2-BMA with *Ma*FRSA. We note that the β5–β6 loop in chain A exhibits lower *B*-factors than in chain B, indicating tighter binding and providing further evidence that chain A represents the more active binding mode of *m*-CF_3_-2-BMA (Extended Data Fig. 5c,d). The two alternative poses of *m*-CF_3_-2-BMA in chains A and B correspond to a rotation of ~120° about the Cα–Cβ bond of the substrate that largely maintains interactions with the *m*-CF_3_-2-BMA side chain but alters the placement of the reacting carboxylate. This observation emphasizes the dominant stabilizing role of the main-chain hydrogen bonds provided by the backbone amides of Leu121 and Ala122.

### In vitro translation initiation via codon skipping

We performed in vitro translation experiments to verify that the uniquely acylated derivatives of *Ma*-tRNA^Pyl^ produced using the *Ma*PylRS variants are effectively shuttled to and accommodated by the *E. coli* ribosome. Whereas the *E. coli* initiator tRNA^fMet^ has been engineered into a substrate for *Mj*TyrRS variants to introduce non-canonical l-α-amino acids at the protein N terminus^55^, *Ma*-tRNA^Pyl^ lacks the key sequence elements for recognition by *E. coli* initiation factors, precluding its use for initiation in vivo^39,63^. It has been reported^64^ that in the absence of methionine, in vitro translation can begin at the second codon of the mRNA template, a phenomenon we refer to as ‘codon skipping’. We took advantage of codon skipping and a commercial in vitro translation kit to evaluate whether *Ma*-tRNA^Pyl^ enzymatically charged with monomers **13**–**15** would support translation initiation.

**Fig. 6:**
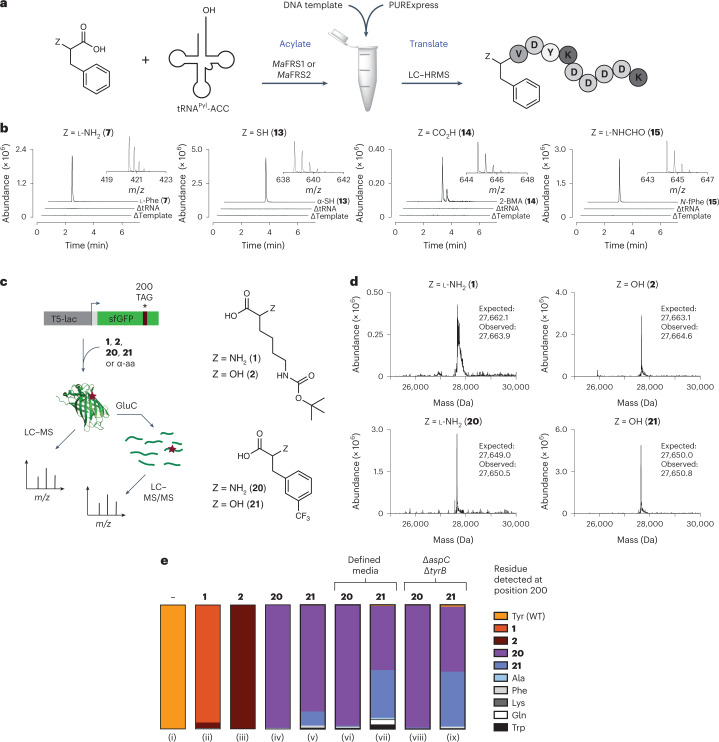
In vitro and in vivo incorporation of novel expanded monomer selection. **a**, Workflow for in vitro translation via codon skipping. **b**, EICs and mass spectra of peptide products obtained using *Ma*-tRNA^Pyl^-ACC charged with monomers **7** and **13**–**15** by *Ma*FRS1 (**7** and **15**) or *Ma*FRS2 (**13** and **14**). The insets show the mass spectra of the major ions used to generate the EIC of the translated peptide initiated with the indicated monomer. The expected (exp) and observed (obs) *m*/*z* peaks in the mass spectra are as follows: l-Phe (**7**) (M + 3H): exp: 420.51906, obs: 420.52249; α-SH **13** (M + 2H): exp: 638.75554, obs: 638.75614; 2-BMA (**14**) (M + 2H): exp: 644.76442, obs: 644.76498; *N*-formyl-l-Phe (*N*-fPhe) **15** (M + 2H): exp: 644.27242, obs: 644.27167. **c**, Workflow for the in vivo incorporation of monomers **1**, **2**, **20** and **21** at position 200 of sfGFP. **d**, Intact protein mass spectra of sfGFP variants purified from DH10B cells co-expressing *Ma*PylRS (top) or *Ma*FRSA (bottom) in the presence of 1 mM BocK (**1**), α-OH-BocK (**2**), *m*-trifluoromethylphenylalanine (**20**) or α-OH-*m*-trifluoromethylphenylalanine (**21**). **e**, Fidelity (%) of sfGFP containing the indicated residue at position 200 when expressed in *E. coli* DH10B (i–vii) or DH10B ∆*aspC* ∆*tyrB* (viii and ix) using either terrific broth (i–v, viii and ix) or a defined media lacking glutamate (vi and vii). The fidelity was determined as described in Methods and Supplementary Fig. 39. Source data

To avoid competition with release factor 1 at UAG codons, the anticodon of *Ma*-tRNA^Pyl^ was mutated to ACC (*Ma*-tRNA^Pyl^-ACC) to recode a glycine GGT codon at position 2 in the mRNA template. To maximize yields of acyl-tRNA, we increased the aaRS/tRNA ratio to 1:2 (monomers **7**, **14** and **15**) or 1:1 (monomer **13**), extended the incubation time to 3 h and used the most active *Ma*FRS*x* variant for each monomer. These modifications resulted in acyl-tRNA yields of 79% (**7,** *Ma*FRS1), 13% (**13**, *Ma*FRS2), 85% (**14**, *Ma*FRS2) and 82% (**15**, *Ma*FRS1). The acylated *Ma*-tRNA^Pyl^-ACC was added with a DNA template encoding a short MGV-FLAG peptide (MGVDYKDDDDK; Fig. 6a) to a commercial in vitro translation kit (PURExpress Δ (aa, tRNA)). When methionine was excluded from the reaction mixture, translation was initiated at the second position by skipping the start codon to produce peptides with the sequence XVDYKDDDDK (X = **7**, **13**–**15**). Following FLAG-tag enrichment, LC–HRMS confirmed the initiation with monomers **7** and **13**–**15** (Fig. 6b). When the mass corresponding to the *m*/*z* = M + 2H (**13**–**15**) or *m*/*z* = M + 3H (**7**) charge state of each peptide was extracted from the total ion chromatogram (TIC), a clear peak was observed for each peptide. No such peak was observed in reactions that lacked either the DNA template or acyl-tRNA, confirming templated ribosomal initiation with α-thio acid **13**, 2-benzylmalonic acid (**14**) and *N*-formyl-l-α-amino acid **15**. Two peaks of identical mass were observed in the extracted ion chromatogram (EIC) when translation was initiated with 2-benzylmalonic acid (**14**), which we assigned to diastereomeric peptides resulting from acylation at either the *pro*-*S* or *pro*-*R* carboxylate. Combined with the multiple peaks present in the RNAse A assay with 2-benzylmalonic acids **17**, **18** and **19** (Fig. 4b and Extended Data Fig. 1), as well as the two *m*-CF_3_-2-BMA conformations observed in the structure of *Ma*FRSA (Fig. 5b), the two peptide products of identical mass generated in the in vitro translation reactions imply that *Ma*FRS*x* enzymes can effectively acylate either of the two prochiral carboxylates of a malonic acid substrate.

### In vivo translation of ester-amide heteropolymers

We next sought to evaluate whether the ability of *Ma*PylRS and *Ma*FRSA to acylate tRNA with an expanded subset of monomers in vitro would support the translation of backbone-modified protein heteropolymers in vivo. As the yields of tRNAs acylated in vitro with *N*-methyl-α-amino acids and α-thio acids were low (Fig. 3), *N*-formyl-α-amino acids lack an appropriate nucleophile and the requirements for intra-PTC bond formation by α-carboxy (malonic) acids are unknown, we focused on the in vivo incorporation of α-OH-BocK (**2**) and α-OH-*m*-trifluoromethyl-l-phenylalanine (α-OH-*meta*-CF_3_-Phe, **21**).

**Extended Data Fig. 6:**
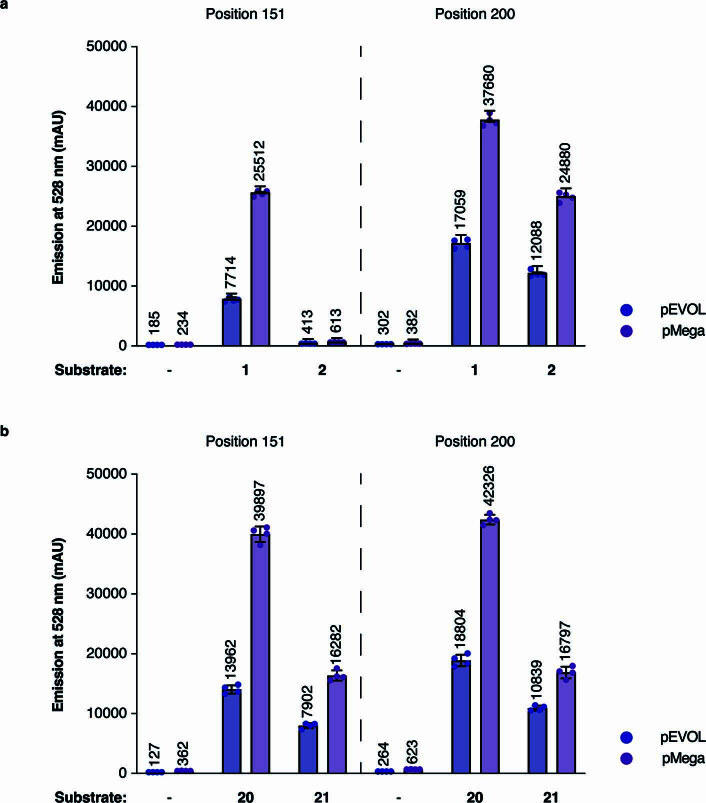
Plate reader analysis of sfGFP expression in DH10B *E. coli* with *n* = 4 replicates. Emission at 528 nm after 24 h sfGFP expression in *E. coli* DH10B cells harboring pEVOL or pMega plasmids encoding **a**, *Ma*PylRS and *Ma-*tRNA^Pyl^ in the presence of 0 or 1 mM BocK (**1**) or α-OH BocK (**2**) or **b**, *Ma*FRSA and *Ma-*tRNA^Pyl^ in the presence of 0 or 1 mM *m*-CF_3_-L-phenylalanine (**20**) or α-OH *m*-CF_3_- phenylalanine (**21**). Source data

First, we modified established pUltra plasmids^65^ for aaRS/tRNA expression by adding a *lac* operator between the tRNA promoter and coding region to make tRNA expression inducible^66^. The resulting pMega plasmids were more easily propagated than the pUltra plamids^67^. Using the resulting pMega or modified pEVOL (ref. ^68^) plasmids, we performed experiments in *E. coli* DH10B cells transformed with one of two superfolder green fluorescent protein (sfGFP) reporter plasmids (pET22b-sfGFP-200TAG or pET22b-sfGFP-151TAG; Supplementary Fig. 36) encoding *Ma-*tRNA^Pyl^ and either *Ma*PylRS or *Ma*FRSA. The growth was supplemented with 1 mM BocK (**1**), α-OH-BocK (**2**), *m*-CF_3_-Phe (**20**) or α-OH-*m-*CF_3_-Phe (**21**), and the emission at 528 nm, close to the *λ*_max_ for sfGFP, was assessed after 24 h. In most cases, DH10B cells harbouring a pMega plasmid produced two- to threefold higher levels of sfGFP fluorescence than those harbouring a pEVOL plasmid (Extended Data Fig. 6). In all but one case, the α-OH monomers led to approximately 1.5- to 2-fold lower sfGFP fluorescence than the α-NH_2_ monomers. The highest levels of sfGFP fluorescence were observed in those cases in which α-NH_2_ or α-OH monomers were encoded at position 200.

Preparative-scale growth was conducted using pET22b-sfGFP-200TAG and pMega-*Ma*PylRS or pMega-*Ma*FRSA, the top-performing plasmids in the plate reader assay, and the sfGFP products were isolated by metal affinity chromatography (Supplementary Fig. 37). As observed previously with WT *Mm*PylRS, *E. coli* DH10B cells expressing *Ma*PylRS and grown in the presence of 1 mM BocK (**1**) or α-OH-BocK (**2**) expressed an sfGFP variant whose mass corresponded to the presence of a single BocK side chain (Fig. 6d); analogous cells expressing *Ma*FRSA and grown in the presence of 1 mM *m*-CF3-Phe (**20**) or α-OH-*m-*CF_3_-Phe (**21**) expressed an sfGFP variant whose mass corresponded to the presence of a single trifluoromethylphenylalanine side chain (Fig. 6d). Although the intact masses of the proteins grown in the presence of BocK (**1**) and *m-*CF_3_-Phe (**20**) differed from those grown in the presence of α-OH-BocK (**2**) or α-OH-*m-*CF_3_-Phe (**21**), the resolution was insufficient to unequivocally establish the ester/amide ratio. To characterize the products more rigorously, we digested the isolated sfGFP variants with GluC (Supplementary Fig. 38) and analysed the products by LC with tandem MS (LC–MS/MS; Fig. 6e(i)–(v) and Supplementary Fig. 39). This analysis revealed that the sfGFP produced in the presence of α-OH BocK (**2**) contained virtually only an ester linkage at position 200, whereas the sfGFP product generated in the presence of α-OH-*m-*CF_3_-Phe (**21**) contained an ester/amide ratio of 11:85 (Fig. 6e and Supplementary Table 5).

Certain α-hydroxy acids can be metabolized in *E. coli* to α-amino acids via a two-step oxidation–transamination process. Indeed, in classic work^21^, a DH10B strain lacking the transaminase genes *aspC* and *tyrB* was required to detect cytosolic accumulation of the α-OH analogue of tyrosine, 3-(4-hydroxyphenyl)lactic acid^21^. We thus repeated the preparative-scale growth of DH10B ∆*aspC* ∆*tyrB* harbouring pET22b-sfGFP-200TAG and pMega-*Ma*FRSA supplemented with 1 mM *m-*CF_3_-Phe (**20**) or α-OH-*m-*CF_3_-Phe (**21**). For comparison, we also performed growth in DH10B using a defined media lacking glutamate, the amine donor for AspC and TyrB, in an attempt to minimize transaminase activity. The results of intact mass analysis of sfGFP isolated from DH10B ∆*aspC* ∆*tyrB* growth supplemented with α-OH-*m-*CF_3_-Phe (**21**) were again consistent with the expected products (Supplementary Figs. 37 and 39). Both strategies improved the fraction of ester product, as judged by GluC digestion of the intact isolated sfGFP; use of the defined media led to a 39:52 ester/amide ratio, whereas use of DH10B ∆*aspC* ∆*tyrB* led to an ester/amide ratio of 44:52 (Fig. 6e, Supplementary Fig. 39 and Supplementary Table 5). These studies reveal that while *Ma*FRSA is sufficiently active in vivo to support the biosynthesis of a sequence-defined hetero-oligomer, more complex *E. coli* strains^69,70^ or alternative organisms^71^ may be required to generate ribosomal products containing multiple esters in high yield.

## Discussion

Expanding and reprogramming the genetic code for the templated biosynthesis of sequence-defined heteropolymers demands orthogonal aaRS/tRNA pairs that accept non-l-α-amino acid substrates. Over 50 years ago, the PTC of the *E. coli* ribosome was reported to tolerate an α-hydroxy substituent in place of the α-amine of phenylalanine and biosynthesize a polyester in vitro^3^. More recent in vitro studies have broadened the scope of WT PTC reactivity to include diverse nucleophilic heteroatoms in place of the α-amine^13,14,16,17^. However, with the exception of substrates carrying an α-hydroxy substituent^21–24,27^, none of these non-l-α-amino acid monomers is a substrate for any known orthogonal aaRS. The challenge is that most aaRSs of known structure simultaneously engage both the substrate α-amine and α-carboxylate moieties via multiple main-chain and side-chain hydrogen bonds to position the α-carboxylate for adenylation and acylation. These well-conserved hydrogen bond networks complicate the engineering of new enzymes that accept substrates with alternative amine and/or carboxylate orientations or conformations (such as β-amino acids or aminobenzoic acids), or those whose α-substituents are large and/or electrostatically distinctive (such as malonates, α-thio acids and *N*-acyl-α-amino acids).

The water-mediated hydrogen bond network in the active site of *Mm*PylRS has been shown to facilitate the recognition of substrates with conservative substitutions (α-OH, α-H and α-NHCH_3_) of the α-amino group^22^. Here we have expanded the scope of monomers recognized and processed by PylRS variants to include α-thio acids and *N*-formyl-l-α-amino acids, as well as those that carry an α-carboxy functional group in place of the α-amine, that is, prochiral malonic acids with protein-like side chains. Monomers containing α-thio, *N*-formyl-l-α-amino and α-carboxy substituents in place of the α-amine can be incorporated into polypeptides at the N terminus by the native *E. coli* translational apparatus; those with an α-hydroxy substituent can be introduced into proteins in vivo, albeit in a side-chain- and position-specific manner. Ester-containing biopolymers produced at scale could generate biomaterials that change shape and self-cleave in a pH- and/or environment-selective manner.

Although thioesters and malonic acids are ubiquitous intermediates in polyketide and fatty acid biosynthesis^72,73^, as far as we know, aaRS enzymes that act on α-thio or α-carboxy acids were previously unknown, and tRNAs acylated with a polyketide precursor represent distinct chemical species. Such tRNAs could forge a link between ribosomal translation and the assembly-line PKSs^74^ responsible for protein and polyketide biosynthesis, respectively. Combined with synthetic genomes^69,70^, ribosomes capable of carbon–carbon bond formation would set the stage for the template-driven biosynthesis of unique hybrid biomaterials and sequence-defined polyketide-peptide oligomers, such as those produced by PKS-non-ribosomal peptide synthetase biosynthetic modules.

## Methods

### Expression and purification of *Ma*PylRS, *Ma*FRS1, *Ma*FRS2 and *Ma*FRSA for biochemical analysis

The plasmids used to express WT *Ma*PylRS (pET32a-*Ma*PylRS) and *Ma*FRS1 (pET32a-*Ma*FRS1) were constructed by inserting synthetic double-stranded DNA (dsDNA) fragments (Supplementary Table 1) into the NdeI-NdeI cut sites of a pET32a vector using the Gibson method^75^. pET32a-*Ma*FRS2 and pET32a-*Ma*FRSA were constructed from pET32a-*Ma*FRS1 using a Q5 Site-Directed Mutagenesis Kit (NEB). Primers RF31 and RF32, and RF32 and RF33 (Supplementary Table 1) were used to construct pET32a-*Ma*FRS2 and pET32a-*Ma*FRSA, respectively. The sequences of the plasmids spanning the inserted regions were confirmed via Sanger sequencing at the University of California Berkeley DNA Sequencing Facility using T7 forward and reverse (T7 F and T7 R) primers (Supplementary Table 1), and the complete sequence of each plasmid was confirmed by the Massachusetts General Hospital Center for Computational and Integrative Biology DNA Core.

Chemically competent cells were prepared by following a modified published protocol^76^. Briefly, 5 ml Luria-Bertani (LB) medium was inoculated using a freezer stock of BL21-Gold (DE3)pLysS cells. The following day, 50 ml LB was inoculated with 0.5 ml of the culture from the previous day and incubated at 37 °C with shaking at 200 r.p.m. until the culture reached an optical density at 600 nm (OD_600_) in the range of 0.3–0.4. The cells were collected by centrifugation at 4,303*g* for 20 min at 4 °C. The cell pellet was resuspended in 5 ml of sterile filtered TSS solution (10% (wt/v) polyethylene glycol 8,000, 30 mM MgCl_2_, 5% (v/v) dimethylsulfoxide in 25 g l^−1^ LB). The chemically competent cells were portioned into 100 µl aliquots in 1.5 ml microcentrifuge tubes, flash frozen in liquid N_2_ and stored at −80 °C until use. The following protocol was used to transform plasmids into chemically competent cells: 20 µl KCM solution (500 mM KCl, 150 mM CaCl_2_ and 250 mM MgCl_2_) was added to a 100 µl aliquot of cells held on ice along with approximately 200 ng of the requisite plasmid and water to a final volume of 200 µl. The cells were incubated on ice for 30 min and then heat-shocked by placing them for 90 s in a water bath heated to 42 °C. Immediately after heat shock, the cells were placed on ice for 2 min, after which 800 µl LB was added. The cells were then incubated at 37 °C with shaking at 200 r.p.m. for 60 min. The cells were plated onto LB agar plates with the appropriate antibiotic and incubated overnight at 37 °C.

The plasmids used to express WT *Ma*PylRS, *Ma*FRS1, *Ma*FRS2, and *Ma*FRSA were transformed into BL21-Gold (DE3)pLysS chemically competent cells and plated onto LB agar plates supplemented with 100 µg ml^−1^ carbenicillin. Colonies were picked the following day and used to inoculate 10 ml LB supplemented with 100 µg ml^−1^ carbenicillin. The cultures were incubated overnight at 37 °C with shaking at 200 r.p.m. The following day, the 10 ml cultures were used to inoculate 1 l LB supplemented with 100 µg ml^−1^ carbenicillin in 4-l baffled Erlenmeyer flasks. The cultures were incubated at 37 °C with shaking at 200 r.p.m. for 3 h until they reached an OD_600_ of 0.6–0.8. At this point, isopropyl β-d-1-thiogalactopyranoside (IPTG) was added to a final concentration of 1 mM and the incubation was continued for 6 h at 30 °C with shaking at 200 r.p.m. The cells were collected by centrifugation at 4,303*g* for 20 min at 4 °C and the cell pellets stored at −80 °C until the expressed protein was purified as described below.

The following buffers were used for protein purification: wash buffer: 50 mM sodium phosphate (pH 7.4), 500 mM NaCl, 20 mM β-mercaptoethanol, 25 mM imidazole; elution buffer: 50 mM sodium phosphate (pH 7.4), 500 mM NaCl, 20 mM β-mercaptoethanol, 100 mM imidazole; storage buffer: 100 mM HEPES-K (pH 7.2), 100 mM NaCl, 10 mM MgCl_2_, 4 mM dithiothreitol (DTT), 20% (v/v) glycerol. One cOmplete Mini EDTA-free protease inhibitor tablet was added to the wash and elution buffers immediately before use. To isolate protein, cell pellets were resuspended in wash buffer (5 ml per g cells). The resultant cell paste was lysed at 4 °C by sonication (Branson Sonifier 250) over 10 cycles of 30 s sonication followed by 30 s manual swirling. The lysate was centrifuged at 4,303*g* for 10 min at 4 °C to separate the soluble and insoluble fractions. The soluble lysate was incubated at 4 °C with 1 ml Ni nitrilotriacetic acid (Ni-NTA) agarose resin (washed with water and equilibrated with wash buffer) for 2 h. The lysate–resin mixture was added to a 65 g RediSep disposable sample load cartridge (Teledyne ISCO) and allowed to drain at room temperature. The protein-bound Ni-NTA agarose resin was then washed three times with 10 ml aliquots of wash buffer. The protein was eluted from the Ni-NTA agarose resin by rinsing the resin three times with 10 ml elution buffer. The elution fractions were pooled and concentrated using a 10 kDa molecular weight cut-off (MWCO) Amicon Ultra-15 centrifugal filter unit (4,303*g*, 4 °C). The protein was then buffer-exchanged into the storage buffer using the same centrifugal filter unit until the imidazole concentration was less than 5 µM. The protein was dispensed into 20 µl single-use aliquots and stored at −80 °C for up to 8 months. The protein concentration was measured using the Bradford assay^77^. The yields were between 8 and 12 mg l^−1^. Proteins were analysed by SDS–PAGE (Supplementary Fig. 2) using Any kD Mini-PROTEAN TGX precast protein gels (BioRad). The gels were run at 200 V for 30 min.

Proteins were analysed by LC–MS to confirm their identity (Supplementary Fig. 2). The samples analysed by MS were resolved using a Poroshell StableBond 300 C8 column (2.1 mm × 75 mm, 5 µm; Agilent, 660750-906) with a 1290 Infinity II ultra-high-performance liquid chromatograph (UHPLC; Agilent, G7120AR). The mobile phases used for separation were 0.1% formic acid in water (mobile phase A) and 100% acetonitrile (mobile phase B), and the flow rate was 0.4 ml min^−1^. After an initial hold at 5% B for 0.5 min, the proteins were eluted using a linear gradient from 5% to 75% B for 9.5 min, a linear gradient from 75% to 100% B for 1 min, a hold at 100% B for 1 min, a linear gradient from 100% to 5% B for 3.5 min and finally a hold at 5% B for 4.5 min. The protein masses were analysed by LC–HRMS using a 6530 Q-TOF AJS-ESI (Agilent, G6530BAR) instrument. The following parameters were used: gas temperature = 300 °C, drying gas flow = 12 l min^−1^, nebulizer pressure = 35 psi, sheath gas temperature = 350 °C, sheath gas flow = 11 l min^−1^, fragmentor voltage = 175 V, skimmer voltage = 65 V, peak-to-peak voltage (*V*_pp_) = 750 V, capillary voltage (*V*_cap_) = 3,500 V, nozzle voltage = 1,000 V and collection rate = 3 spectra s^−1^.

Analytical size exclusion chromatography (SEC; Supplementary Fig. 2) was performed on an ÄKTA Pure 25 instrument. A flow rate of 0.5 ml min^−1^ was used for all steps. A Superdex 75 Increase 10/300 GL column (stored and operated at 4 °C) was washed with 1.5 column volumes of degassed, sterile, filtered MilliQ water. The column was equilibrated in 1.5 column volumes of SEC buffer: 100 mM HEPES (pH 7.2), 100 mM NaCl, 10 mM MgCl_2_, 4 mM DTT. Approximately 800 µg of protein in 250 µl SEC buffer was loaded into a 500 µl capillary loop. The sample loop was washed with 2.0 ml SEC buffer as the sample was injected onto the column. The sample was eluted in 1.5 column volumes of SEC buffer and analysed by UV spectrophotometry at 280 nm.

### Transcription and purification of tRNAs

The DNA template used for transcribing *Ma-*tRNA^Pyl^ (ref. ^39^) was prepared by annealing and extending the single-stranded DNA oligonucleotides *Ma*-PylT-F and *Ma*-PylT-R (2 mM; Supplementary Table 1) using OneTaq 2x Master Mix (NEB). The annealing and extension process was performed using the following protocol on a thermocycler (BioRad, C1000 Touch): 94 °C for 30 s, 30 cycles of 94 °C for 20 s, 53 °C for 30 s and 68 °C for 60 s, and finally 68 °C for 300 s. Following the extension, the reaction mixture was supplemented with sodium acetate (pH 5.2) to a final concentration of 300 mM, washed once with 1:1 (v/v) acid phenol–chloroform, twice with chloroform and the dsDNA product precipitated by addition of ethanol to a final concentration of 71%. The pellet was resuspended in water and the concentration of dsDNA determined using a NanoDrop ND-1000 device (Thermo Scientific). The template starts with a single C preceding the T7 promoter, which increases the yields of T7 transcripts^78^. The penultimate residue of *Ma*-PylT-R carries a 2′-methoxy modification, which reduces non-templated nucleotide addition by T7 RNA polymerase during in vitro transcription^79^.

*Ma*-tRNA^Pyl^ was transcribed in vitro using a modified version of a published procedure^80^. Transcription reactions (25 µl) contained the following components: 40 mM Tris-HCl (pH 8.0), 100 mM NaCl, 20 mM DTT, 2 mM spermidine, 5 mM ATP, 5 mM cytidine triphosphate, 5 mM guanosine triphosphate, 5 mM uridine triphosphate, 20 mM guanosine monophosphate, 0.2 mg ml^−1^ bovine serum albumin, 20 mM MgCl_2_, 12.5 ng µl^−1^ DNA template and 0.025 mg ml^−1^ T7 RNA polymerase. The reaction mixtures were incubated at 37 °C in a thermocycler for 3 h. Four 25 µl reactions were pooled, and then sodium acetate (pH 5.2) was added to a final concentration of 300 mM in a volume of 200 µl. The transcription reaction mixtures were extracted once with 1:1 (v/v) acid phenol–chloroform, washed twice with chloroform and the tRNA product precipitated by adding ethanol to a final concentration of 71%. After precipitation, the tRNA pellet was resuspended in water and incubated with 8 U of RQ1 RNAse-free DNAse (Promega) at 37 °C for 30 min according to the manufacturer’s protocol. The tRNA was then washed with acid phenol–chloroform and chloroform as described above, precipitated and resuspended in water. To remove small molecules, the tRNA was further purified using a Micro Bio-Spin P-30 gel column, Tris buffer (RNase-free; BioRad) after first exchanging the column buffer with water according to the manufacturer’s protocol. The tRNA was precipitated once more, resuspended in water, quantified using a NanoDrop ND-1000 device, aliquoted and stored at −20 °C.

tRNA was analysed by urea–PAGE (Supplementary Fig. 2**)** using 10% Mini-PROTEAN Tris-borate-ethylenediaminetetraacetic acid-urea gel (BioRad). The gels were run at 120 V for 30 min and then stained with SYBR Safe gel stain (Thermo Fisher) for 5 min before imaging. *Ma*-tRNA^Pyl^ was analysed by LC–MS to confirm its identity. Samples were resolved on an ACQUITY UPLC BEH C18 column (130 Å, 1.7 µm, 2.1 mm × 50 mm, 60 °C; Waters, 186002350) using an ACQUITY UPLC I-Class PLUS instrument (Waters, 186015082). The mobile phases used were 8 mM triethylamine, 80 mM hexafluoroisopropanol and 5 µM EDTA (free acid) in 100% MilliQ water (mobile phase A) and 4 mM triethylamine, 40 mM hexafluoroisopropanol and 5 µM EDTA (free acid) in 50% MilliQ water–50% methanol (mobile phase B). The analysis was performed at a flow rate of 0.3 ml min^−1^ and began with mobile phase B at 22%, increasing linearly to 40% B over 10 min, followed by a linear gradient from 40% to 60% B for 1 min, a hold at 60% B for 1 min, a linear gradient from 60% to 22% B over 0.1 min and then a hold at 22% B for 2.9 min. The mass of the RNA was analysed by LC–HRMS with a Xevo G2-XS Tof instrument (Waters, 186010532) in negative ion mode with the following parameters: capillary voltage = 2,000 V, sampling cone = 40, source off-set = 40, source temperature = 140 °C, desolvation temperature = 20 °C, cone gas flow = 10 l h^−1^, desolvation gas flow = 800 l h^−1^ and collection rate = 1 spectrum s^−1^. The expected masses of the oligonucleotide products were calculated using the AAT Bioquest RNA Molecular Weight Calculator. Deconvoluted mass spectra were obtained using the MaxEnt software (Waters).

### Procedure for RNAse A assays

The reaction mixtures (25 µl) used to acylate tRNA contained the following components: 100 mM HEPES-K (pH 7.5), 4 mM DTT, 10 mM MgCl_2_, 10 mM ATP, 0–10 mM substrate, 0.1 U *E. coli* inorganic pyrophosphatase (NEB), 25 µM *Ma*-tRNA^Pyl^ and 2.5 µM enzyme (*Ma*PylRS, *Ma*FRS1, *Ma*FRS2 or *Ma*FRSA). The reaction mixtures were incubated at 37 °C in a dry air incubator for 2 h. The tRNA samples from the enzymatic acylation reactions were quenched with 27.5 µl RNAse A solution (1.5 U µl^−1^ RNAse A (MilliporeSigma) and 200 mM sodium acetate, pH 5.2) and incubated for 5 min at room temperature. The proteins were then precipitated by the addition of 50% trichloroacetic acid (Sigma-Aldrich) to a final concentration of 5%. After precipitating protein at −80 °C for 30 min, insoluble material was removed by centrifugation at 21,300*g* for 10 min at 4 °C. The soluble fraction was then transferred to autosampler vials, kept on ice until immediately before LC–MS analysis and returned to ice immediately afterwards.

The samples analysed by MS were resolved using a Zorbax Eclipse XDB-C18 RRHD column (2.1 mm × 50 mm, 1.8 μm, room temperature; Agilent, 981757-902) fitted with a guard column (Zorbax Eclipse XDB-C18, 2.1 mm ×5 mm, 1.8 µm; Agilent, 821725-903) and a 1290 Infinity II UHPLC (Agilent, G7120AR). The mobile phases used were 0.1% formic acid in water (mobile phase A) and 100% acetonitrile (mobile phase B). The analysis was performed at a flow rate of 0.7 ml min^−1^ and began with mobile phase B held at 4% for 1.35 min, followed by a linear gradient from 4% to 40% B over 1.25 min, a linear gradient from 40% to 100% B over 0.4 min, a linear gradient from 100% to 4% B over 0.7 min and then finally B held at 4% for 0.8 min. Acylation was confirmed by correctly identifying the exact masses of the 2′- and 3′-acyl-adenosine products corresponding to the substrate tested in the EICs recorded by LC–HRMS using a 6530 Q-TOF AJS-ESI instrument (Agilent, G6530BAR). The following parameters were used: fragmentor voltage = 175 V, gas temperature = 300 °C, gas flow = 12 l min^−1^, sheath gas temperature = 350 °C, sheath gas flow = 12 l min^−1^, nebulizer pressure = 35 psi, skimmer voltage =65 V, *V*_cap_ = 3,500 V and collection rate = 3 spectra s^−1^. The expected exact masses of the acyl-adenosine nucleosides (Supplementary Table 2) were calculated using ChemDraw 19.0 and extracted from the TICs (±100 ppm).

### Procedure for determining aminoacylation yields using intact tRNA MS

Enzymatic tRNA acylation reactions (25 µl) were performed as described in Procedure for RNAse A assays section. Sodium acetate (pH 5.2) was added to the acylation reactions to a final concentration of 300 mM in a volume of 200 µl. The reaction mixtures were then extracted once with a 1:1 (v/v) mixture of acidic phenol (pH 4.5) and chloroform and washed twice with chloroform. After extraction, the acylated tRNA was precipitated by adding ethanol to a final concentration of 71% and incubation at −80 °C for 30 min, followed by centrifugation at 21,300*g* for 30 min at 4 °C. After removing the supernatant, acylated tRNA was resuspended in water and kept on ice for analysis.

The tRNA samples from the enzymatic acylation reactions were analysed by LC–MS as described in Transcription and purification of tRNAs section. Because the unacylated tRNA peak in each TIC contained tRNA species that could not be enzymatically acylated (primarily tRNAs that lack the 3′-terminal adenosine^81^), simple integration of the acylated and non-acylated peaks in the absorbance at 260 nm (A_260_) chromatogram could not accurately quantify the acylation yield. To accurately quantify the acylation yield, we used the following procedure. For each sample, mass data were collected between *m/z* = 500 and 2,000. A subset of the mass data collected defined as the raw MS deconvolution range (Supplementary Figs. 3–35) was used to produce the deconvoluted mass spectra (Supplementary Figs. 3–35). The raw MS deconvolution range of each macromolecule species contained multiple peaks corresponding to different charge states of that macromolecule. Within the raw mass spectrum deconvolution range we identified the most abundant charge state peak in the raw mass spectrum of each tRNA species (unacylated, monoacylated and diacylated tRNA), which is identified as the major ion by an asterisk in Supplementary Figs. 3c–35c. To quantify the relative abundance of each species, the exact mass of the major ions (±0.3000 Da) was extracted from the TIC to produce the EICs (Supplementary Figs. 3–35). The EICs were integrated and the areas of the peaks that aligned with the correct peaks in the TIC (as determined from the deconvoluted mass spectrum) were used to quantify the yields (Supplementary Table 3). For the malonic acid substrates, the integrated peak areas of the EICs of both the malonic acid and decarboxylation products were added together to determine the overall acylation yield. Each sample was injected three times; the chromatograms and spectra in Supplementary Figs. 3–35 are representative, and the yields shown in Supplementary Table 3 are averages of the three injections. The expected masses of the oligonucleotide products were calculated using the AAT Bioquest RNA Molecular Weight Calculator, and the molecular masses of the small molecules added to them were calculated using ChemDraw 19.0. All the masses identified in the mass spectra are summarized in Supplementary Data 1.

### Malachite green assay to monitor adenylation

Enzymatic adenylation reactions were monitored using malachite green following a previous protocol with modifications^56^. Each adenylation reaction (60 µl) contained the following components: 200 mM HEPES-K (pH 7.5), 4 mM DTT, 10 mM MgCl_2_, 0.2 mM ATP, 0–10 mM substrate, 4 U m^−1^ *E. coli* inorganic pyrophosphatase (NEB) and 2.5 µM enzyme (*Ma*FRS1 or *Ma*FRSA). The adenylation reactions were incubated at 37 °C in a dry air incubator. Aliquots (10 µl) were withdrawn after 0, 5, 10, 20 and 30 min and quenched by addition to an equal volume of 20 mM EDTA (pH 8.0) on ice. Once all aliquots had been withdrawn, 80 µl malachite green solution (Echelon Biosciences) was added to each aliquot and the mixture incubated at room temperature for 30 min. After shaking for 30 s to remove bubbles, the absorbance at 620 nm was measured on a Synergy HTX plate reader (BioTek). The absorbance was then converted to phosphate concentration using a phosphate calibration curve (0–100 µM) and plotted against time to determine the turnover number.

### Structure determination

The following synthetic dsDNA sequence was cloned upstream of *Ma*FRSA (*Ma*PylRS N166A, V168A) into pET32a-*Ma*FRSA by Gibson assembly^75^ and used for subsequent crystallographic studies: GSS linker-6xHis-SSG linker-thrombin site-*Ma*FRSA (Supplementary Table 1). The sequence of the pET32a-6xHis-thrombin-*Ma*FRSA plasmid was confirmed by Sanger sequencing at Genewiz using the primers T7 F and T7 R (Supplementary Table 1). The procedure used to express and purify *Ma*FRSA for crystallography using pET32a-6xHis-thrombin-*Ma*FRSA was adapted from a protocol used to express and purify WT *Ma*PylRS (ref. ^60^). BL21-Gold (DE3)pLysS competent cells (Agilent) were transformed with pET32a-6xHis-thrombin-*Ma*FRSA and grown in TB media at 37 °C. Protein expression was induced at an OD_600_ of 1.2 with 1 mM IPTG. The temperature was lowered to 20 °C and growth was allowed to continue overnight. The cells were pelleted for 1 h at 4,300*g* and then resuspended in lysis buffer (50 mM potassium phosphate (pH 7.4), 25 mM imidazole, 500 mM sodium chloride, 5 mM β-mercaptoethanol and 1 cOmplete Mini EDTA-free protease inhibitor tablet). The cells were then lysed by homogenization (Avestin Emulsiflex C3). After centrifugation for 1 h at 10,000*g*, the clarified lysate was bound to TALON metal affinity resin (Takara Bio) for 1 h at 4 °C, washed with additional lysis buffer and eluted with elution buffer (50 mM potassium phosphate (pH 7.4), 500 mM imidazole, 500 mM sodium chloride and 5 mM β-mercaptoethanol). The eluate was dialysed overnight at 4 °C into cleavage buffer (40 mM potassium phosphate (pH 7.4), 100 mM NaCl and 1 mM DTT) and then incubated overnight at room temperature with thrombin protease on a solid agarose support (MilliporeSigma). Following cleavage, the protein was passed over additional TALON resin to remove the 6xHis tag and dialysed overnight at 4 °C into sizing buffer (30 mM potassium phosphate (pH 7.4), 200 mM NaCl and 1 mM DTT). The protein was concentrated and loaded onto a HiLoad 16/600 Superdex 200 pg column (Cytiva Life Sciences) equilibrated with sizing buffer on an ÄKTA Pure 25 fast-liquid chromatograph. Purified *Ma*FRSA was dialysed into storage buffer (10 mM Tris-HCl (pH 8.0), 150 mM NaCl, 10 mM MgCl_2_ and 10 mM β-mercaptoethanol), concentrated to 20 mg ml^−1^, aliquoted and flash-frozen for crystallography (Supplementary Fig. 2).

The initial crystallization screening conditions were adapted from Seki et al.^60^. Crystals were grown by hanging-drop vapour diffusion in 24-well plates. Then, 25 µl of 100 mM *m*-CF_3_-2-BMA (pH ≈ 7) was added to 1.5 ml microcentrifuge tubes and the water was removed by evaporation. The dried aliquots were then resuspended at a concentration of 100 mM with *Ma*FRSA in storage buffer at three concentrations (6.9, 12.3 and 19.2 mg ml^−1^) and 10 mM AMP-PNP lithium salt hydrate. The protein–substrate solution (1 µl) was mixed in a 1:1 ratio with the reservoir solution (1 µl) containing 10 mM Tris-HCl (pH 7.4) and 26% polyethylene glycol 3,350, and then incubated above 1 ml of reservoir solution at 18 °C. Crystals with an octahedral shape appeared within 1 week. The crystals were plunged into liquid nitrogen to freeze with no cryoprotectant.

Data were collected at the Advanced Light Source beamline 8.3.1 at 100 K using a wavelength of 1.11583 Å. Data collection and refinement statistics are presented in Supplementary Table 4. The diffraction data were indexed and integrated with XDS^82^, and then merged and scaled with Pointless^83^ and Aimless^84^. The crystals were formed in the space group *I*4 with unit cell dimensions 108.958, 108.958 and 112.26 Å. The structure was solved by molecular replacement with Phaser^85^ using a single chain of the WT apo structure of *Ma*PylRS (PDB code: 6JP2)^60^ as the search model. Two copies of *Ma*FRSA were present in the asymmetric unit. The model was improved with iterative cycles of manual model building in Coot (ref. ^86^) alternating with refinement in Phenix^87,88^ using data with a resolution of up to 1.8 Å. Structural analysis and figures were generated using Pymol (version 2.4.2)^89^.

### In vitro translation initiation

The *Ma*-tRNA^Pyl^-ACC dsDNA template was prepared as described in Transcription and purification of tRNAs section using the primers *Ma*-PylT-ACC F and *Ma*-PylT-ACC R (Supplementary Table 1). *Ma*-tRNA^Pyl^-ACC was also transcribed, purified and analysed as described previously. The enzymatic tRNA acylation reactions (150 µl) were performed as described in Procedure for RNAse A assays section with slight modifications. The enzyme concentration was increased to 12.5 µM (monomers **7**, **14** and **15**) or 25 µM (monomer **13**) and the incubation time was increased to 3 h at 37 °C. Sodium acetate (pH 5.2) was added to the acylation reactions to a final concentration of 300 mM in a volume of 200 µl. The reactions were then extracted once with a 1:1 (v/v) mixture of acidic phenol (pH 4.5) and chloroform and washed twice with chloroform. After extraction, the acylated tRNA was precipitated by adding ethanol to a final concentration of 71% and incubation at −80 °C for 30 min, followed by centrifugation at 21,300*g* for 30 min at 4 °C. The acylated tRNAs were resuspended in water to a concentration of 307 µM immediately before in vitro translation.

Templates for the expression of MGVDYKDDDDK were prepared by annealing and extending the oligonucleotides MGVflag-1 and MGVflag-2 using Q5 High-Fidelity 2x Master Mix (NEB; Supplementary Table 1). The annealing and extension procedure was performed using the following protocol on a thermocycler (BioRad C1000 Touch): 98 °C for 30 s, 10 cycles of 98 °C for 10 s, 55 °C for 30 s and 72 °C for 45 s, 10 cycles of 98 °C for 10 s, 67 °C for 30 s and 72 °C for 45 s, and finally 72 °C for 300 s. Following the extension, the reaction mixture was supplemented with sodium acetate (pH 5.2) to a final concentration of 300 mM, extracted once with a 1:1 (v/v) mixture of basic phenol (pH 8.0) and chloroform, and washed twice with chloroform. The dsDNA product was precipitated by the addition of ethanol to a final concentration of 71% and incubation at −80 °C for 30 min, followed by centrifugation at 21,300*g* for 30 min at 4 °C. The dsDNA pellets were washed once with 70% (v/v) ethanol, resuspended in 10 mM Tris-HCl (pH 8.0) to a concentration of 500 ng µl^−1^ and stored at −20 °C until use in translation.

In vitro transcription/translation by codon skipping of the short FLAG tag-containing peptides X-Val-Asp-Tyr-Lys-Asp-Asp-Asp-Asp-Lys (XV-Flag, where X = **7**, **13**, **14** or **15**) was carried out using the PURExpress Δ (aa, tRNA) Kit (NEB, E6840S) according to a previous protocol with slight modifications^17^. The XV-Flag peptides were produced from the following reaction mixtures (12.5 µl): solution A (∆tRNA and ∆aa; 2.5 µl), amino acid stock mix (33 mM l-valine, 33 mM l-aspartic acid, 33 mM l-tyrosine and 33 mM l-lysine; 1.25 µl), tRNA solution (1.25 µl), solution B (3.75 µl), 250 ng dsDNA MGVDYKDDDDK template (0.5 µl) and *Ma*-tRNA^Pyl^-ACC acylated with **7**, **13**, **14** or **15** (3.25 µl). The reactions were incubated in a thermocycler (BioRad C1000 Touch) at 37 °C for 2 h and quenched on ice.

The translated peptides were purified from the in vitro translation reaction mixtures by enrichment using anti-FLAG M2 magnetic beads (MilliporeSigma) according to the manufacturer’s protocol with slight modifications. For each peptide, 10 µl of a 50% (v/v) suspension of magnetic beads was used. The supernatant was pipetted from the beads on a magnetic manifold. The beads were then washed twice by incubating with 100 µl TBS buffer (150 mM NaCl and 50 mM Tris-HCl (pH 7.6)) for 10 min at room temperature and then removing the supernatant each time using a magnetic manifold. The in vitro translation reaction mixtures were added to the beads and incubated at room temperature for 30 min with periodic agitation. The beads were washed again three times with 100 µl TBS as described above. The peptides were eluted upon incubation with 12.5 µl of 0.1 M glycine·HCl (pH 2.8) for 10 min. The supernatant was transferred to vials and kept on ice for analysis.

The purified peptides were analysed according to a previously reported protocol^17^. The supernatant was analysed on an ZORBAX Eclipse XDB-C18 column (1.8 μm, 2.1 mm × 50 mm, room temperature; Agilent) using a 1290 Infinity II UHPLC (Agilent, G7120AR). The following protocol was used for separation: an initial hold at 95% solvent A (0.1% formic acid in water) and 5% solvent B (acetonitrile) for 0.5 min, followed by a linear gradient from 5% to 50% solvent B for 4.5 min at a flow rate of 0.7 ml min^−1^. The peptides were identified by LC–HRMS using a 6530 Q-TOF AJS-ESI instrument (Agilent, G6230BAR). The following parameters were used: fragmentor voltage = 175 V, gas temperature = 300 °C, gas flow rate = 12 l min^−1^, sheath gas temperature = 350 °C, sheath gas flow rate = 11 l min^−1^, nebulizer pressure = 35 psi, skimmer voltage = 5 V, *V*_cap_ = 3,500 V and collection rate = 3 spectra s^−1^. The expected exact masses of the major charge state of each peptide were calculated using ChemDraw 19.0 and extracted from the TICs (±100 ppm).

### Plasmids used for in vivo studies

The plasmids used to express WT sfGFP (pET22b-T5/lac-sfGFP) and 151TAG-sfGFP (pET22b-T5/lac-sfGFP-151TAG) in *E. coli* have been described previously^90^. pET22b-T5/lac-sfGFP-200TAG was constructed from pET22b-T5/lac-sfGFP using a Q5 Site-Directed Mutagenesis Kit (NEB) with primers CS43 and CS44 (Supplementary Table 1). The synthetase/tRNA plasmid for WT *Ma*PylRS (pMega-*Ma*PylRS) was constructed by inserting a synthetic dsDNA fragment (pMega-*Ma*PylRS) (Supplementary Table 1) into the NotI-XhoI cut sites of a pUltra vector^65^ by Gibson assembly^75^ using NEBuilder HiFi DNA Assembly Master Mix (NEB). pMega-*Ma*FRSA was constructed by inserting a synthetic dsDNA fragment (made by annealing primers RF48 and RF49) after inverse PCR of pMega-*Ma*PylRS with primers RF61 and RF62 (Supplementary Table 1) using Gibson assembly^75^. The sequences of the plasmids spanning the inserted regions were confirmed via Sanger sequencing at the University of California Berkeley DNA Sequencing Facility using primers T7 F and T7 R (Supplementary Table 1), and the complete sequence of each plasmid was confirmed by full-plasmid sequencing at Primordium Labs.

### Plate reader analysis of sfGFP expression

*E. coli* DH10B chemically competent cells were transformed with pET22b-T5/lac-sfGFP-200TAG and either pMega-*Ma*PylRS or pMega-*Ma*FRSA. Colonies were picked and grown overnight in LB with the appropriate antibiotics. The following day, the OD_600_ of the overnight culture was measured, and all cultures were diluted with LB to an OD_600_ of 0.10 to generate a seed culture. A monomer cocktail was prepared in LB supplemented with 2 mM IPTG, 2 mM monomer **1**, **2**, **20** or **21**, and the appropriate antibiotics at two times the final concentration (200 µg ml^−1^ carbenicillin and 100 µg ml^−1^ spectinomycin). In a 96-well plate (Corning 3904), 100 µl of the seed culture was combined with 100 µl of each monomer cocktail to bring the starting OD_600_ to 0.05 and half the concentration of the monomer cocktail. The 96-well plate was sealed with a Breathe-Easy sealing membrane (Diversified Biotech) and loaded into a Synergy HTX plate reader (BioTek). The plate was incubated at 37 °C for 24 h with continuous shaking. Two readings were made at 10 min intervals, that is, the absorbance at 600 nm, to measure cell density, and sfGFP fluorescence with excitation at 485 nm and emission at 528 nm.

### Expression and purification of sfGFP variants

The plasmids used to express WT sfGFP and sfGFP-200TAG were co-transformed with pMega-*Ma*PylRS or pMega-*Ma*FRSA into DH10B or DH10B Δ*aspC* Δ*tyrB* chemically competent cells and plated onto LB agar plates supplemented with 100 µg ml^−1^ carbenicillin and 100 µg ml^−1^ spectinomycin. Colonies were picked the following day and used to inoculate 10 ml LB supplemented with 100 µg ml^−1^ carbenicillin and 100 µg ml^−1^ spectinomycin. The cultures were incubated overnight at 37 °C with shaking at 200 r.p.m. The following day, 1 ml of each culture was used to inoculate 100 ml TB or defined media (adapted from a published protocol^55^ with glutamate excluded and 19 other amino acids at 200 µg ml^−1^) supplemented with 100 µg ml^−1^ carbenicillin and 100 µg ml^−1^ spectinomycin in 250-ml baffled Erlenmeyer flasks. The cultures were incubated at 37 °C with shaking at 200 r.p.m. for ~4 h until they reached an OD_600_ of 1.0–1.2. At this point, IPTG was added to a final concentration of 1 mM and incubation was continued overnight at 37 °C with shaking at 200 r.p.m. The cells were collected by centrifugation at 4,303*g* for 20 min at 4 °C.

sfGFP variants were purified according to a previously published protocol^71^. The following buffers were used for protein purification: lysis/wash buffer: 50 mM sodium phosphate (pH 8), 300 mM NaCl and 20 mM imidazole; elution buffer: 50 mM sodium phosphate (pH 8) and 250 mM imidazole; storage buffer: 50 mM sodium phosphate (pH 7), 250 mM NaCl and 1 mM DTT. One cOmplete Mini EDTA-free protease inhibitor tablet was added to the wash and elution buffers immediately before use. To isolate protein, cell pellets were resuspended in 10 ml wash buffer. The resultant cell paste was lysed at 4 °C by homogenization (Avestin Emulsiflex C3) for 5 min at 15,000–20,000 psi. The lysate was centrifuged at 4,303*g* for 15 min at 4 °C to separate the soluble and insoluble fractions. The soluble lysate was incubated at 4 °C with 1 ml TALON resin (washed with water and equilibrated with wash buffer) for 1 h. The lysate–resin mixture was centrifuged at 4,303*g* for 5 min to pellet. The supernatant was removed and the protein-bound TALON resin was then washed three times with 5 ml lysis/wash buffer, centrifuging between washes to pellet. The protein was eluted from TALON resin by rinsing the resin five times with 1 ml elution buffer. The elution fractions were pooled and dialysed overnight at 4 °C into storage buffer using 12,000–14,000 MWCO dialysis tubing. The protein concentration was measured using the Pierce 660 nm assay^91^. Protein samples were concentrated as needed with a 10 kDa MWCO Amicon Ultra-15 centrifugal filter unit (4,303*g*, 4 °C) to reach a concentration of ≥0.22 mg ml^−1^ (Supplementary Fig. 37). The protein was stored at 4 °C for later analysis. Yields were between 24 and 324 mg l^−^^1^ when expressed in TB, and between 3.6 and 3.7 mg l^−^^1^ when expressed in the defined media described above. Proteins were analysed by LC–MS as described above.

### Protease digestion and fragment identification by MS

Each isolated sfGFP sample (~10–25 µg) was denatured with 6 M guanidine in 0.15 M Tris buffer (pH 7.5), followed by disulfide reduction with 8 mM DTT at 37 °C for 30 min. The reduced sfGFP was alkylated in the presence of 14 mM iodoacetamide at 25 °C for 25 min, followed by quenching using 6 mM DTT. The reduced and alkylated protein was exchanged into ~40 µl of 0.1 M Tris buffer (pH 7.5) using a Microcon 10 kDa membrane, and then 2.5 µg endoproteinase GluC (in a 0.25 µg µl^−1^ solution) was added directly to the membrane to achieve an enzyme/substrate ratio of at least 1:10. After 3 h at 37 °C, the digestion was quenched with an equal volume of 0.25 M acetate buffer (pH 4.8) containing 6 M guanidine. The peptide fragments were collected by spinning down through the membrane and subjected to LC–MS/MS analysis.

LC–MS/MS analysis was performed on an Agilent 1290-II HPLC device directly connected to a Thermo Fisher Q Exactive HF high-resolution mass spectrometer. Peptides were separated on a Waters HSS T3 reversed-phase column (2.1 mm × 150 mm) at 50 °C with a 70 min acetonitrile gradient (0.5–35%) in water containing 0.1% formic acid in the mobile phase at a total flow rate of 0.25 ml min^−1^. The MS data were collected at a resolution of 120,000, followed by data-dependent higher-energy collision dissociation MS/MS at a normalized collision energy of 25%.

Proteolytic peptides were identified and quantified using MassAnalyzer, a program^92^ developed in house (available in Biopharma Finder, Thermo Fisher). The program performs feature extraction, peptide identification, retention time alignment^93^ and peak integration in an automated fashion.

### Reporting summary

Further information on research design is available in the Nature Portfolio Reporting Summary linked to this article.

## Online content

Any methods, additional references, Nature Portfolio reporting summaries, source data, extended data, supplementary information, acknowledgements, peer review information; details of author contributions and competing interests; and statements of data and code availability are available at 10.1038/s41557-023-01224-y.

## Supplementary information

Supplementary Information Materials, Synthesis notes, Supplementary Figs. 1–43, Tables 1–5 and References.

Reporting Summary

Supplementary Data 1 All masses identified in intact tRNA mass spectra are listed here. N-1 and N-2 refer to the tRNA missing the final nucleotides 1 and 2 at the 3′ end, respectively. P and PPP indicate that the 5′ end of the tRNA has a mono- or triphosphate, respectively. N+G and N+GG refer to tRNA products with non-templated addition of guanosine residues identified in the mass spectrum. Note that for some enzyme/substrate pairs there is evidence that the N+G products are acylated by the synthetase, suggesting that the untemplated guanosine addition does not exclude these tRNA species from activity with the synthetase.

## Source data

Source Data Fig. 1 Numerical MS data.

Source Data Fig. 2 Numerical MS data.

Source Data Fig. 3 Numerical MS data.

Source Data Fig. 4 Numerical MS data.

Source Data Fig. 6 Numerical MS data.

Source Data Extended Data Fig. 1 Numerical MS data.

Source Data Extended Data Fig. 2 Numerical MS data.

Source Data Extended Data Fig. 6 Numerical fluorescence emission data.

## Data Availability

The structural model of *Ma*FRSA is available in the Protein Data Bank with PDB code 7U0R. Data for Supplementary Figs. 1–43 are available at Zenodo (10.5281/zenodo.7754396). Source data are provided with this paper.
