# Supplementary material for: Expanding the substrate scope of pyrrolysyl-transfer RNA synthetase enzymes to include non-α-amino acids in vitro and in vivo
Source: Nat Chem. 2023 Jun 1;15(7):960–71. doi: 10.1038/s41557-023-01224-y (PMC10322718; doi:10.1038/s41557-023-01224-y)
Supplement: Supplementary file 1 — Materials, Synthesis notes, Supplementary Figs. 1–43, Tables 1–5 and References. [file 41557_2023_1224_MOESM1_ESM.pdf]

nature chemistry

Article

https://doi.org/10.1038/s41557-023-01224-y

Expanding the substrate scope of pyrrolysyl-transfer RNA synthetase enzymes to include non-α-amino acids in vitro and in vivo

In the format provided by the authors and unedited

Table of Contents

**Materials**	1
**Synthesis Notes**	3
**Supplementary Figures**	9
**Supplementary Tables**	54
**References**	67

Materials

Materials were sourced from the following suppliers: **Agilent Technologies** (Santa Clara, CA): BL21-Gold (DE3) Competent Cells; **Accela Chembio** (San Diego, CA): (S)-6-((tert-butoxycarbonyl)amino)-2-hydroxyhexanoic acid; **Alfa Aesar** (Ward Hill, MA): N(ε)-Boc-L-lysine (L-BocK), N(ε)-Boc-D-lysine (D-BocK), 3-Methylbenzyl bromide, 3-(Trifluoromethyl)benzyl bromide, 3-Bromobenzyl bromide, L-lysine monohydrochloride; **AmericanBio** (Canton, MA): carbenicillin, glycerol, isopropyl β-D-1-thiogalactopyranoside (IPTG), HEPES, magnesium chloride (1 M solution), spectinomycin sulfate, sodium acetate buffer (3 M, pH 5.2), EDTA-Na (0.5 M solution, pH 8.0); **BACHEM** (Torrance, CA): N-methyl-L-phenylalanine, N-formyl-L-phenylalanine; **BioRad** (Hercules, CA): Any kD™ Mini-PROTEAN® TGX™ Precast Protein Gels (product 4569033), 10% Mini-PROTEAN® TBE-Urea Gel (product 4566036), Micro Bio-Spin™ P-30 Gel Columns, Tris Buffer RNase-free (product 7326250), Precision Plus Protein™ Dual Color Standards (product 1610374); **BioWorld** (Dublin, OH): Luria-Bertani broth (LB), Terrific broth (TB); **Cayman Chemical** (Ann Arbor, MI): α-mercapto-benzenepropanoic acid; **Corning** (Kennebunk, ME): 96-well plate #3904; **Cytiva Life Sciences** (Marlborough, MA): Superdex® 75 Increase 10/300 GL column, HiLoad® 16/600 Superdex® 200 pg column; **Decon Labs** (King of Prussia, PA): 200 proof ethanol; **Diversified Biotech** (Dedham, MA): Breathe-Easy® membranes; **Echelon Biosciences** (Salt Lake City, UT): malachite green solution; **Enamine** (Kyiv, Ukraine): 2-hydroxy-3-(3-(trifluoromethyl)phenyl)propanoic acid; **Fisher Scientific** (Pittsburgh, PA): agar, sodium hydroxide, potassium hydroxide, sodium chloride, potassium chloride, calcium chloride, dithiothreitol (DTT), 50% polyethylene glycol 3350 solution, acetonitrile Optima™ LC/MS Grade, Tris base, ethylenediaminetetraacetic acid (free acid), Pierce™ 660 nm Protein Assay

Reagent; **Frontier Scientific** (Logan, UT): L-phenylalanine, L-aspartic acid, L-valine, L-tyrosine; **Honeywell** (Charlotte, NC): 1,1,1,3,3,3-Hexafluoro-2-propanol, LC-MS Grade (HFIP); **Integrated DNA Technologies** (Coralville, IA): RF31, RF32, RF33, *Ma*-PylT-F, *Ma*-PylT-R; **Invitrogen** (Waltham, MA): SYBR™ Safe DNA Gel Stain; **J.T.Baker - Avantor** (Radnor, PA): sodium phosphate, chloroform, boric acid, hydrochloric acid, dimethylsulfoxide (DMSO); **MilliporeSigma** (Burlington, MA): β-mercaptoethanol (BME), imidazole, cesium chloride, adenosine 5′-(β,γ-imido)triphosphate lithium salt hydrate (AMP-PNP), ribonuclease A from bovine pancrease (RNAse A), acidic phenol (Phenol Saturated Citrate Buffered pH 4.5), ethanol, spermidine, guanosine monophosphate (GMP), bovine serum albumin (BSA), polyethylene glycol 8000, 6-(Boc-amino)hexanoic acid (BocAhx), (*S*)-(−)-3-phenyllactic acid (3-PLA), 2-benzylmalonic acid (2-BMA), 4-(Boc-amino)butyl bromide, diethyl malonate, tetrahydrofuran anhydrous, sodium hydride 60 % dispersion in mineral oil, sodium sulfate anhydrous, diethylether, Thrombin CleanCleave™ Kit, 10 kDa MWCO Amicon® Ultra-15 Centrifugal Filter Unit, Anti-FLAG® M2 Magnetic Beads, basic phenol (Phenol solution equilibrated with 10 mM Tris HCl, pH 8.0, 1 mM EDTA); **MP Biomedicals** (Irvine, CA): D-phenylalanine, glycine hydrochloride; **New England BioLabs** (Ipswich, MA): NdeI restriction enzyme, NEBuilder® HiFi DNA Assembly Master Mix, OneTaq® Quick-Load® 2X Master Mix, nucleotide triphosphate solutions, Low Range ssRNA Ladder, PURExpress® Δ (aa, tRNA) Kit, Q5® High-Fidelity 2X Master Mix, Q5® Site-Directed Mutagenesis Kit; **PepTech** (Bedford, MA): 3-Trifluoromethyl-L-phenylalanine; **Promega** (Madison, WI): RQ1 RNase-Free DNase **Qiagen** (Germantown, MD): Ni-NTA Agarose resin; **Ricca Chemical Company** (Arlington, TX): formic acid LCMS grade, triethylamine (TEA) LCMS grade; **Roche** (Basel, Switzerland): cOmplete™ Mini EDTA-free Protease Inhibitor Cocktail; **Takara Bio** (San Jose,

CA): TALON® Metal Affinity Resin; **Teledyne ISCO** (Lincoln, NE): 65 g RediSep® Disposable Sample Load Cartridge; **Tokyo Chemical Industry** (Portland, OR): 3-phenylpropanoic acid (3-PLA).

Synthesis notes

Synthesis of *meta*-substituted 2-benzylmalonates 17-19 and malonate 16.

General. Alkylation reactions to synthesize **22**-**25** as well as hydrolysis reactions to synthesize **16**-**19** were based on published methods[1,2]. All reagents and solvents were used as received from commercial suppliers, unless indicated otherwise. Alkylation reactions were carried out with exclusion of air and moisture. Room temperature is considered 20–23 °C. Stirring was achieved with Teflon-coated magnetic stir bars. TLC was performed on glass-backed silica gel plates (median pore size 60 Å) and visualized using UV light at 254 nm or staining with iodine. Column chromatography was performed on an Isco Teledyne Combiflash Nextgen 300+ instrument using pre-packed Redi-sep Gold silica gel cartridges (particle diameter 20-40 μM, pore diameter 60 Å). The eluents are given in brackets. Mass spectrometry was performed on an LTQ FT-ICR mass spectrometer equipped with an electrospray ionization source (Finnigan LTQ FT, Thermo Fisher Scientific, Waltham, MA) operated in either positive or negative ion mode. ^{1}H NMR spectra NMR data were acquired at 298 K using a 500 MHz Bruker Avance Neo NMR spectrometer that was equipped with a 5 mm iProbe or a 400 MHz Bruker Avance I spectrometer equipped with a 5 mm BBO Smart Probe. The experiments were conducted using the default Bruker NMR parameters and data was time-averaged until a sufficient level of sensitivity was achieved. ^{1}H NMR data was calibrated by using the residual peak of the solvent as the internal standard ($CDCl_3$: δ_H = 7.26 ppm; CD_3OD: δ_H = 3.31 ppm). All coupling constants are recorded in

Hz. NMR spectra were processed with MestReNova v14.1.2-25024 software using the baseline and phasing correction features. Multiplicities and coupling constants were calculated using the multiplet analysis feature with manual intervention as necessary.

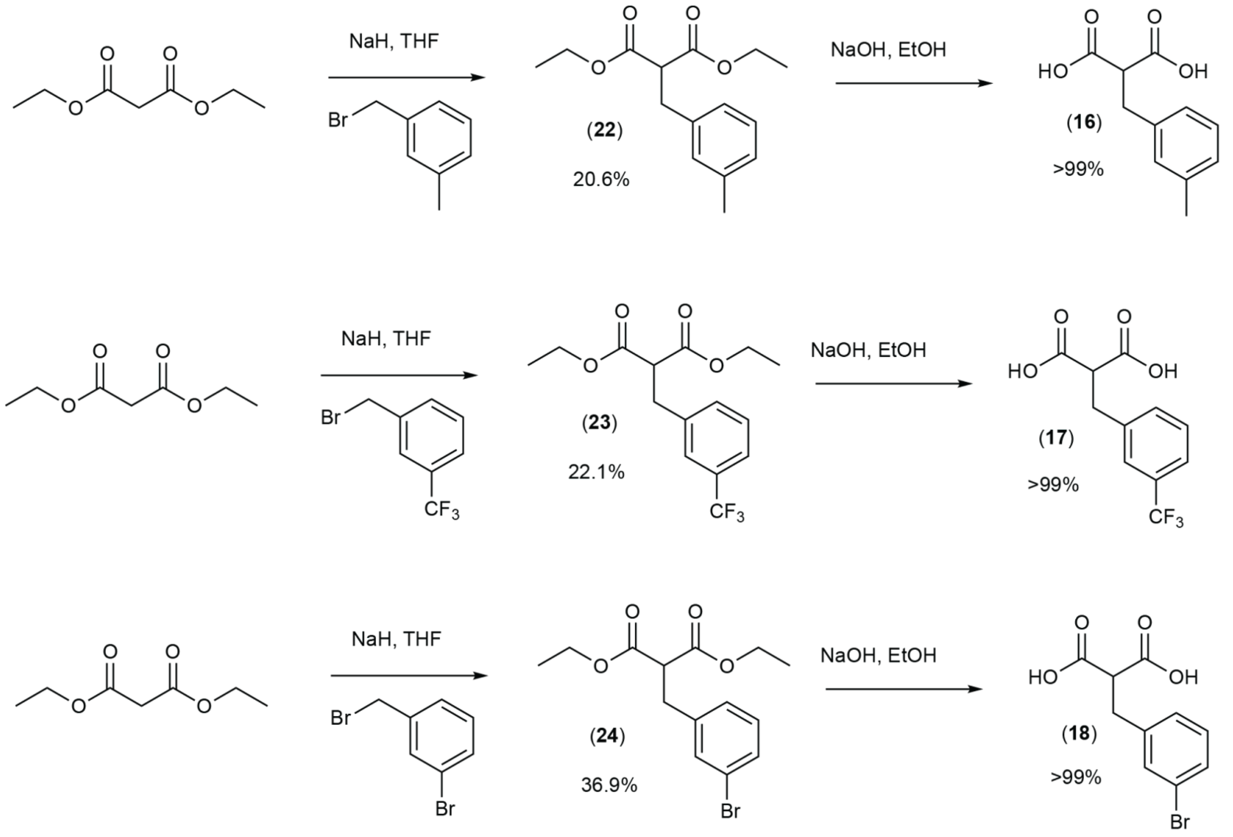

Diethyl 2-(3-methylbenzyl)malonate (22) Diethyl malonate (500.57 mg, 3.125 mmol, 1.05 equiv.) was added dropwise to a suspension of 60% NaH on mineral oil (125 mg, 3.125 mmol, 1.05 equiv.) in 6 mL dry THF at 0 °C. After 20 min, 3-methylbenzyl bromide (550.86 mg, 2.97 mmol, 1 equiv.) was added in one portion and the reaction mixture was refluxed overnight. The next day, the reaction was cooled and quenched by the addition of H_2O. Et_2O was added and the aqueous layer was extracted three times with Et_2O. The combined organic layers were dried over Na_2SO_4 then evaporated to dryness under reduced pressure. The crude product was purified by flash chromatography on SiO_2 [eluent: EtOAc/hexane (5% then 10% then 15% then 20%)] to

obtain pure diethyl 2-(3-methylbenzyl)malonate **22** as a clear liquid. Yield 20.6%. ^{1}H NMR (500 MHz, $CDCl_3$) δ 7.19 (t, J = 7.8 Hz, 1H), 7.07 – 7.00 (m, 3H), 4.19 (qd, J = 7.1, 2.8 Hz, 4H), 3.65 (t, J = 7.8 Hz, 1H), 3.20 (d, J = 7.8 Hz, 2H), 2.34 (s, 3H), 1.24 (t, J = 7.1 Hz, 6H). HR-EI-MS [M+H]+: calculated for C15H21O4+, *m/z* 265.1434, found *m/z* 265.1395.

2-(3-methylbenzyl)malonic acid (17) Diethyl 2-(3-methylbenzyl)malonate **22** (100 mg, 0.362 mmol) was dissolved in 1 mL of ethanol then added dropwise to 375 µL of 6.67 M NaOH. The mixture was stirred for 5 h at 60 °C. The solution was then cooled to 0 °C, carefully acidified to pH 1 with 1 N HCl, and extracted with 5 portions of Et_2O. The combined extracts were washed with a saturated aqueous solution of NaCl, dried over Na_2SO_4 and evaporated to dryness. 2-(3-methylbenzyl)malonic acid was dissolved in 1:1 H_2O:MeCN and lyophilized to give a white solid. Yield >99%. ^{1}H NMR (500 MHz, MeOD) δ 7.04 (t, J = 7.6 Hz, 1H), 6.95 (s, 1H), 6.94 – 6.88 (m, 2H), 3.49 (t, J = 7.8 Hz, 1H), 3.01 (d, J = 7.7 Hz, 2H), 2.20 (s, 3H). HR-ESI-MS [M-H]-: calculated for C11H11O4, *m/z* 207.0663, found *m/z* 207.0661.

Diethyl 2-(3-(trifluoromethyl)benzyl)malonate (23) Diethyl malonate (500.57 mg, 3.125 mmol, 1.05 equiv.) was added dropwise to a suspension of 60% NaH on mineral oil (125 mg, 3.125 mmol, 1.05 equiv.) in 6 mL dry THF at 0°C. After 20 min, 3-(trifluoromethyl)benzyl bromide (711.377 mg, 2.97 mmol, 1 equiv.) was added in one portion and the reaction mixture was refluxed overnight. The next day, the reaction was cooled and quenched by the addition of H_2O. Et_2O was added and the aqueous layer was extracted three times with Et_2O. The combined organic layers were then dried over Na_2SO_4 then evaporated to dryness under reduced pressure. The crude product was purified by flash chromatography on SiO_2 [eluent: EtOAc/hexane (5%

then 10% then 15% then 20%)] to obtain pure diethyl 2-(3-(trifluoromethyl)benzyl)malonate as a clear liquid. Yield 22.1%. ^{1}H NMR (400 MHz, CDCl3) δ 7.56 – 7.49 (m, 2H), 7.45 (p, J = 2.1 Hz, 2H), 4.21 (qd, J = 7.2, 1.7 Hz, 4H), 3.68 (t, J = 7.8 Hz, 1H), 3.31 (d, J = 7.9 Hz, 2H), 1.25 (t, J = 7.1 Hz, 6H). HR-EI-MS [M+H]+: calculated for C15H18F3O4+, *m/z* 319.1152, found *m/z* 319.1112.

2-(3-(trifluoromethyl)benzyl)malonic acid (18) Diethyl 2-(3-(trifluoromethyl)benzyl)malonate (100 mg, 0.314 mmol) was dissolved in 1 mL of ethanol then added dropwise to 375 µL of 6.67 M NaOH. The mixture was stirred for 5 h at 60 °C. The solution was then cooled to 0°C, carefully acidified to pH 1 with 1 N HCl, and extracted with 5 portions of Et_2O. The combined extracts were washed with a saturated solution of NaCl, dried over Na_2SO_4, and evaporated to dryness. 2-(3-(trifluoromethyl)benzyl)malonic acid was dissolved in 1:1 H_2O:MeCN and lyophilized to give a white solid. Yield >99%. ^{1}H NMR (500 MHz, MeOD) δ 7.60 – 7.46 (m, 4H), 3.69 (t, J = 7.8 Hz, 1H), 3.26 (d, J = 7.7 Hz, 2H). HR-ESI-MS [M-H]-: calculated for C11H8F3O4, *m/z* 261.0380, found *m/z* 261.0377.

Diethyl 2-(3-bromobenzyl)malonate (24) Diethyl malonate (500.57 mg, 3.125 mmol, 1.05 equiv.) was added dropwise to a suspension of 60% NaH on mineral oil (125 mg, 3.125 mmol, 1.05 equiv.) in 6 mL dry THF at 0 °C. After 20 min, 3-bromobenzyl bromide (743.91 mg, 2.97 mmol, 1 equiv.) was added in one portion and the reaction mixture was refluxed overnight. The next day, the reaction was cooled and quenched by the addition of H_2O. Et_2O was added and the aqueous layer was extracted three times with Et_2O. The combined organic layers were then dried over Na_2SO_4 then evaporated to dryness under reduced pressure. The crude product was purified

by flash chromatography on SiO_2 [eluent: EtOAc/hexane (5% then 10% then 15% then 20%)] to obtain pure diethyl 2-(3-bromobenzyl)malonate as a clear liquid. Yield 22.1%. ^{1}H NMR (400 MHz, CDCl3) δ 7.39 – 7.32 (m, 2H), 7.19 – 7.10 (m, 2H), 4.17 (qd, J = 7.1, 1.3 Hz, 4H), 3.61 (t, J = 7.8 Hz, 1H), 3.18 (d, J = 7.9 Hz, 2H), 1.22 (t, J = 7.1 Hz, 6H). HR-EI-MS [M+H]+: calculated for C14H18BrO4, *m/z* 329.0383, found *m/z* 329.0343.

2-(3-bromobenzyl)malonic acid (19) Diethyl 2-(3-bromobenzyl)malonate **24** (100 mg, 0.305 mmol) was dissolved in 1 mL of ethanol then added dropwise to 375 µL of 6.67 M NaOH. The mixture was stirred for 5 h at 60 °C. The solution was then cooled to 0 °C, carefully acidified to pH 1 with 1 N HCl, and extracted with 5 portions of Et_2O. The combined extracts were washed with a saturated solution of NaCl, dried over Na_2SO_4, and evaporated to dryness.
2-(3-bromobenzyl)malonic acid was dissolved in 1:1 H_2O:MeCN and lyophilized to give a white solid. Yield >99%. ^{1}H NMR (500 MHz, MeOD) δ 7.33 (t, J = 1.9 Hz, 1H), 7.26 (dt, J = 7.7, 1.7 Hz, 1H), 7.16 – 7.06 (m, 2H), 3.52 (t, J = 7.8 Hz, 1H), 3.04 (d, J = 7.8 Hz, 2H). HR-ESI-MS [M-H]-: calculated for C10H8BrO4, *m/z* 270.9611, found *m/z* 270.9609.

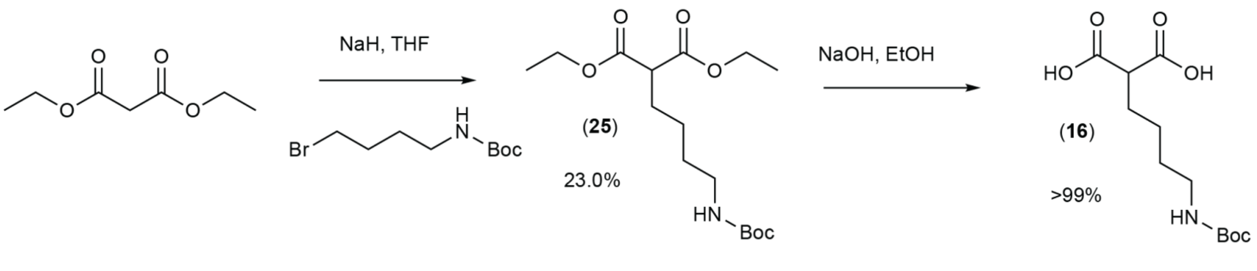

Diethyl 2-(4-((tert-butoxycarbonyl)amino)butyl)malonate (25) Diethyl malonate (166.86 mg, 1.04 mmol, 1.05 equiv.) was added dropwise to a suspension of 60% NaH on mineral oil (41.67 mg, 1.04 mmol, 1.05 equiv.) in 6 mL dry THF at 0 °C. After 20 min, 4-(Boc-amino)butyl

bromide (250.17 mg, 0.99 mmol, 1 equiv.) was added in one portion and the reaction mixture was refluxed overnight. The next day, the reaction was cooled and quenched by the addition of H_2O. Et_2O was added and the aqueous layer was extracted three times with Et_2O. The combined organic layers were then dried over Na_2SO_4 then evaporated to dryness under reduced pressure. The crude product was purified by flash chromatography on SiO_2 [eluent: EtOAc/hexane (5% then 10% then 15% then 20%)] to obtain pure diethyl 2-(4-((tert-butoxycarbonyl)amino)butyl)malonate as a clear liquid. Yield 22.1%. ^{1}H NMR (500 MHz, CDCl3) δ 4.44 (s, 1H), 4.13 (qd, J = 7.1, 2.0 Hz, 4H), 3.24 (t, J = 7.5 Hz, 1H), 3.04 (q, J = 6.7 Hz, 2H), 1.87 – 1.79 (m, 2H), 1.44 (p, J = 7.3 Hz, 2H), 1.37 (s, 9H), 1.28 (tt, J = 10.5, 6.3 Hz, 2H), 1.20 (t, J = 7.1 Hz, 6H).

2-(4-((tert-butoxycarbonyl)amino)butyl)malonic acid (16) Diethyl 2-(4-((tert-butoxycarbonyl)amino)butyl)malonate (72 mg, 0.22 mmol) was dissolved in 1 mL of ethanol then added dropwise to 375 µL of 6.67 M NaOH. The mixture was stirred for 5 h at 60 °C. The solution was then cooled to 0 °C, carefully acidified to pH 1 with 1 N HCl, and extracted with 5 portions of Et_2O. The combined extracts were washed with a saturated solution of NaCl, dried over Na_2SO_4, and evaporated to dryness. 2-(4-((tert-butoxycarbonyl)amino)butyl)malonic acid was dissolved in 1:1 H_2O:MeCN and lyophilized to give a clear serum. Yield >99%. ^{1}H NMR (500 MHz, CDCl3) δ 6.29 (s, 1H), 4.57 (s, 1H), 3.35 (t, J = 7.0 Hz, 1H), 3.03 (t, J = 6.7 Hz, 2H), 1.92 – 1.85 (m, 2H), 1.43 (p, J = 6.7 Hz, 2H), 1.36 (s, 11H). HR-ESI-MS [M-H]-: calculated for C12H20O6N1 *m/z* 274.1296, found *m/z* 274.1295.

Supplementary Figures

A

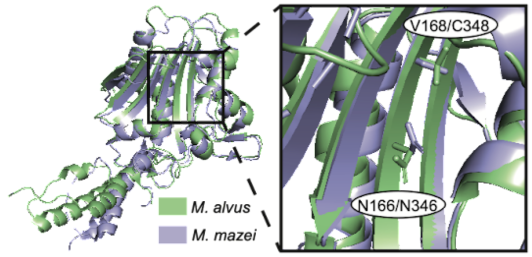

B

```
MaPylRS    1 -MTVKYTDAQIQRLREYGNGTYE-----QKVFEDLASRDAAFSKEMSVASTDNEKKIKGM  54
              .....|.:|..||....|...|      .|.|.:|.|.          ..:..:|.::.:
MmPylRS  185 ASAPALTKSQTDRLEVLLNPKDEISLNSGKPFRELESE----------LLSRRKKDLQQI 234

MaPylRS   55 IANPSRHGLTQLMNDIADALVAEGFIEVRTPIFISKDALARMTITEDKPLFKQVFWIDEK 114
             .|....:.|.:|..:|....|..||:|:::||.|..:.:.||.|..|..|.||:|.:|:.
MmPylRS  235 YAEERENYLGKLEREITRFFVDRGFLEIKSPILIPLEYIERMGIDNDTELSKQIFRVDKN 294

MaPylRS  115 RALRPMLAPNLYSVMRDLRDHTDGPVKIFEMGSCFRKESHSGMHLEEFTMLNLVDMGPRG 174
             ..||||||||||:.:|.|......|:||||:|.|:||||....|||||||||...|| .|
MmPylRS  295 FCLRPMLAPNLYNYLRKLDRALPDPIKIFEIGPCYRKESDGKEHLEEFTMLNFCQMG-SG 353

MaPylRS  175 DATEVLKNYISVVMKAAGLPDYDLVQEESDVYKETIDVEINGQEVCSAAVGPHYLDAAHD 234
             ...|.|::.|:..:...|: |:.:|.:...||.:|:||.....|:.||.|||..||....
MmPylRS  354 CTRENLESIITDFLNHLGI-DFKIVGDSCMVYGDTLDVMHGDLELSSAVVGPIPLDREWG 412

MaPylRS  235 VHEPWSGAGFGLERLLTIREKYSTVKKGGASISYLNGAKIN*  275
             :.:||.||||||||||.::..:..:|:...|.||.||...|.
MmPylRS  413 IDKPWIGAGFGLERLLKVKHDFKNIKRAARSESYYNGISTNL  454

| = identical
: = similar
. = unrelated
```

C

```
MaPylRS  MTVKYTDAQI QRLREYGNGT YEQKVFEDLA SRDAAFSKEM SVASTDNEKK IKGMIANPSR
FRS1     MTVKYTDAQI QRLREYGNGT YEQKVFEDLA SRDAAFSKEM SVASTDNEKK IKGMIANPSR
FRS2     MTVKYTDAQI QRLREYGNGT YEQKVFEDLA SRDAAFSKEM SVASTDNEKK IKGMIANPSR
FRSA     MTVKYTDAQI QRLREYGNGT YEQKVFEDLA SRDAAFSKEM SVASTDNEKK IKGMIANPSR

MaPylRS  HGLTQLMNDI ADALVAEGFI EVRTPIFISK DALARMTITE DKPLFKQVFW IDEKRALRPM
FRS1     HGLTQLMNDI ADALVAEGFI EVRTPIFISK DALARMTITE DKPLFKQVFW IDEKRALRPM
FRS2     HGLTQLMNDI ADALVAEGFI EVRTPIFISK DALARMTITE DKPLFKQVFW IDEKRALRPM
FRSA     HGLTQLMNDI ADALVAEGFI EVRTPIFISK DALARMTITE DKPLFKQVFW IDEKRALRPM

MaPyRS   LAPNLYSVMR DLRDHTDGPV KIFEMGSCFR KESHSGMHLE EFTMLNLVDM GPRGDATEVL
FRS1     LAPNLYSVMR DLRDHTDGPV KIFEMGSCFR KESHSGMHLE EFTMLALLDM GPRGDATEVL
FRS2     LAPNLYSVMR DLRDHTDGPV KIFEMGSCFR KESHSGMHLE EFTMLALKDM GPRGDATEVL
FRSA     LAPNLYSVMR DLRDHTDGPV KIFEMGSCFR KESHSGMHLE EFTMLALADM GPRGDATEVL

MaPylRS  KNYISVVMKA AGLPDYDLVQ EESDVYKETI DVEINGQEVC SAAVGPHYLD AAHDVHEPWS
FRS1     KNYISVVMKA AGLPDYDLVQ EESDVYKETI DVEINGQEVC SAAVGPHYLD AAHDVHEPWS
FRS2     KNYISVVMKA AGLPDYDLVQ EESDVYKETI DVEINGQEVC SAAVGPHYLD AAHDVHEPWS
FRSA     KNYISVVMKA AGLPDYDLVQ EESDVYKETI DVEINGQEVC SAAVGPHYLD AAHDVHEPWS

MaPylRS  GAGFGLERLL TIREKYSTVK KGGASISYLN GAKIN* 275
FRS1     GAGFGLERLL TIREKYSTVK KGGASISYLN GAKIN* 275
FRS2     GAGFGLERLL TIREKYSTVK KGGASISYLN GAKIN* 275
FRSA     GAGFGLERLL TIREKYSTVK KGGASISYLN GAKIN* 275
```

Supplementary Figure 1. a, Structural alignment of the *M. mazei* PylRS (*Mm*PylRS) catalytic domain (PDB 2ZCE) and *M. alvus* PylRS (*Ma*PylRS) (PDB 6JP2). The two active site residues substituted in FRS1, FRS2, and FRSA are shown explicitly. **b**, Sequence alignment of *Mm*PylRS and *Ma*PylRS using the EMBOSS Needle software[3]. **c,** Sequences of the four enzymes used in this study with differences highlighted in blue.

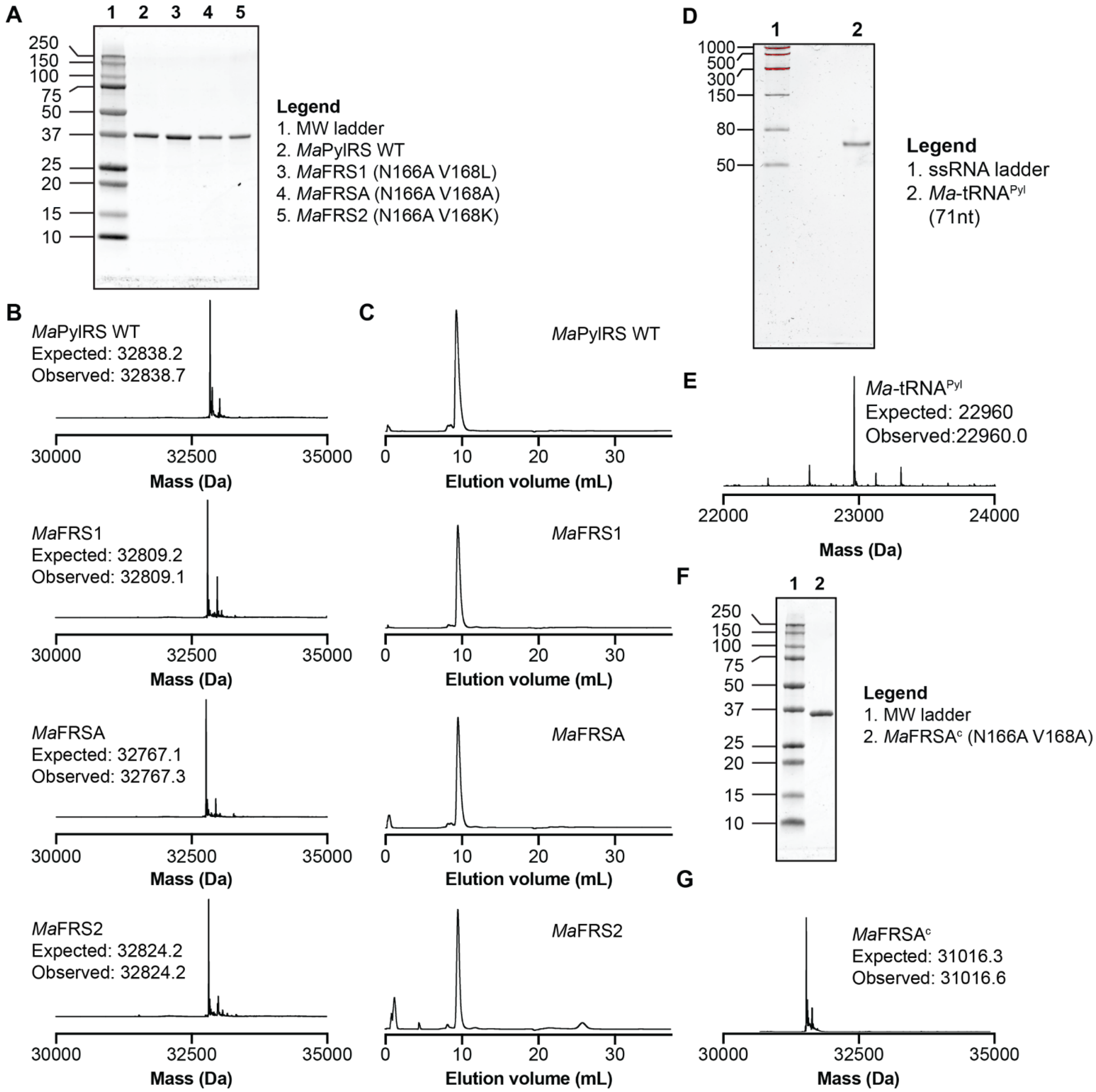

Supplementary Figure 2. a, SDS-PAGE; **b,** LC-MS; and **c,** analytical FPLC chromatograms of purified *Ma*PylRS, *Ma*FRS1, *Ma*FRS2, and *Ma*FRSA used in biochemical experiments. **d,** Urea-PAGE; and **e,** LC-MS analysis of *Ma*-tRNA^{Pyl}. **f,** SDS-PAGE and **g**, LC-MS analysis of *Ma*FRSA used for crystallography. We note that the *Ma*FRSA in panel **b** is extended by an N-terminal His-tag and linker (GSSHHHHHHSSGLVPRGSH-), whereas the *Ma*FRSA used for crystallography in panel **g** only contains an N-terminal GSH scar. The molecular weight standards used in lane 1 of panels **a** and **f** units are shown in units of kDa; the units of the standards used in panel **b** are nucleotides (nt).

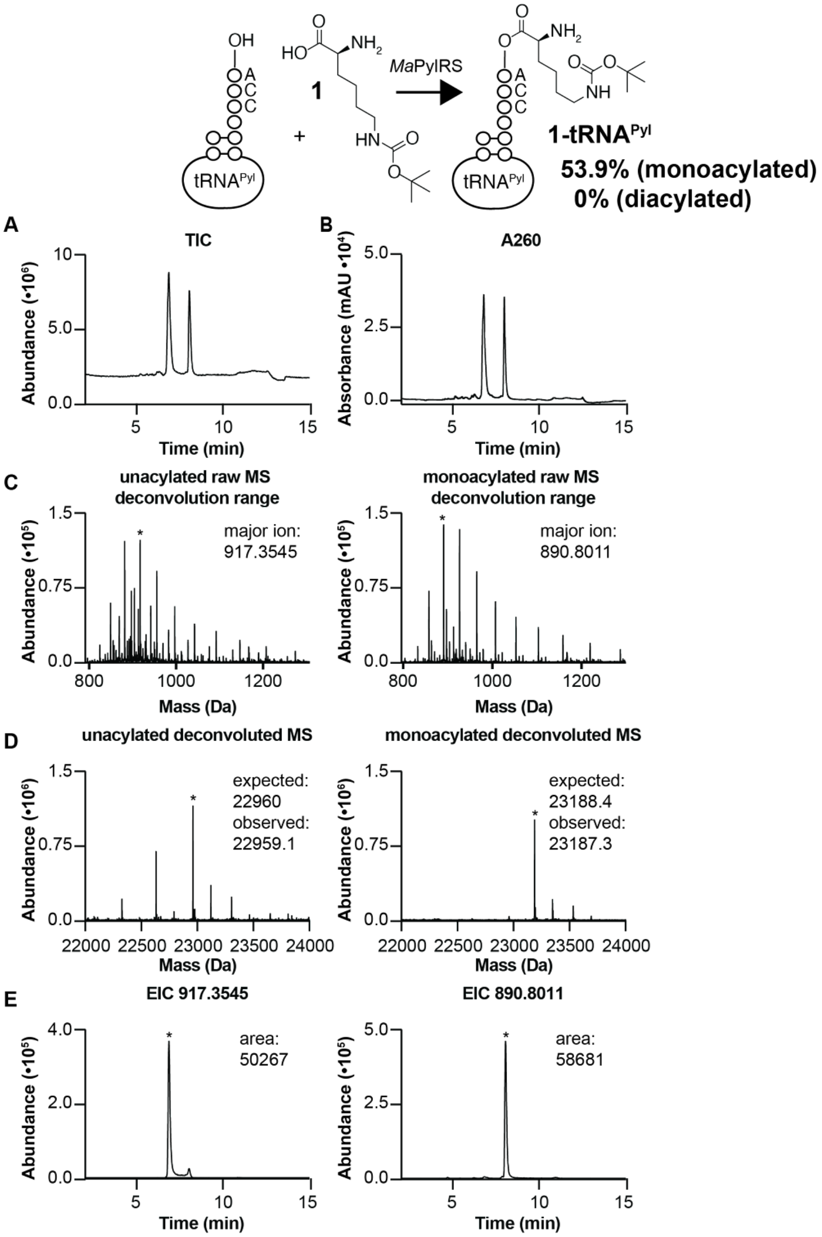

OH
A
C
C
tRNA^Pyl
+
HO
NH2
1
HN
O
O
MaPylRS
O
NH2
O
NH
1-tRNA^Pyl
53.9% (monoacylated)
0% (diacylated)
A
TIC
Abundance (•10^6)
Time (min)
B
A260
Absorbance (mAU •10^4)
Time (min)
C
unacylated raw MS deconvolution range
major ion: 917.3545
Abundance (•10^5)
Mass (Da)
monoacylated raw MS deconvolution range
major ion: 890.8011
D
unacylated deconvoluted MS
expected: 22960
observed: 22959.1
Abundance (•10^6)
monoacylated deconvoluted MS
expected: 23188.4
observed: 23187.3
E
EIC 917.3545
area: 50267
Abundance (•10^5)
EIC 890.8011
area: 58681

Supplementary Figure 3. Analysis of tRNA acylation product mixtures obtained using *Ma*PylRS, *Ma*-tRNA^{Pyl} and monomer **1** as described. **a,** Total ion count and **b,** UV absorbance (260 nm) as a function of elution time. **c,** The raw MS deconvolution range represents the subset of the raw MS data used to determine the deconvoluted mass spectrum of each tRNA species (unacylated or monoacylated). The major ion identified with an asterisk is the most abundant charge state of the tRNA species used for quantification. **d,** Deconvoluted mass spectra generated from the data in (**c**). **e,** Extracted ion chromatograms of the major ions of each tRNA species. The peak corresponding to each tRNA species is noted with an asterisk. The EICs were integrated and the area under the curve (A) was used to determine the overall tRNA acylation yield according to the equation: yield = $[(A_{\text{mono-acylated}} + A_{\text{di-acylated}})/(A_{\text{unacylated}} + A_{\text{mono-acylated}} + A_{\text{di-acylated}})$. The yield shown in this figure is from a representative sample. Average yields from three technical replicates are displayed in Supplementary Table 3.

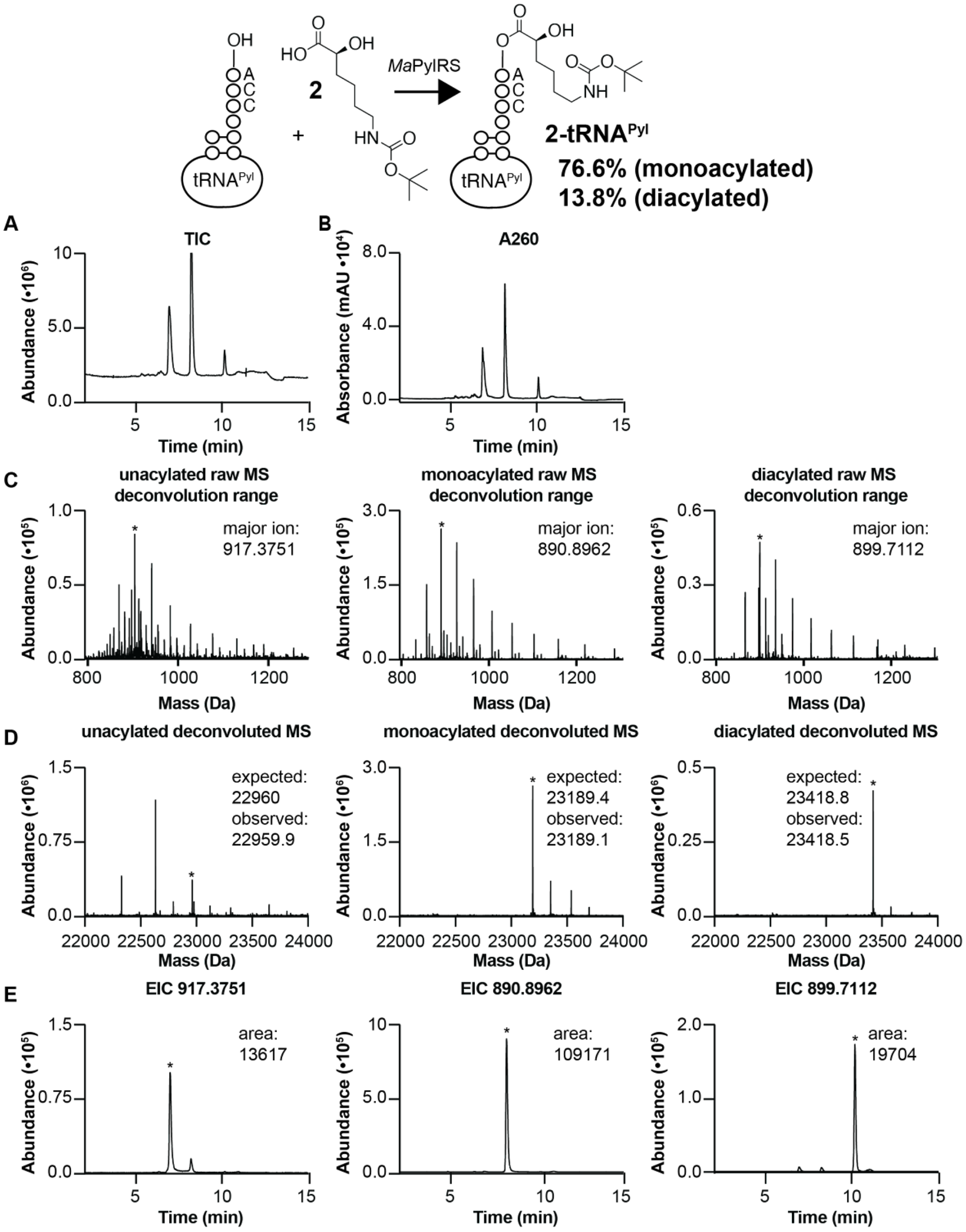

Supplementary Figure 4. Analysis of tRNA product mixtures obtained using *Ma*PylRS, *Ma*-tRNA^{Pyl} and monomer **2** as described. Please refer to the legend for Supplementary Fig. 3 for descriptions of panels **a**-**e**.

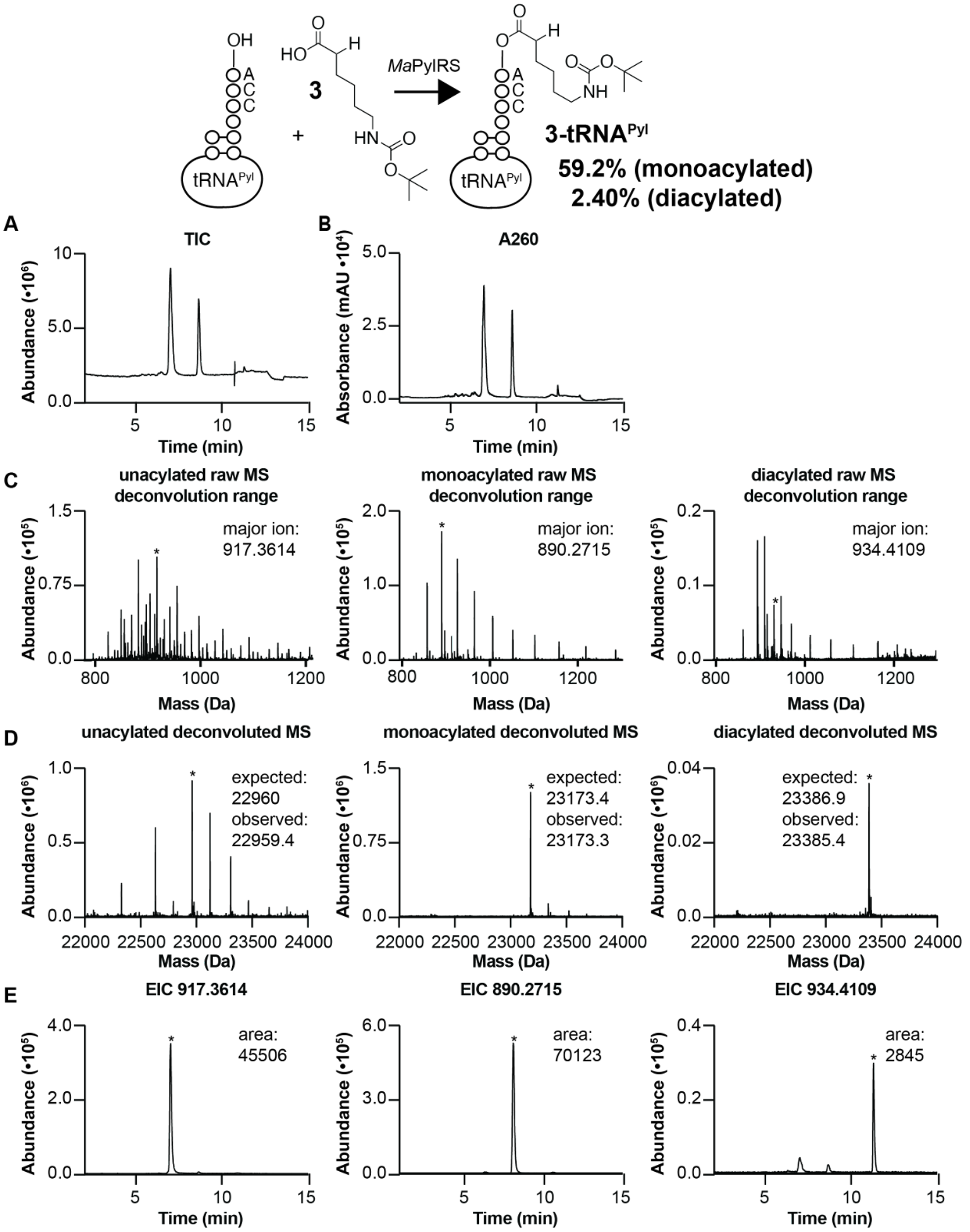

Supplementary Figure 5. Analysis of tRNA product mixtures obtained using *Ma*PylRS, *Ma*-tRNA^{Pyl} and monomer **3** as described. Please refer to the legend for Supplementary Fig. 3 for descriptions of panels **a**-**e**.

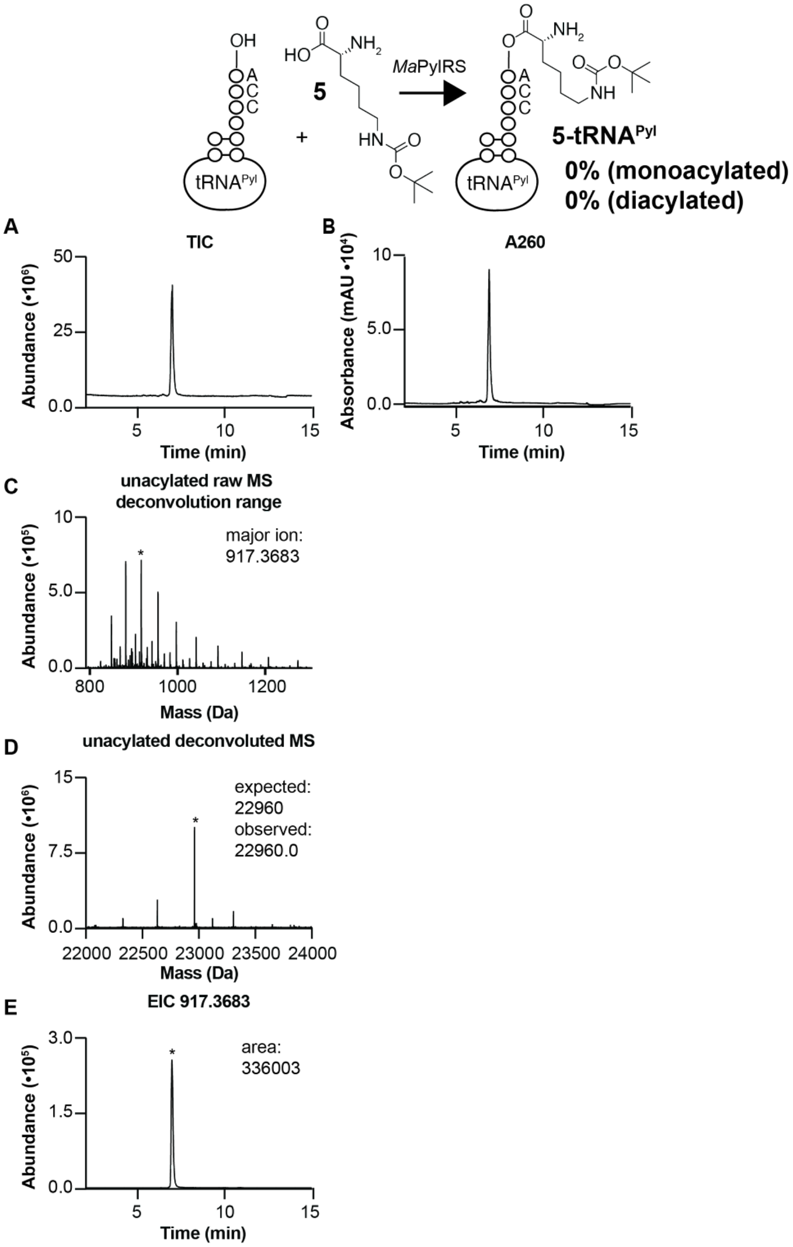

Supplementary Figure 6. Analysis of tRNA product mixtures obtained using *Ma*PylRS, *Ma*-tRNA^{Pyl} and monomer **5** as described. Please refer to the legend for Supplementary Fig. 3 for descriptions of panels **a**-**e**.

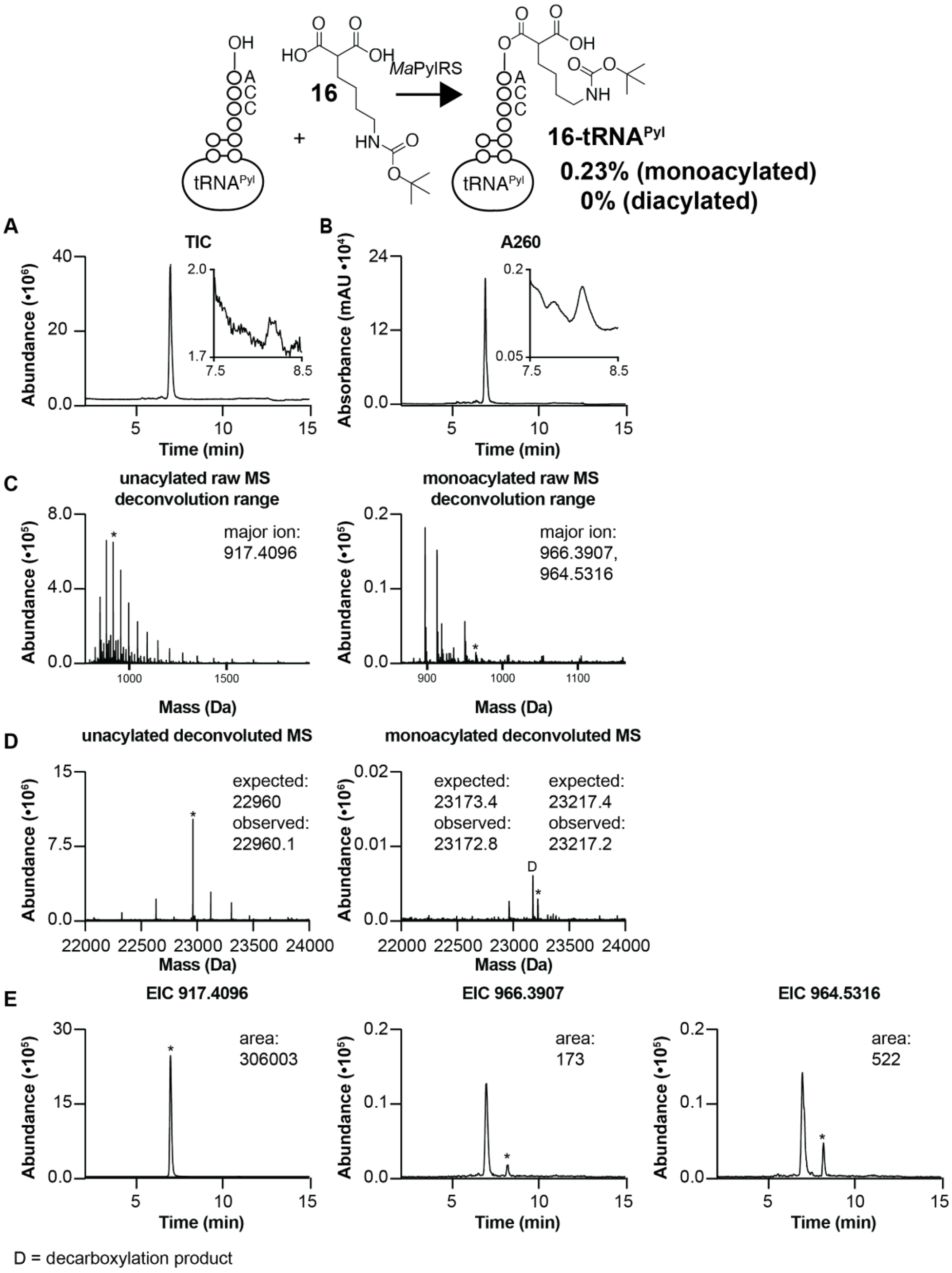

Supplementary Figure 7. Analysis of tRNA product mixtures obtained using *Ma*PylRS, *Ma*-tRNA^{Pyl} and monomer **16** as described. Please refer to the legend for Supplementary Fig. 3 for descriptions of panels **a**-**e** In panel **c**, the major ion for both the base mass and the decarboxylation product are listed. In panel **d**, the decarboxylation product mass is denoted by a D. The areas under the curve in panel **e** for the base and decarboxylation product masses were combined to calculate the overall acylation yield.

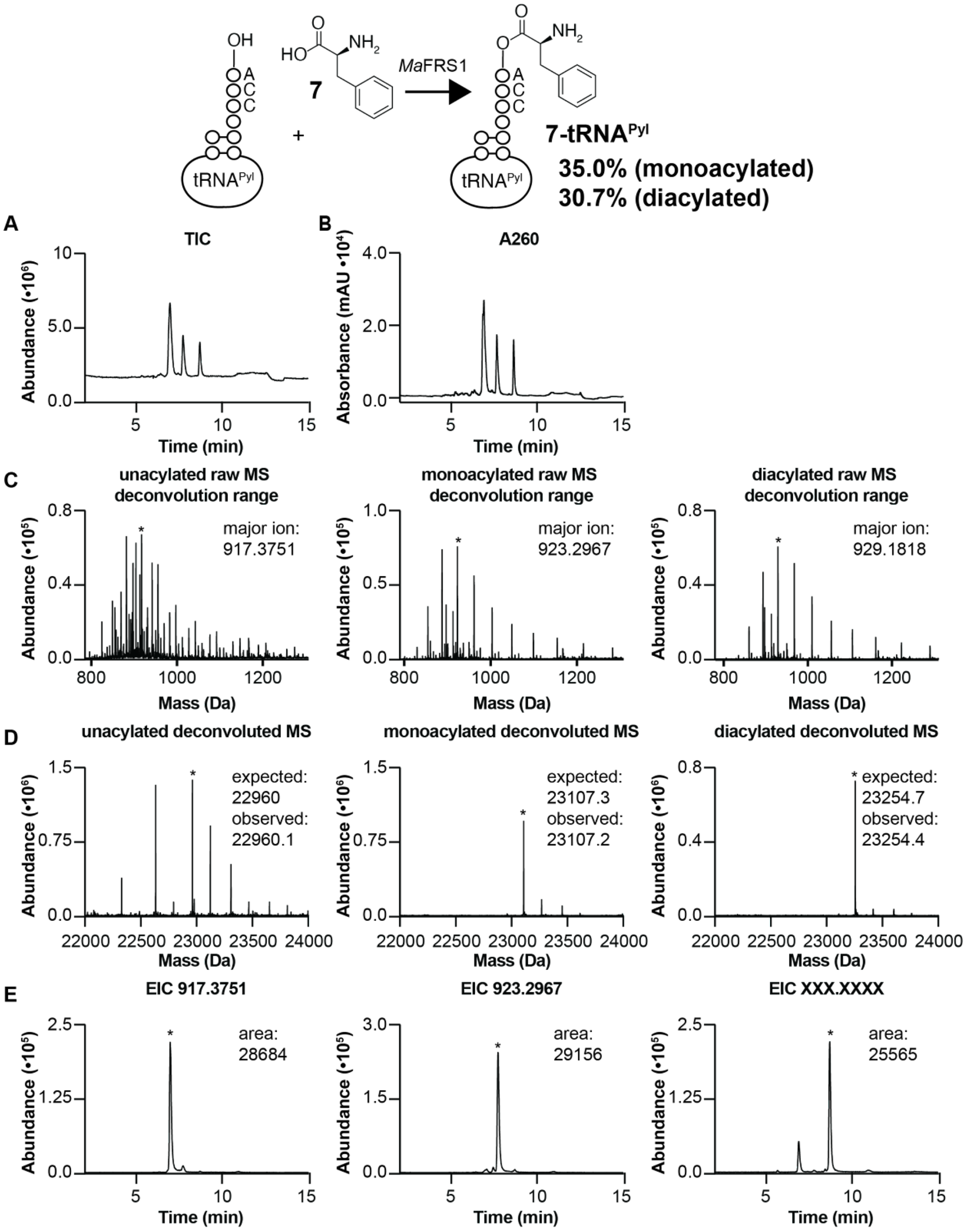

Supplementary Figure 8. Analysis of tRNA product mixtures obtained using *Ma*FRS1, *Ma*-tRNA^{Pyl} and monomer **7** as described. Please refer to the legend for Supplementary Fig. 3 for descriptions of panels **a**-**e**.

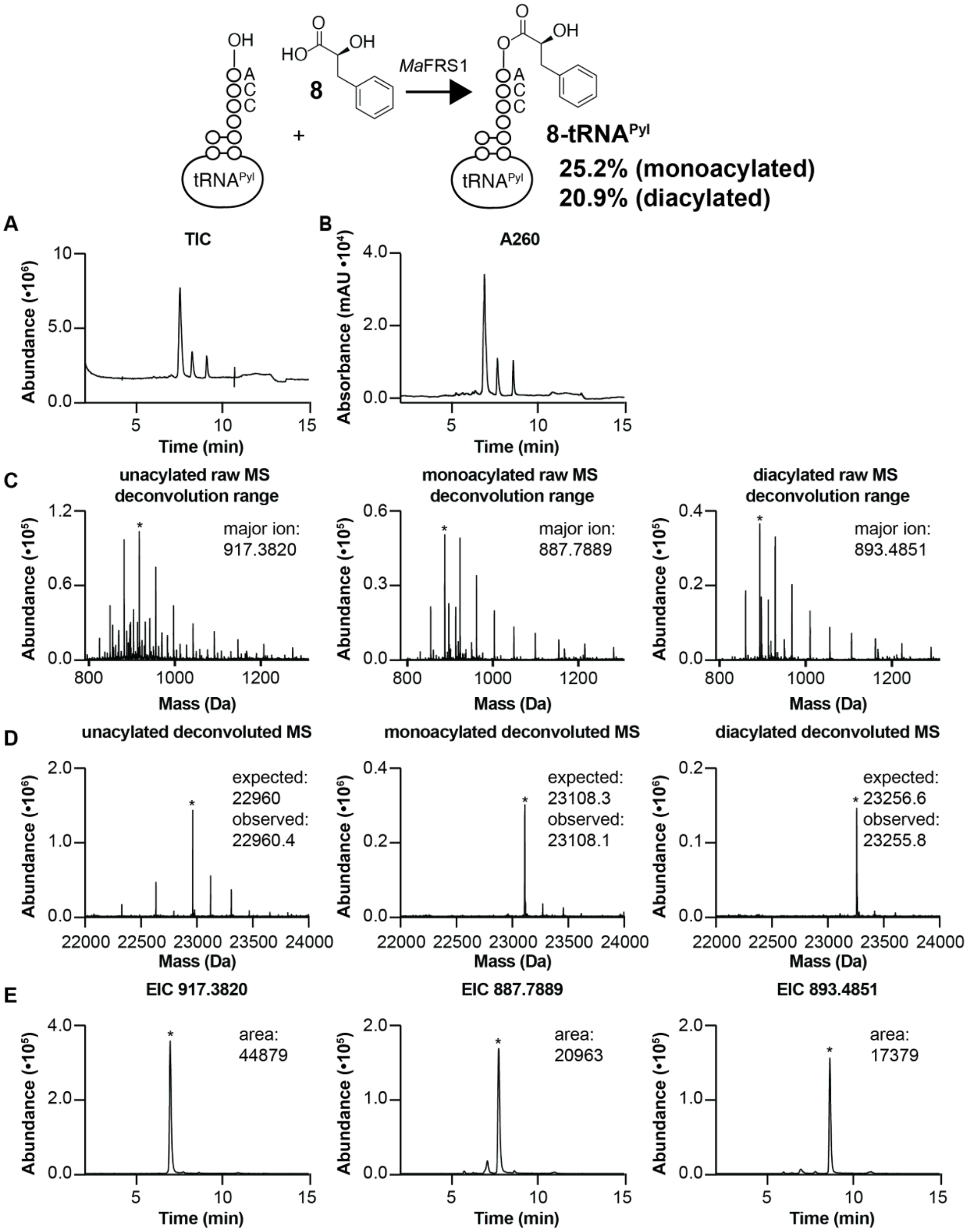

Supplementary Figure 9. Analysis of tRNA product mixtures obtained using *Ma*FRS1, *Ma*-tRNA^{Pyl} and monomer **8** as described. Please refer to the legend for Supplementary Fig. 3 for descriptions of panels **a**-**e**.

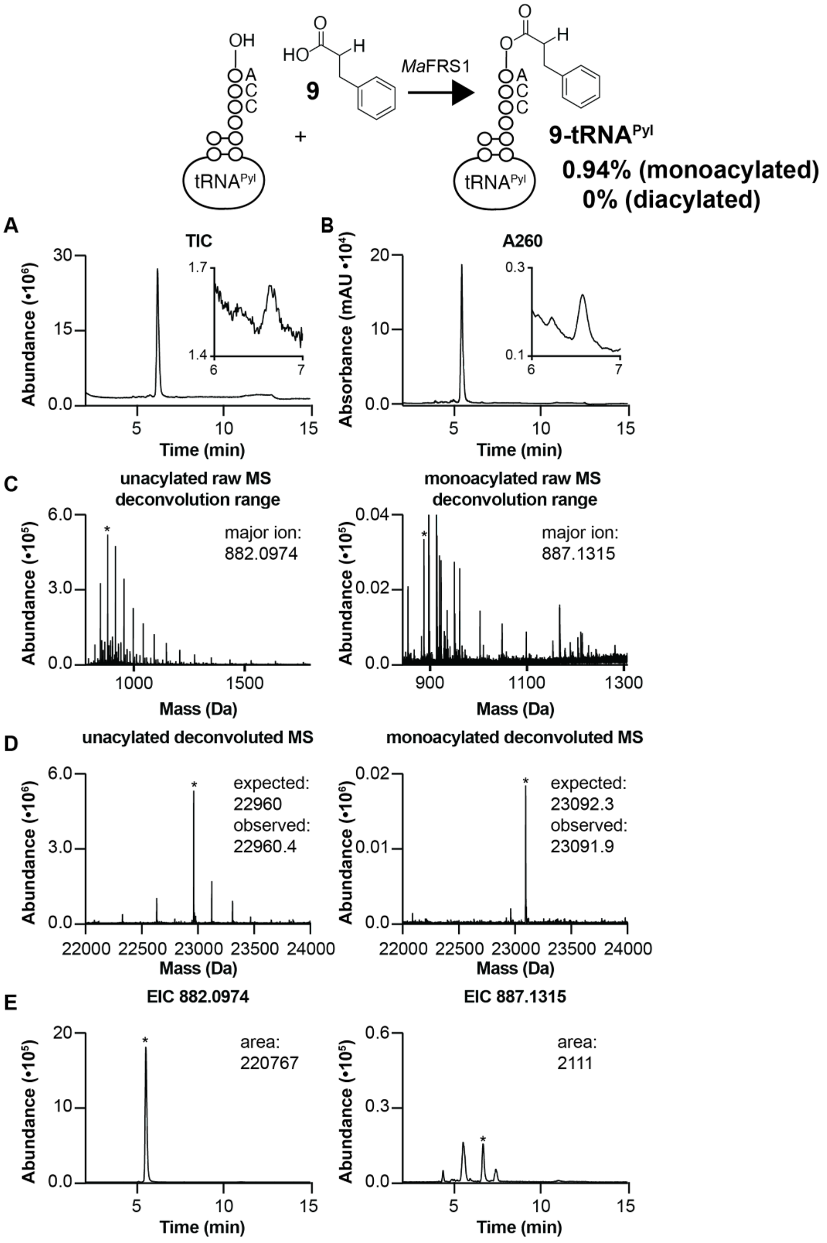

Supplementary Figure 10. Analysis of tRNA product mixtures obtained using *Ma*FRS1, *Ma*-tRNA^{Pyl} and monomer **9** as described. Please refer to the legend for Supplementary Fig. 3 for descriptions of panels **a**-**e**.

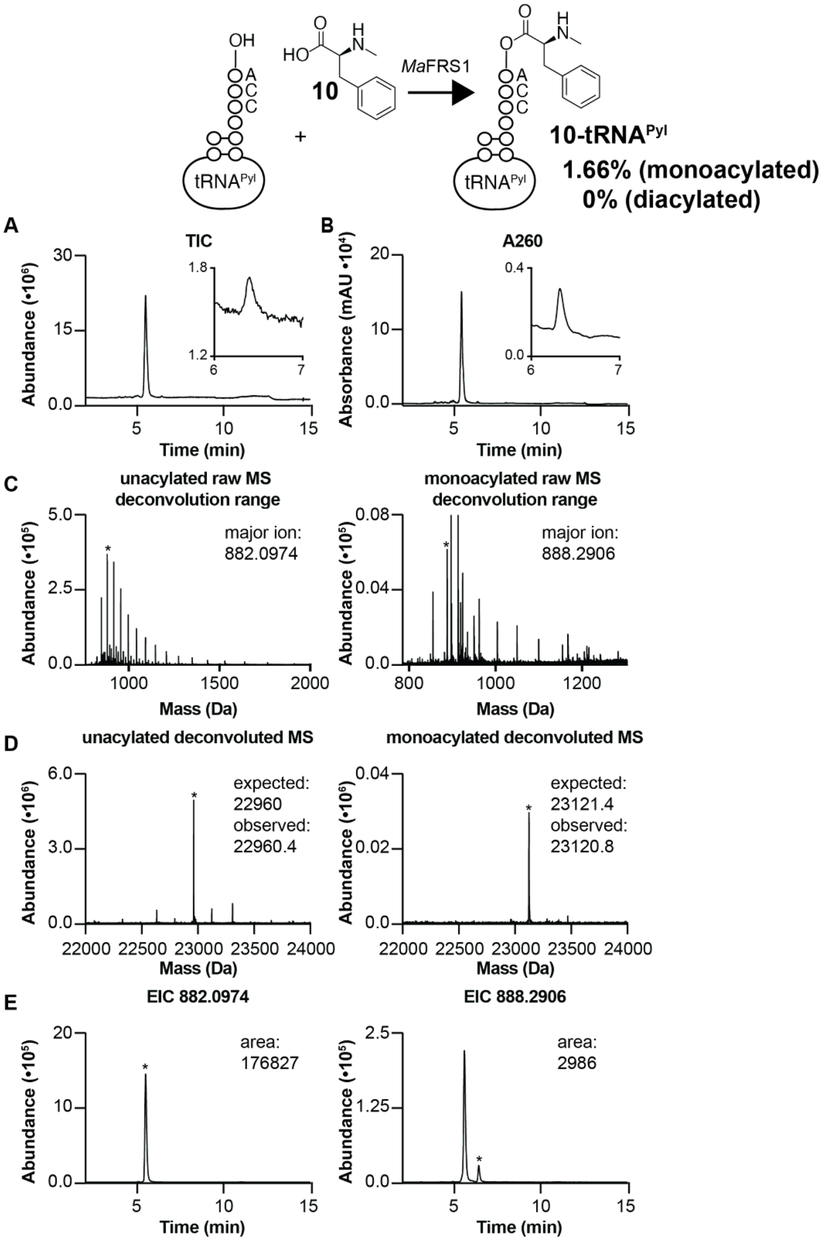

Supplementary Figure 11. Analysis of tRNA product mixtures obtained using *Ma*FRS1, *Ma*-tRNA^{Pyl} and monomer **10** as described. Please refer to the legend for Supplementary Fig. 3 for descriptions of panels **a**-**e**.

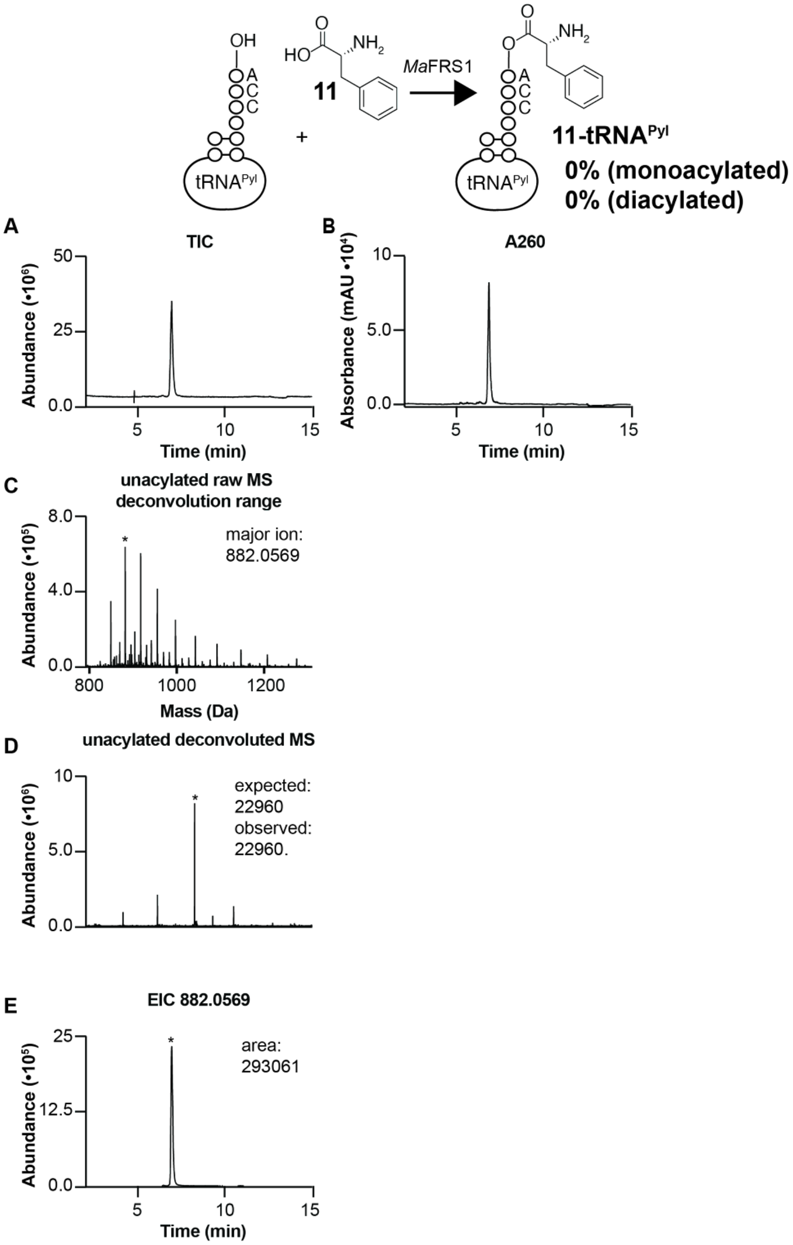

Supplementary Figure 12. Analysis of tRNA product mixtures obtained using *Ma*FRS1, *Ma*-tRNA^{Pyl} and monomer **11** as described. Please refer to the legend for Supplementary Fig. 3 for descriptions of panels **a**-**e**.

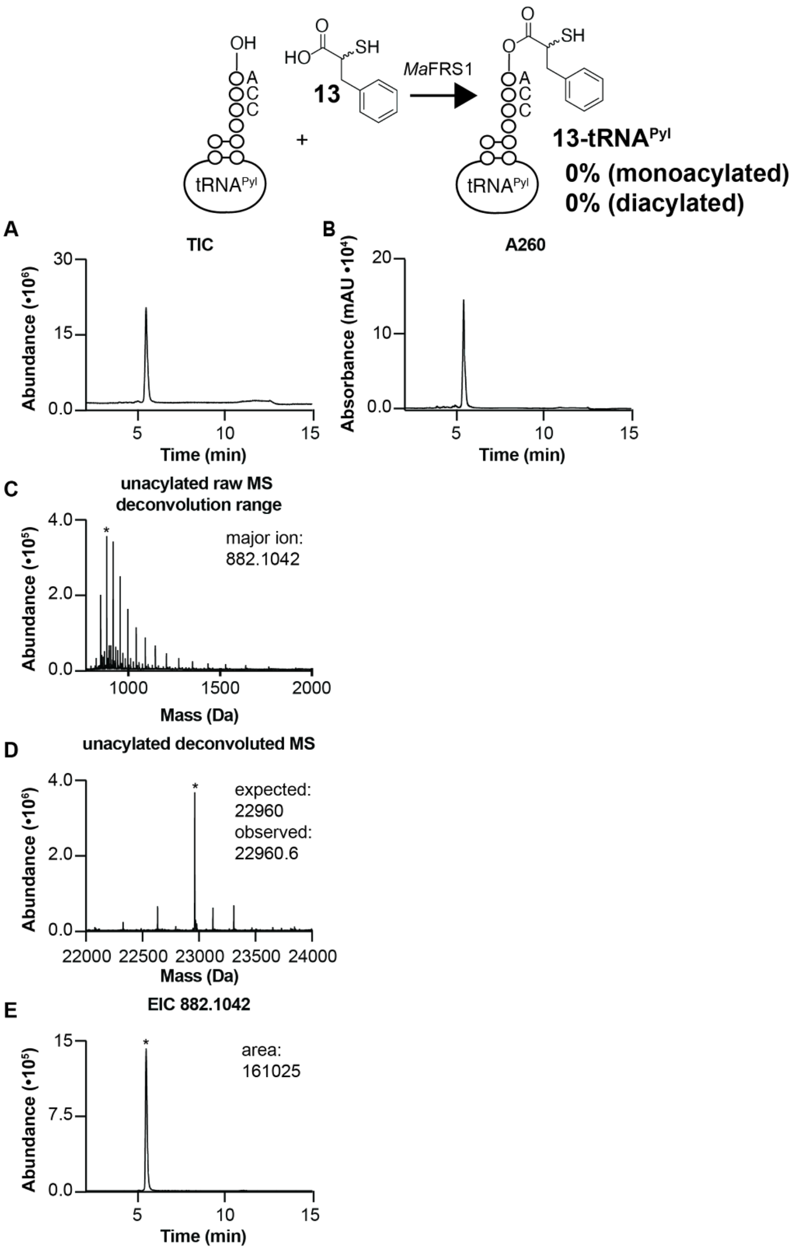

Supplementary Figure 13. Analysis of tRNA product mixtures obtained using *Ma*FRS1, *Ma*-tRNA^{Pyl} and monomer **13** as described. Please refer to the legend for Supplementary Fig. 3 for descriptions of panels **a**-**e**.

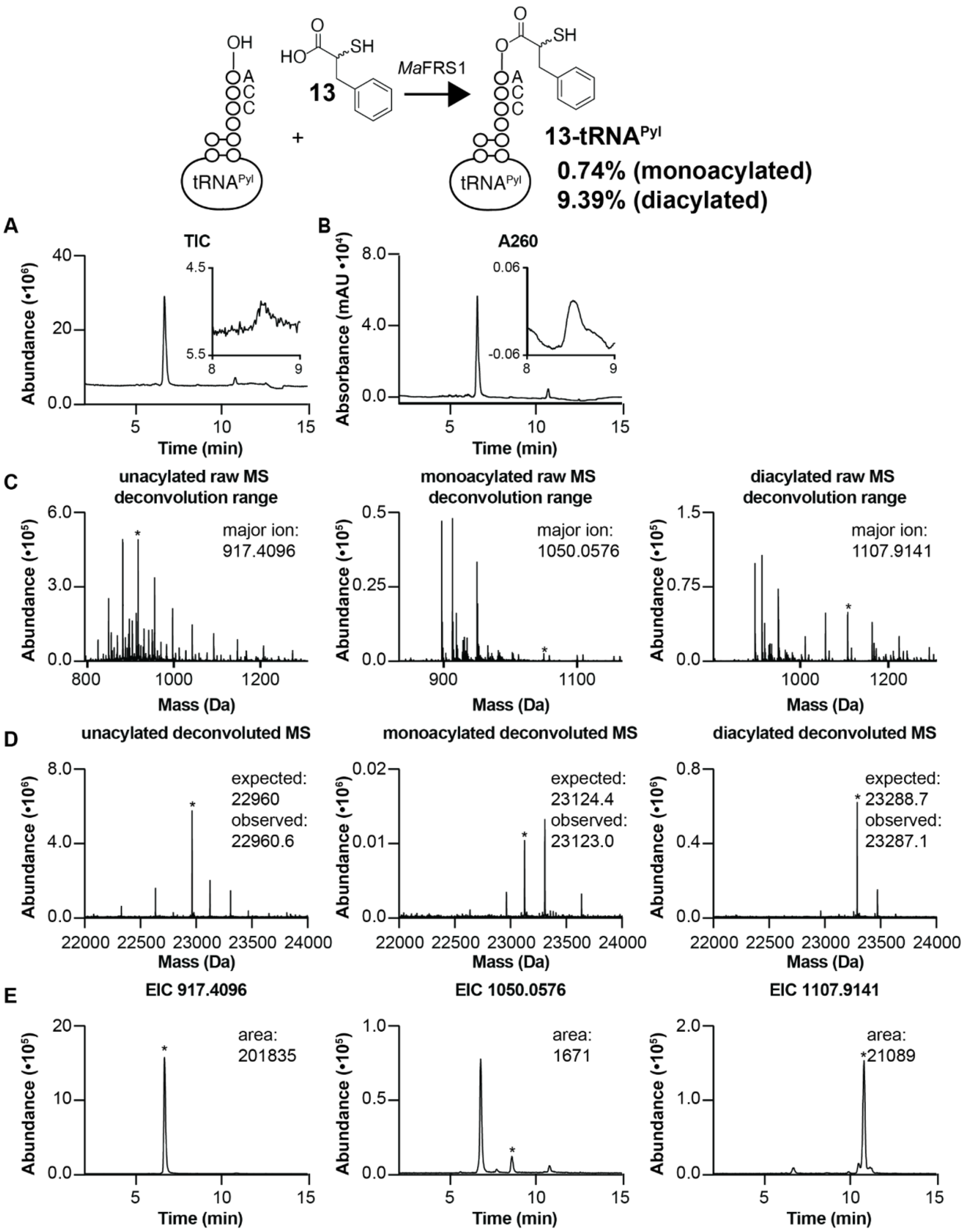

Supplementary Figure 14. Analysis of tRNA product mixtures obtained using *Ma*FRS1, *Ma*-tRNA^{Pyl} and monomer **13** as described. Please refer to the legend for Supplementary Fig. 3 for descriptions of panels **a**-**e**. In this case, acylation was performed using *Ma*FRS1:*Ma*-tRNA^{Pyl} ratio of 1:2.

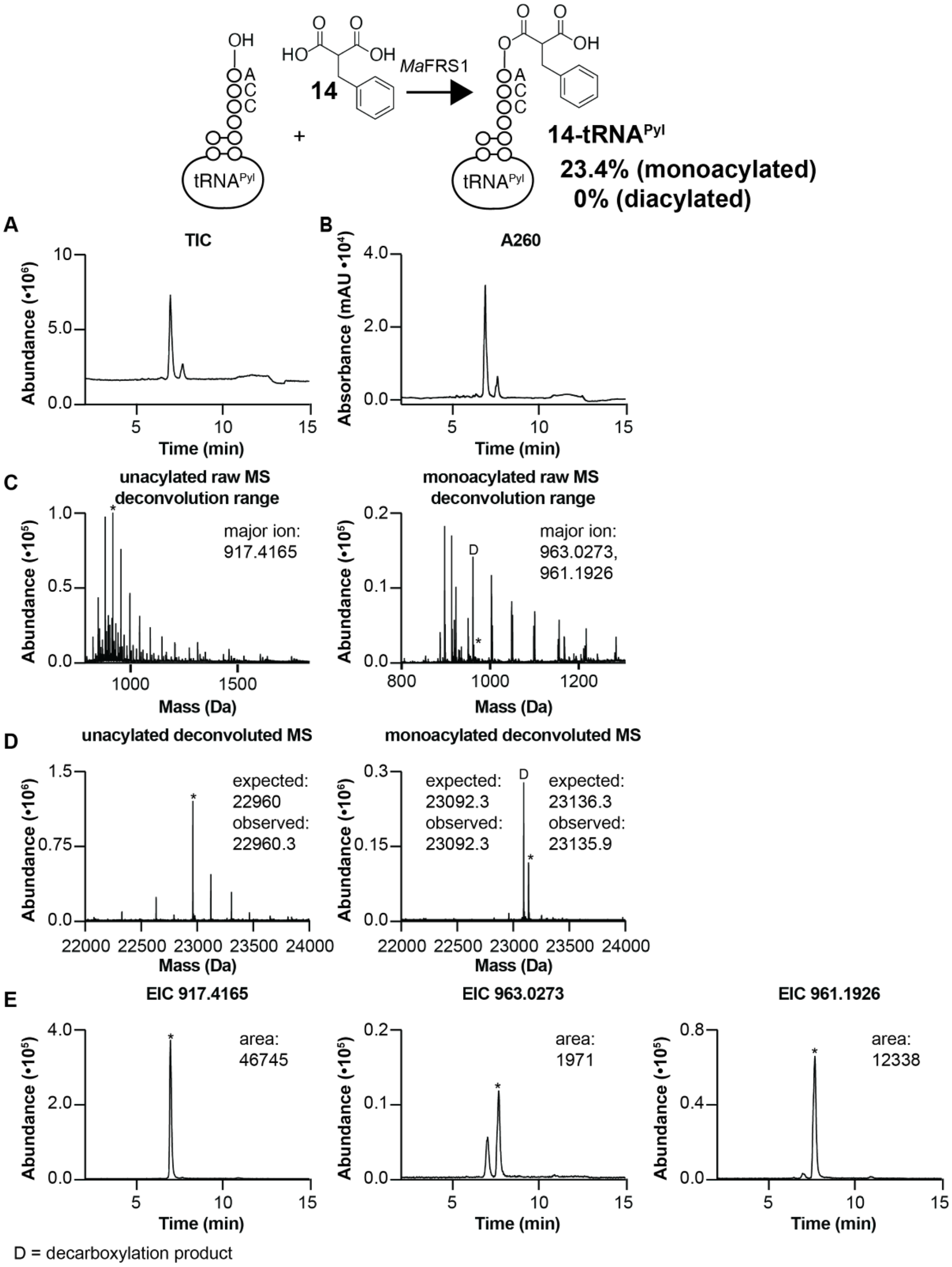

Supplementary Figure 15. Analysis of tRNA product mixtures obtained using *Ma*FRS1, *Ma*-tRNA^{Pyl} and monomer **14** as described. Please refer to the legend for Supplementary Fig. 3 for descriptions of panels **a-e** and the legend of Supplementary Fig. 7 for a note on the decarboxylation products observed in the mass spectra.

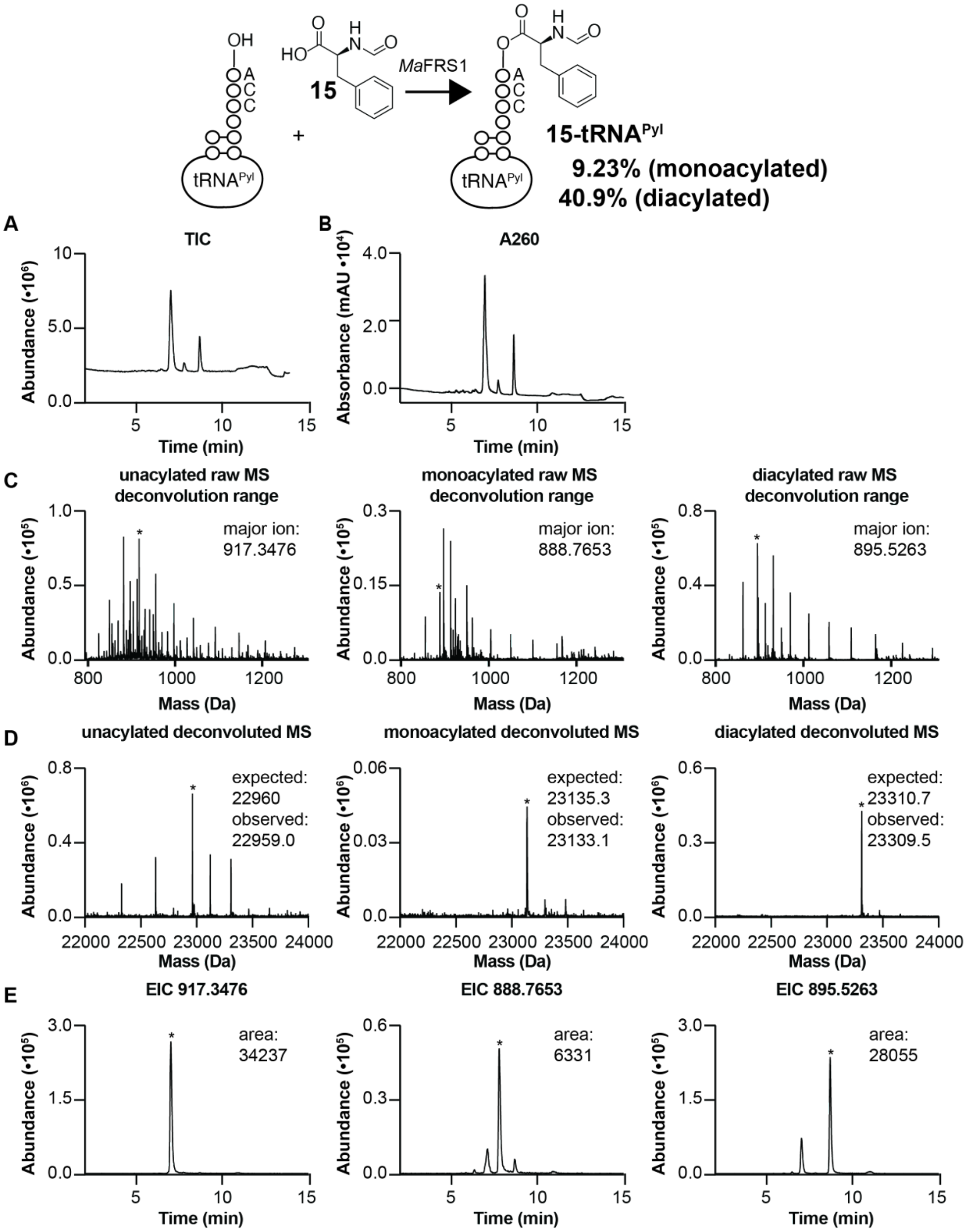

Supplementary Figure 16. Analysis of tRNA product mixtures obtained using *Ma*FRS1, *Ma*-tRNA^{Pyl} and monomer **15** as described. Please refer to the legend for Supplementary Fig. 3 for descriptions of panels **a**-**e**.

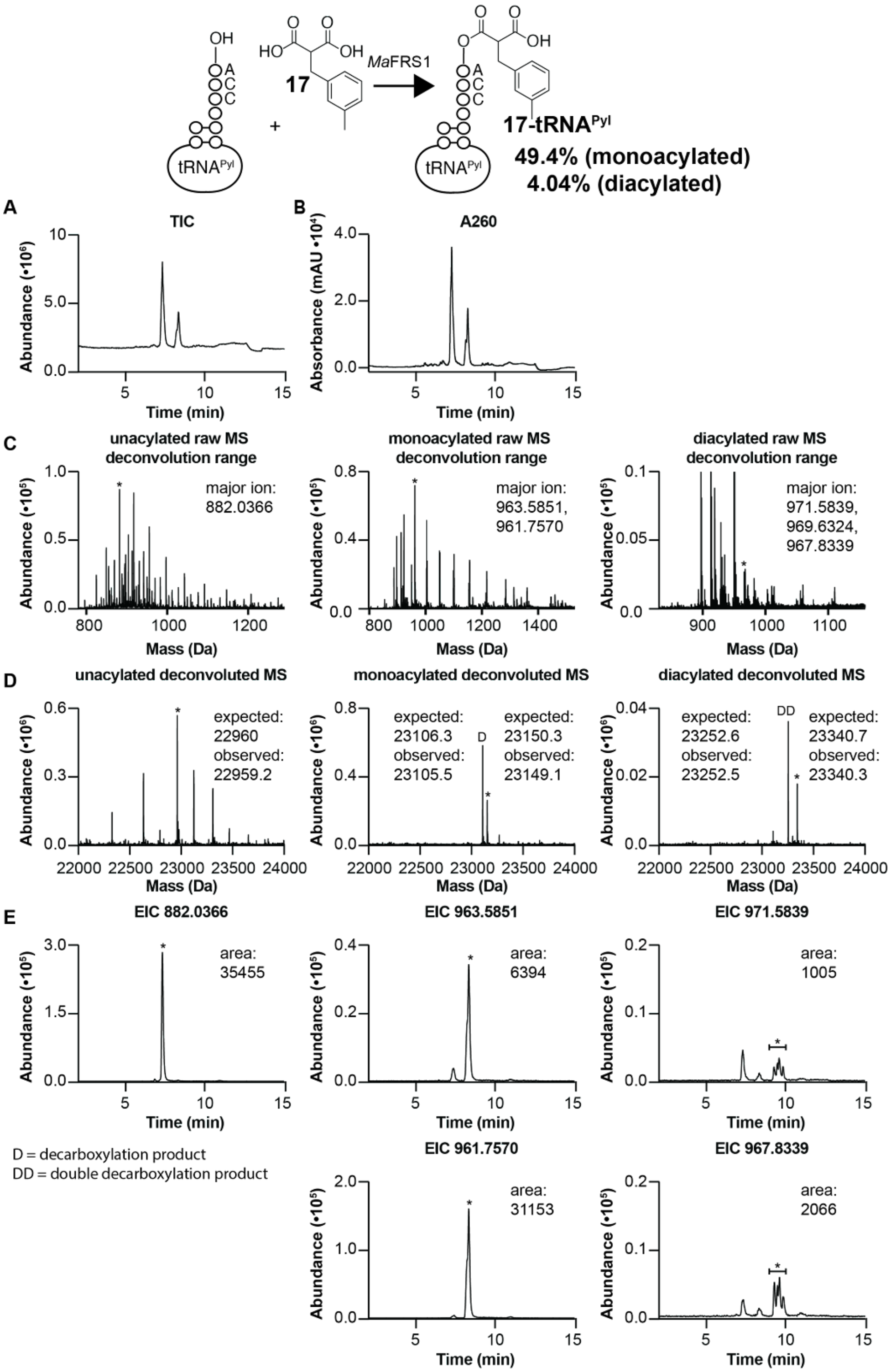

Supplementary Figure 17. Analysis of tRNA product mixtures obtained using *Ma*FRS1, *Ma*-tRNA^{Pyl} and monomer **17** as described. Please refer to the legend for Supplementary Fig. 3 for descriptions of panels **a**-**e** and the legend of Supplementary Fig. 7 for a note on the decarboxylation products observed in the mass spectra.

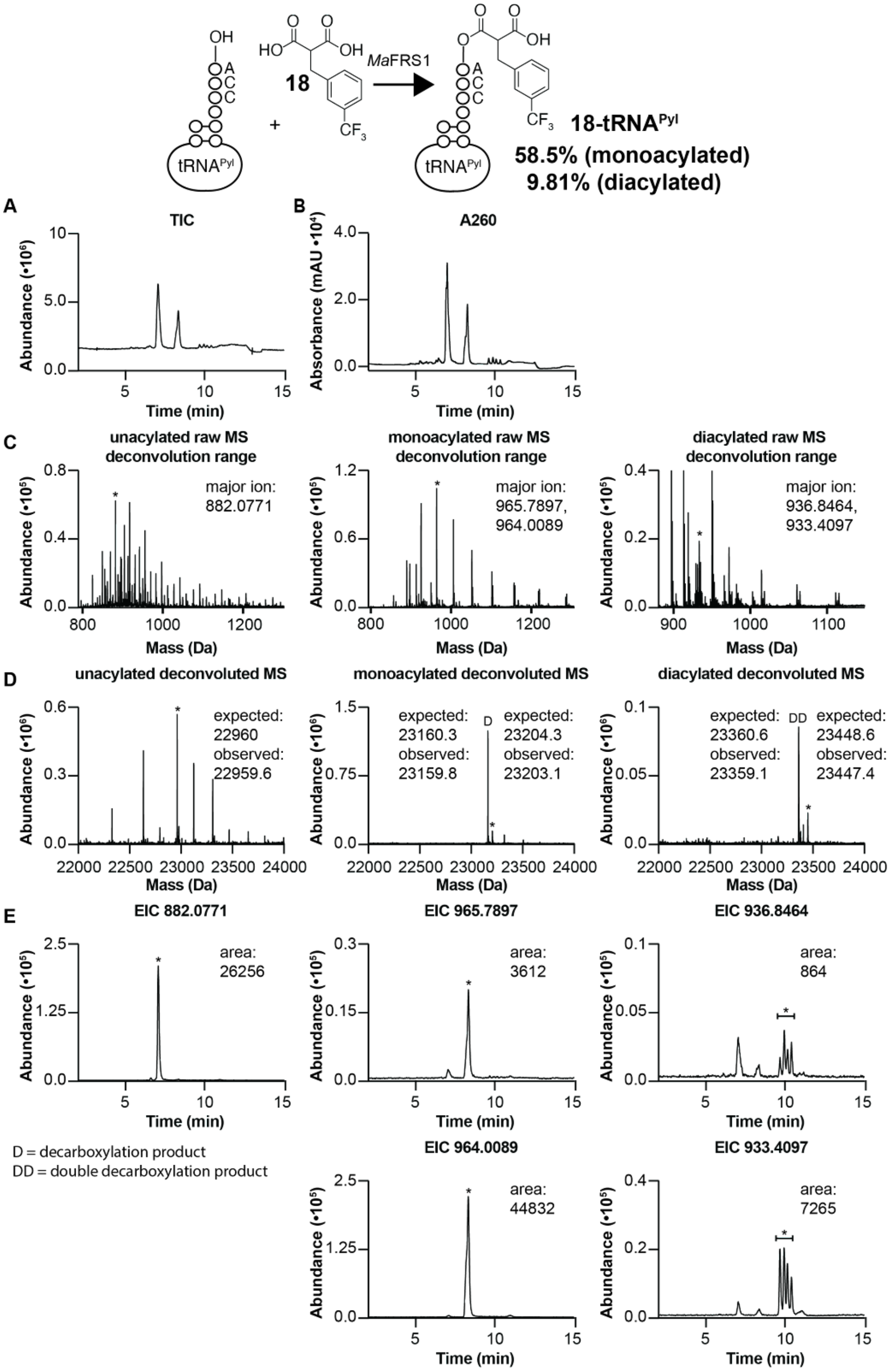

Supplementary Figure 18. Analysis of tRNA product mixtures obtained using *Ma*FRS1, *Ma*-tRNA^{Pyl} and monomer **18** as described. Please refer to the legend for Supplementary Fig. 3 for descriptions of panels **a**-**e** and the legend of Supplementary Fig. 7 for a note on the decarboxylation products observed in the mass spectra.

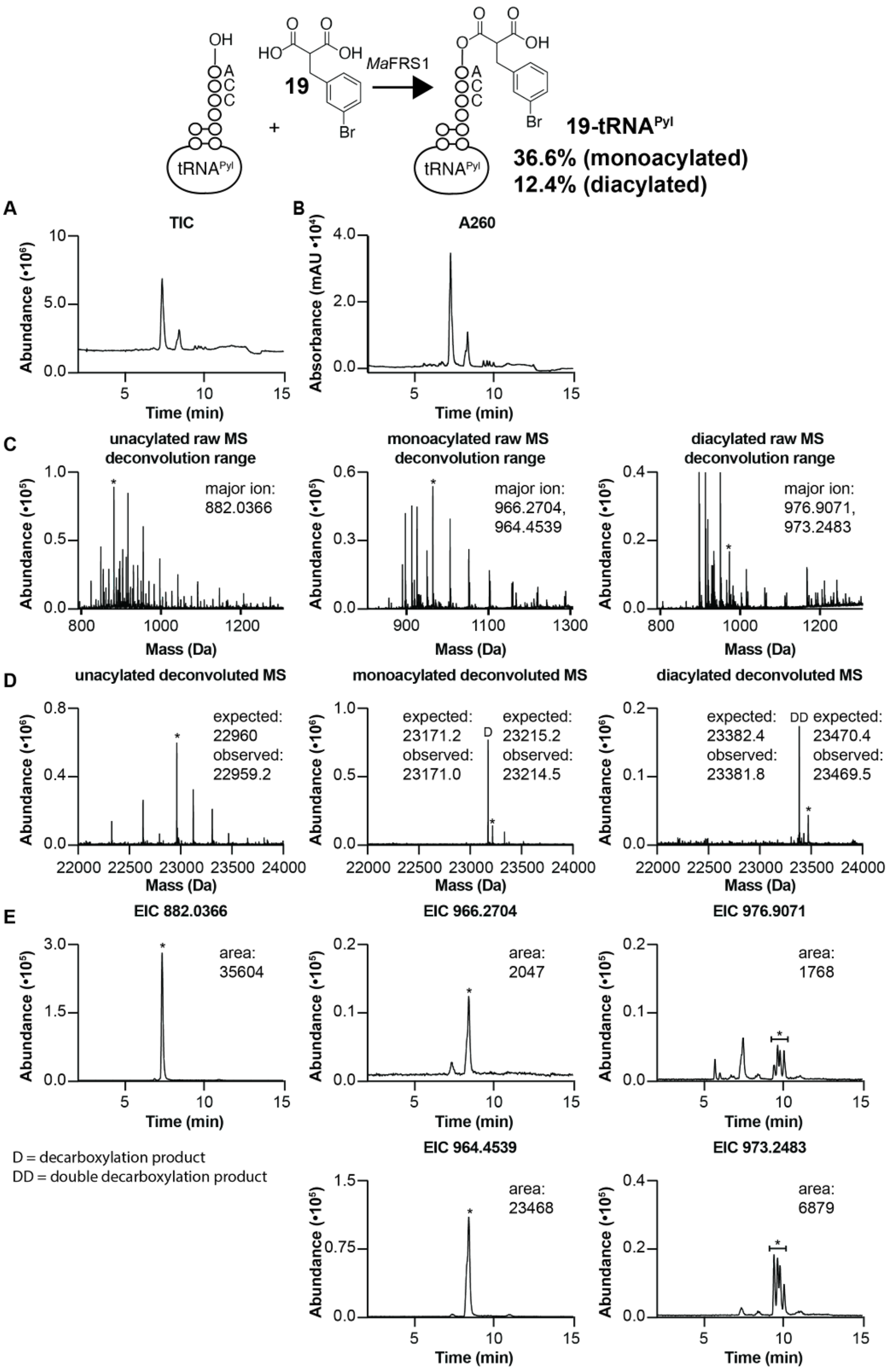

Supplementary Figure 19. Analysis of tRNA product mixtures obtained using *Ma*FRS1, *Ma*-tRNA^{Pyl} and monomer **19** as described. Please refer to the legend for Supplementary Fig. 3 for descriptions of panels **a-e** and a note on the decarboxylation products observed in the mass spectra.

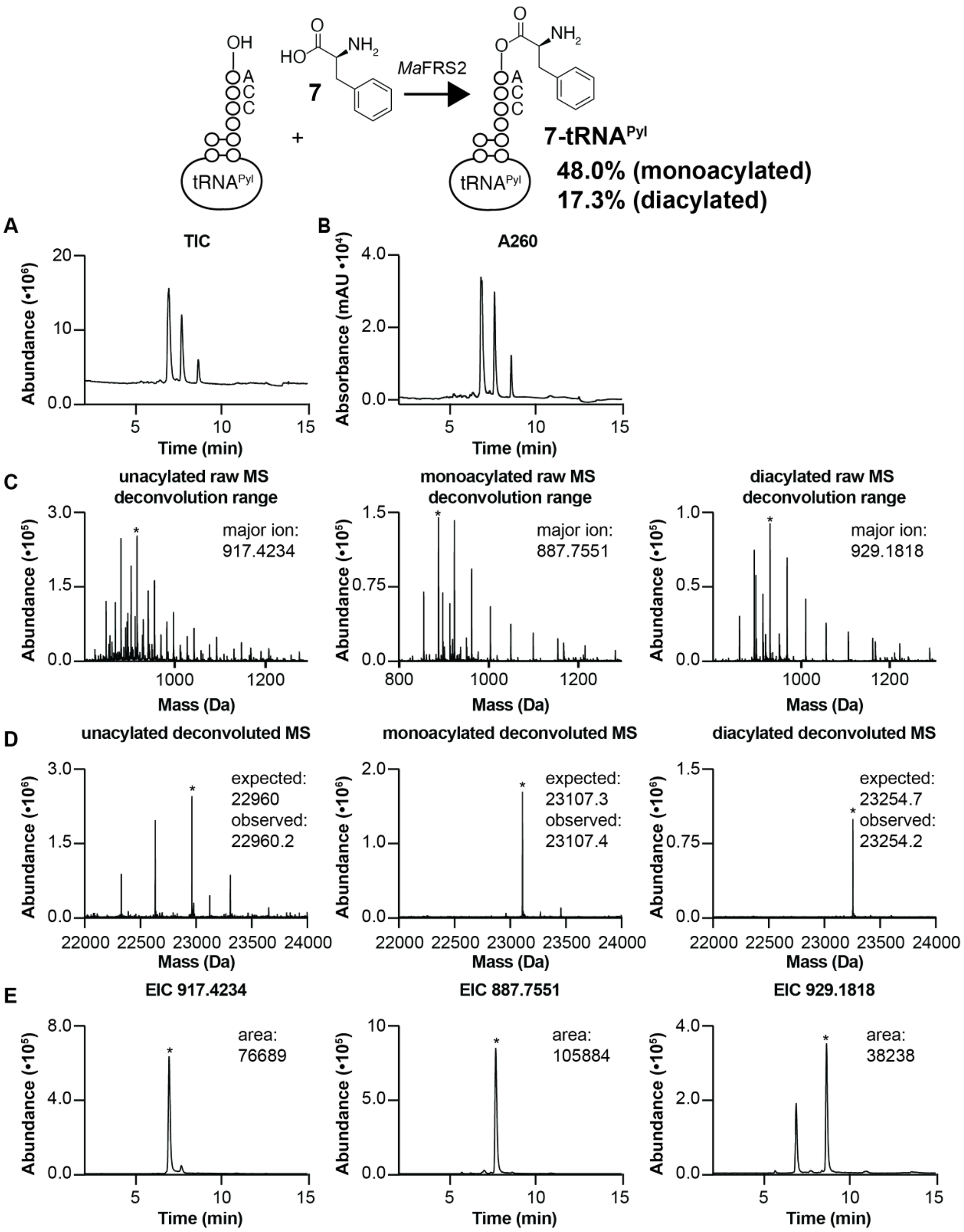

Supplementary Figure 20. Analysis of tRNA product mixtures obtained using *Ma*FRS2, *Ma*-tRNA^{Pyl} and monomer **7** as described. Please refer to the legend for Supplementary Fig. 3 for descriptions of panels **a**-**e**.

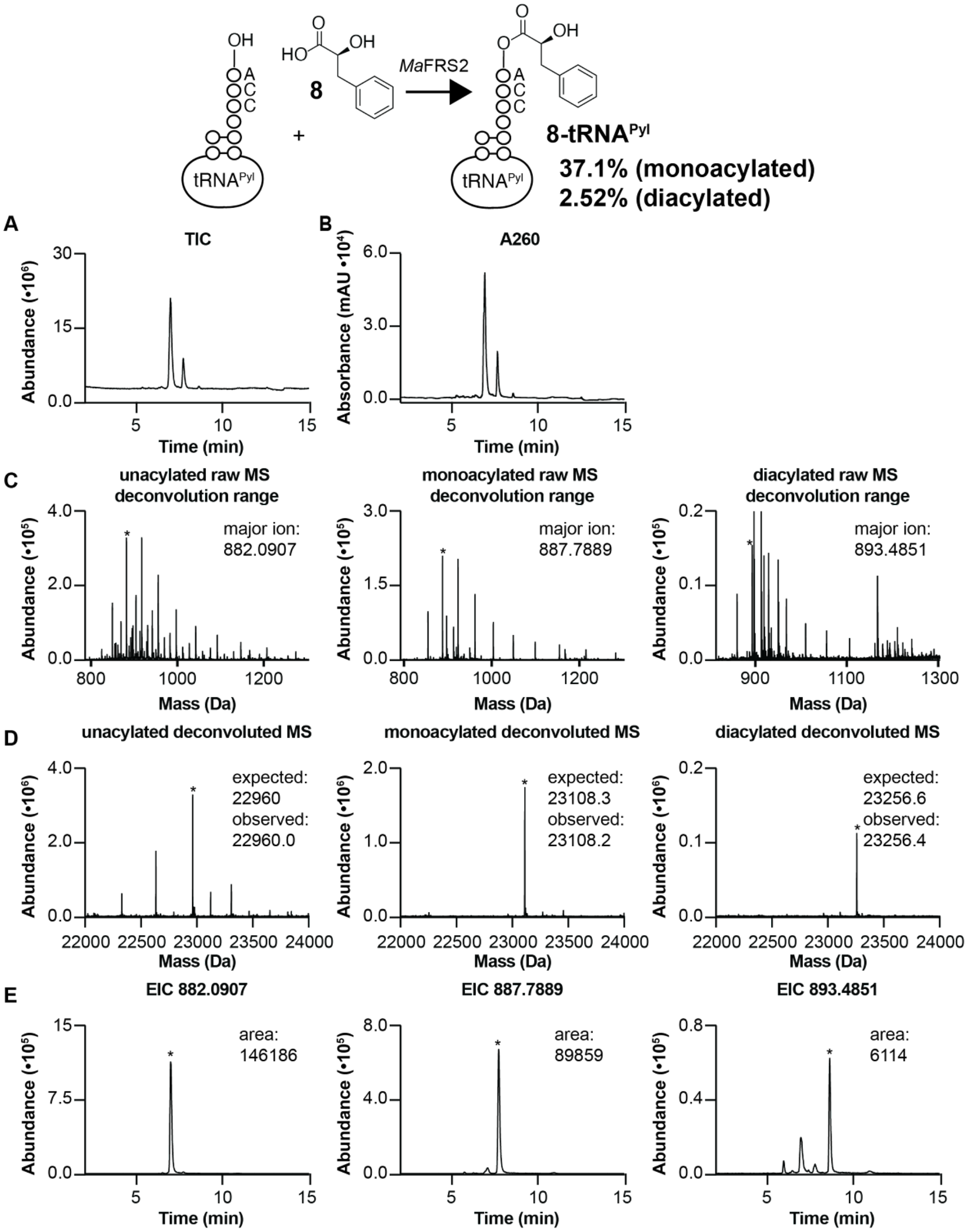

Supplementary Figure 21. Analysis of tRNA product mixtures obtained using *Ma*FRS2, *Ma*-tRNA^{Pyl} and monomer **8** as described. Please refer to the legend for Supplementary Fig. 3 for descriptions of panels **a**-**e**.

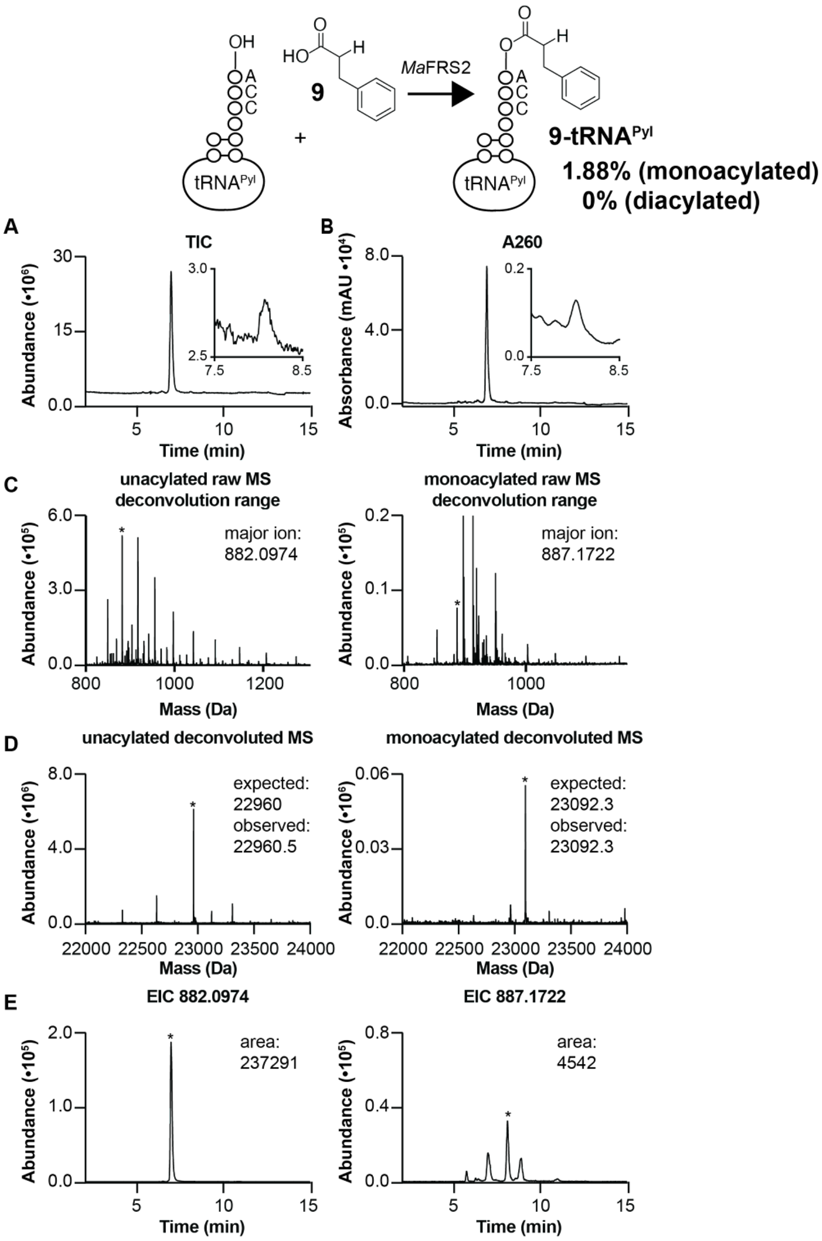

Supplementary Figure 22. Analysis of tRNA product mixtures obtained using *Ma*FRS2, *Ma*-tRNA^{Pyl} and monomer **9** as described. Please refer to the legend for Supplementary Fig. 3 for descriptions of panels **a**-**e**.

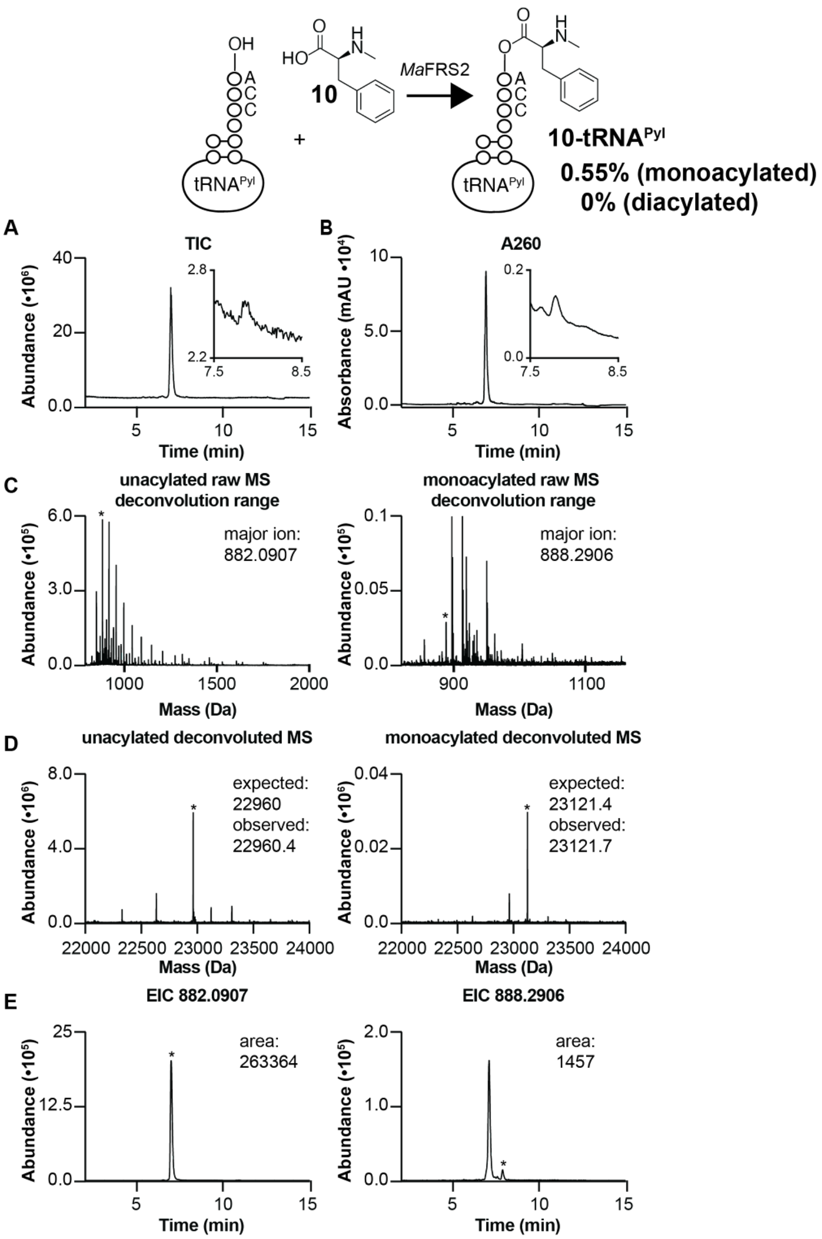

Supplementary Figure 23. Analysis of tRNA product mixtures obtained using *Ma*FRS2, *Ma*-tRNA^{Pyl} and monomer **10** as described. Please refer to the legend for Supplementary Fig. 3 for descriptions of panels **a**-**e**.

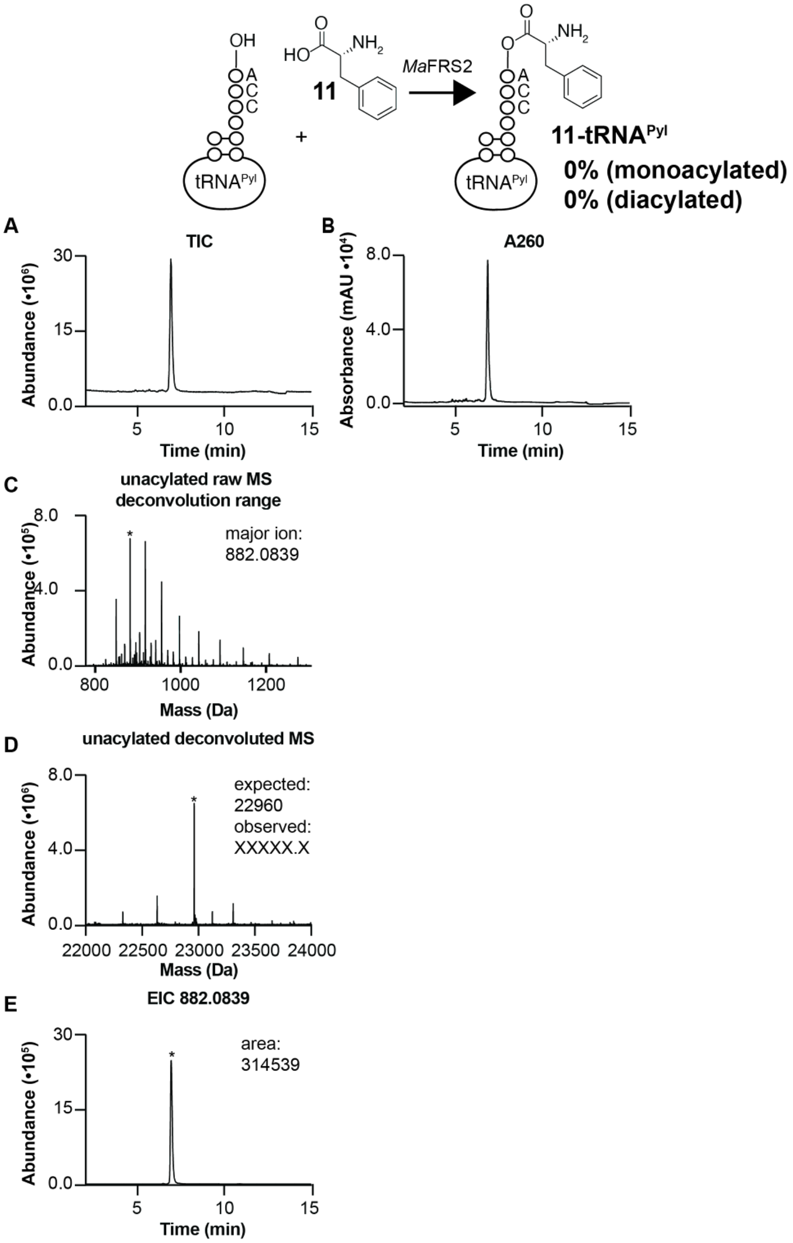

Supplementary Figure 24. Analysis of tRNA product mixtures obtained using *Ma*FRS2, *Ma*-tRNA^{Pyl} and monomer **11** as described. Please refer to the legend for Supplementary Fig. 3 for descriptions of panels **a**-**e**.

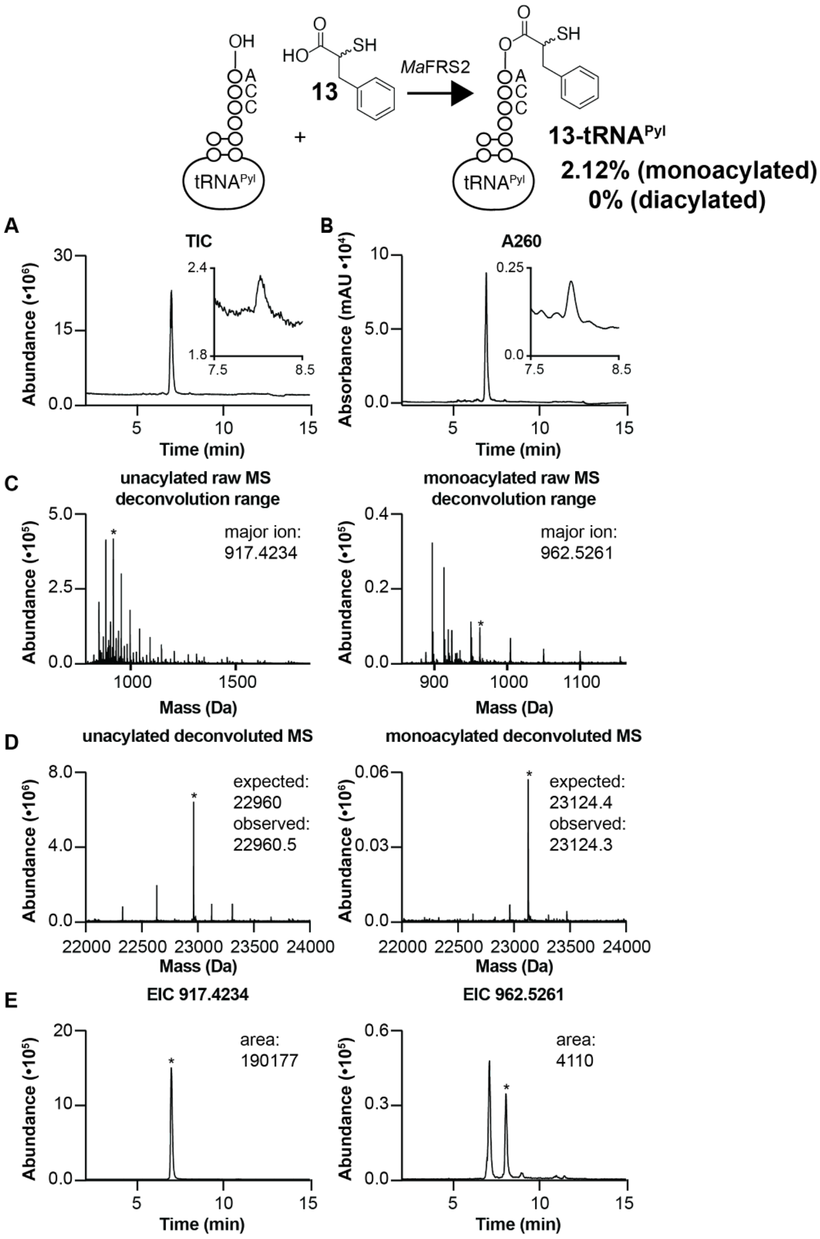

Supplementary Figure 25. Analysis of tRNA product mixtures obtained using *Ma*FRS2, *Ma*-tRNA^{Pyl} and monomer **13** as described. Please refer to the legend for Supplementary Fig. 3 for descriptions of panels **a**-**e**.

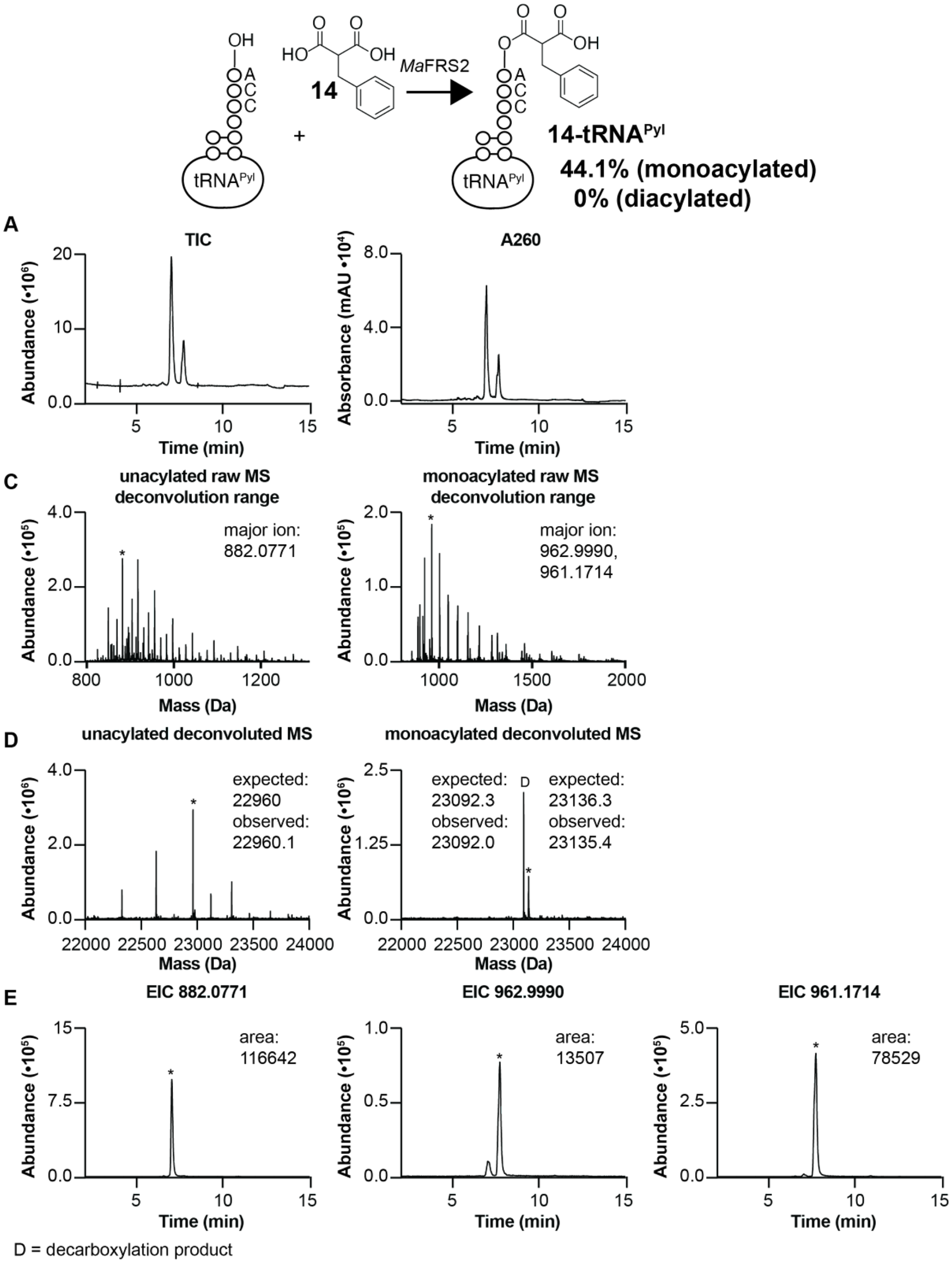

Supplementary Figure 26. Analysis of tRNA product mixtures obtained using *Ma*FRS2, *Ma*-tRNA^{Pyl} and monomer **14** as described. Please refer to the legends for Supplementary Fig. 3 for descriptions of panels **a-e** and Supplementary Fig. 7 for a note on the decarboxylation products observed in the mass spectra.

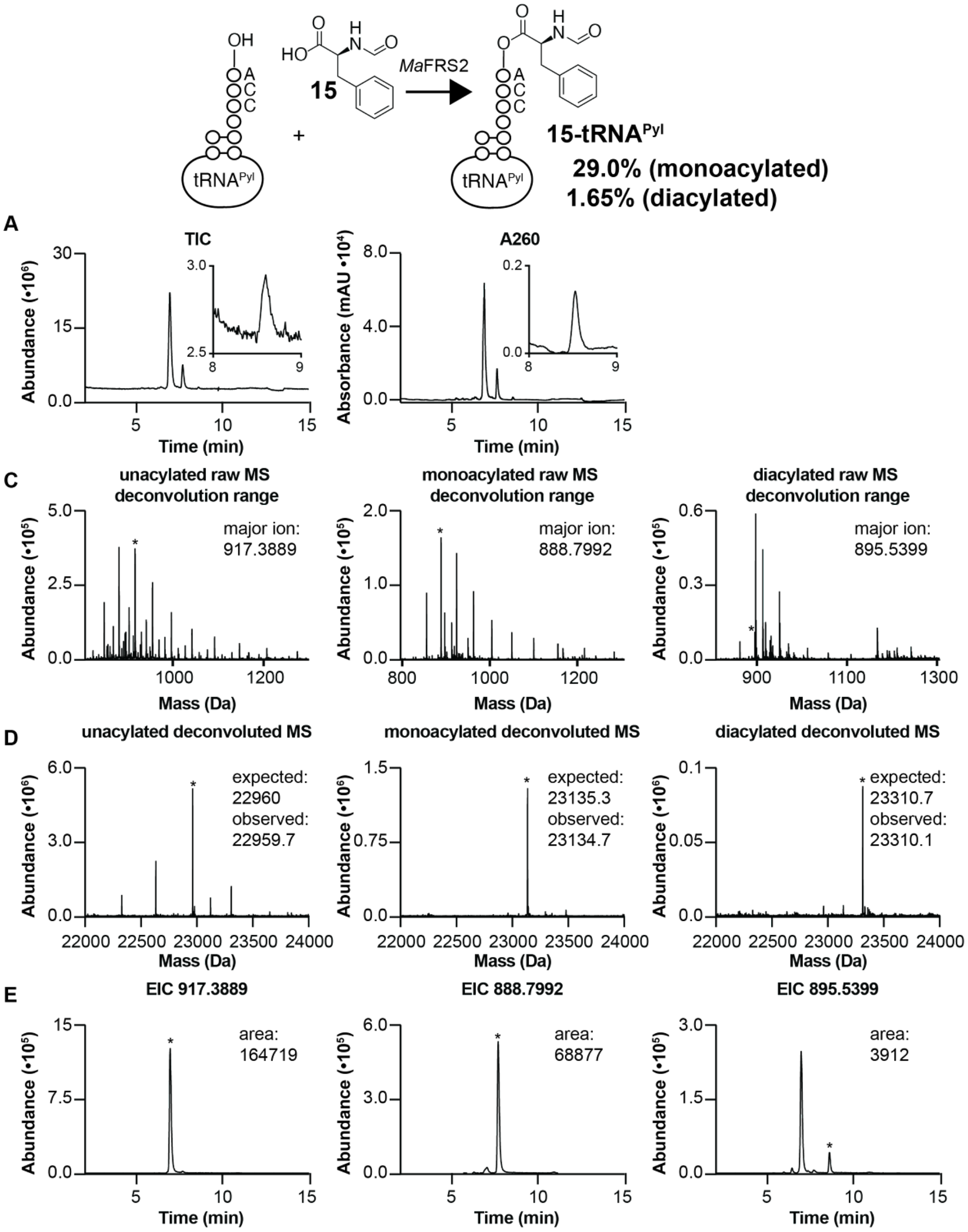

Supplementary Figure 27. Analysis of tRNA product mixtures obtained using *Ma*FRS2, *Ma*-tRNA^{Pyl} and monomer **15** as described. Please refer to the legend for Supplementary Fig. 3 for descriptions of panels **a**-**e**.

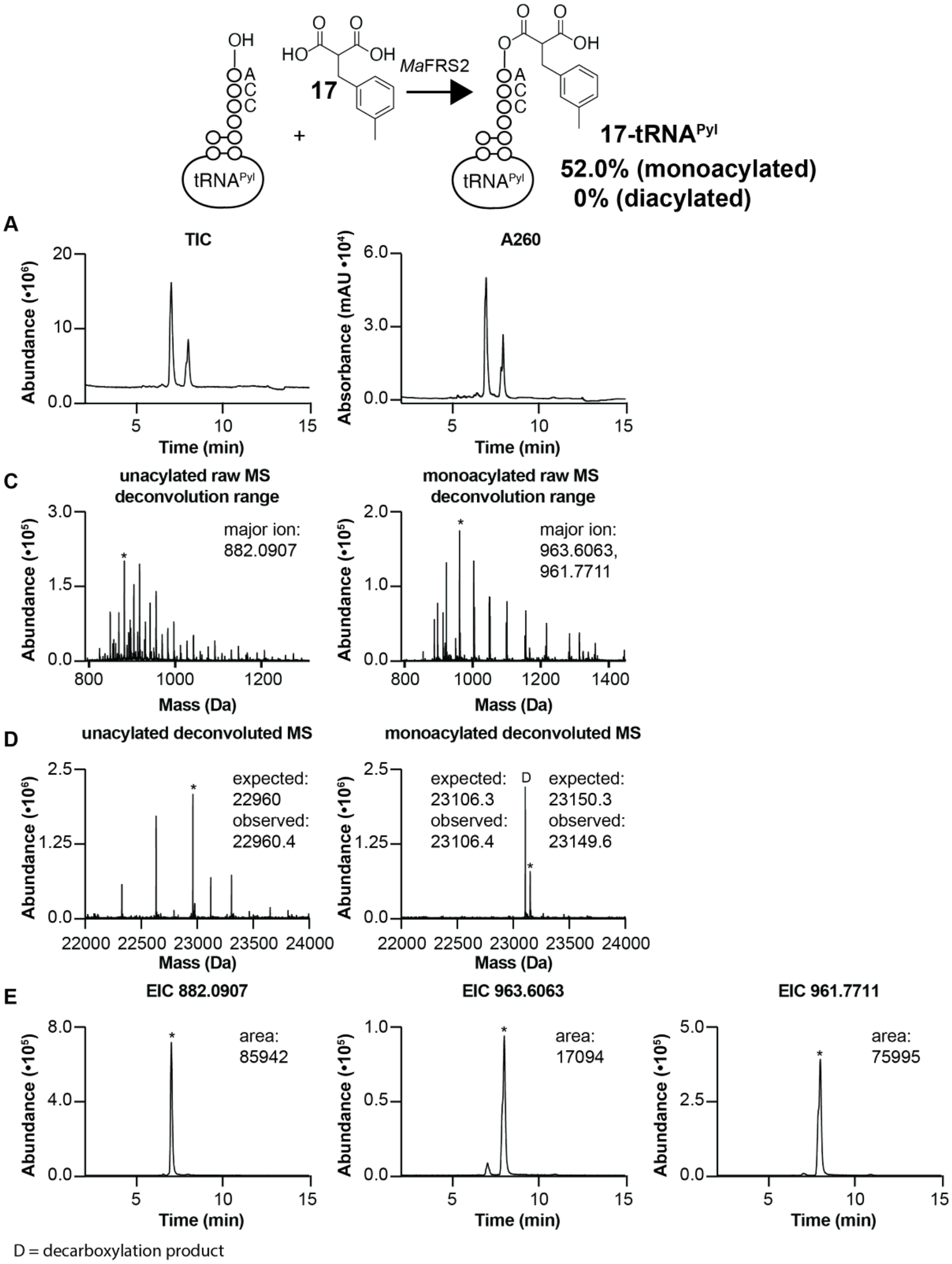

Supplementary Figure 28. Analysis of tRNA product mixtures obtained using *Ma*FRS2, *Ma*-tRNA^{Pyl} and monomer **17** as described. Please refer to the legends for Supplementary Fig. 3 for descriptions of panels **a-e** and Supplementary Fig. 7 for a note on the decarboxylation products observed in the mass spectra.

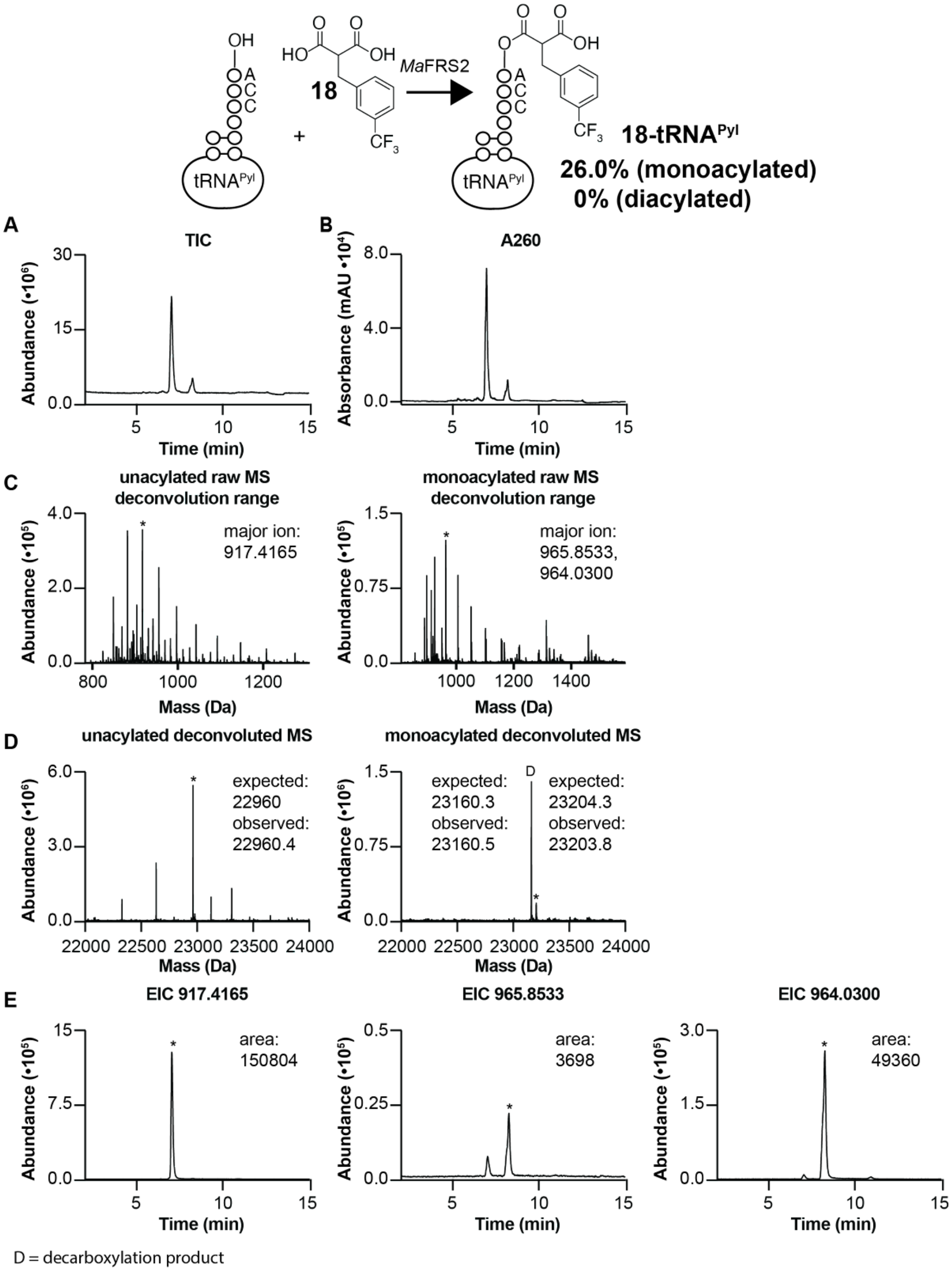

Supplementary Figure 29. Analysis of tRNA product mixtures obtained using *Ma*FRS2, *Ma*-tRNA^{Pyl} and monomer **18** as described. Please refer to the legends for Supplementary Fig. 3 for descriptions of panels **a-e** and Supplementary Fig. 7 for a note on the decarboxylation products observed in the mass spectra.

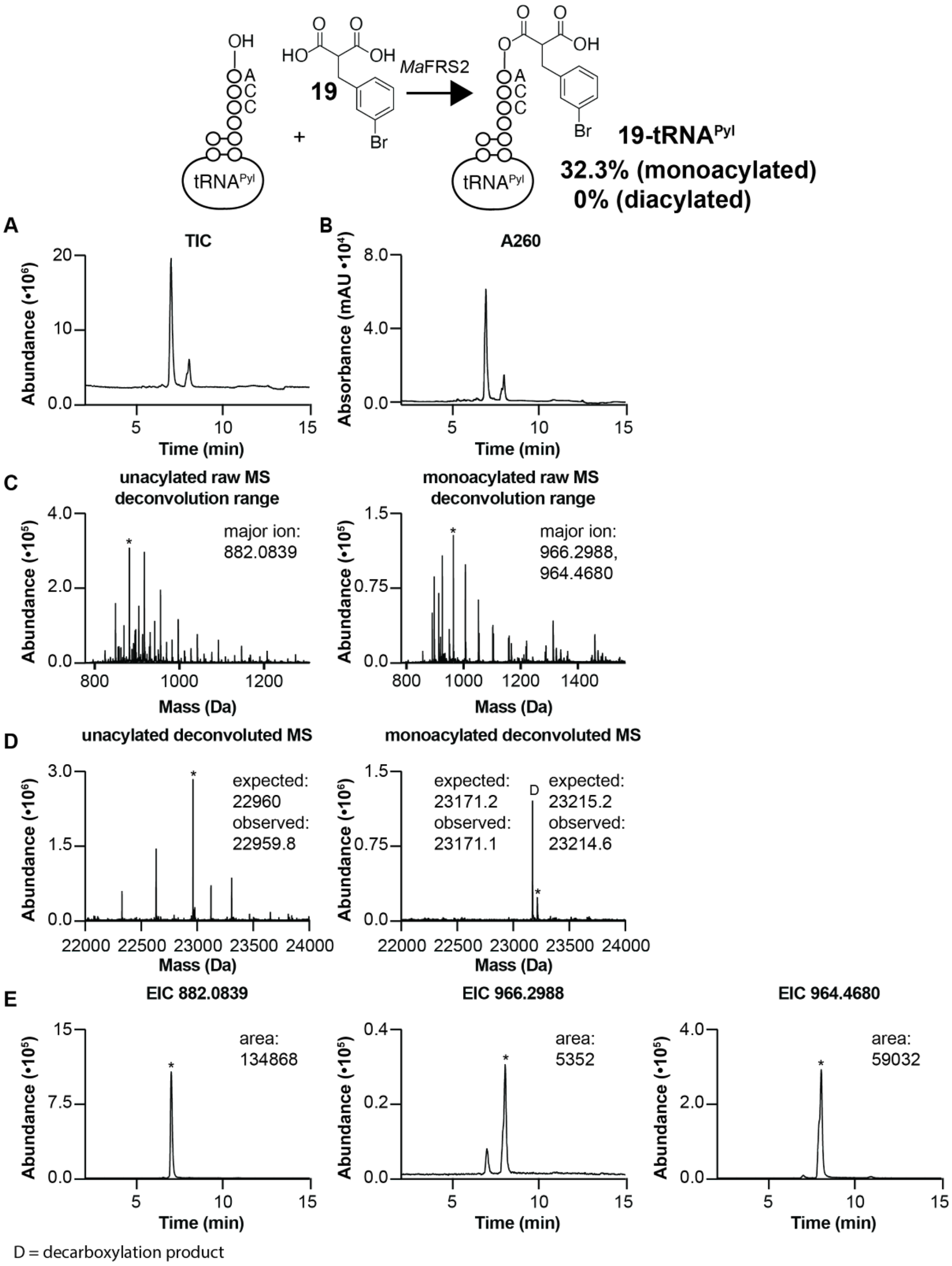

Supplementary Figure 30. Analysis of tRNA product mixtures obtained using *Ma*FRS2, *Ma*-tRNA^{Pyl} and monomer **19** as described. Please refer to the legends for Supplementary Fig. 3 for descriptions of panels **a-e** and Supplementary Fig. 7 for a note on the decarboxylation products observed in the mass spectra.

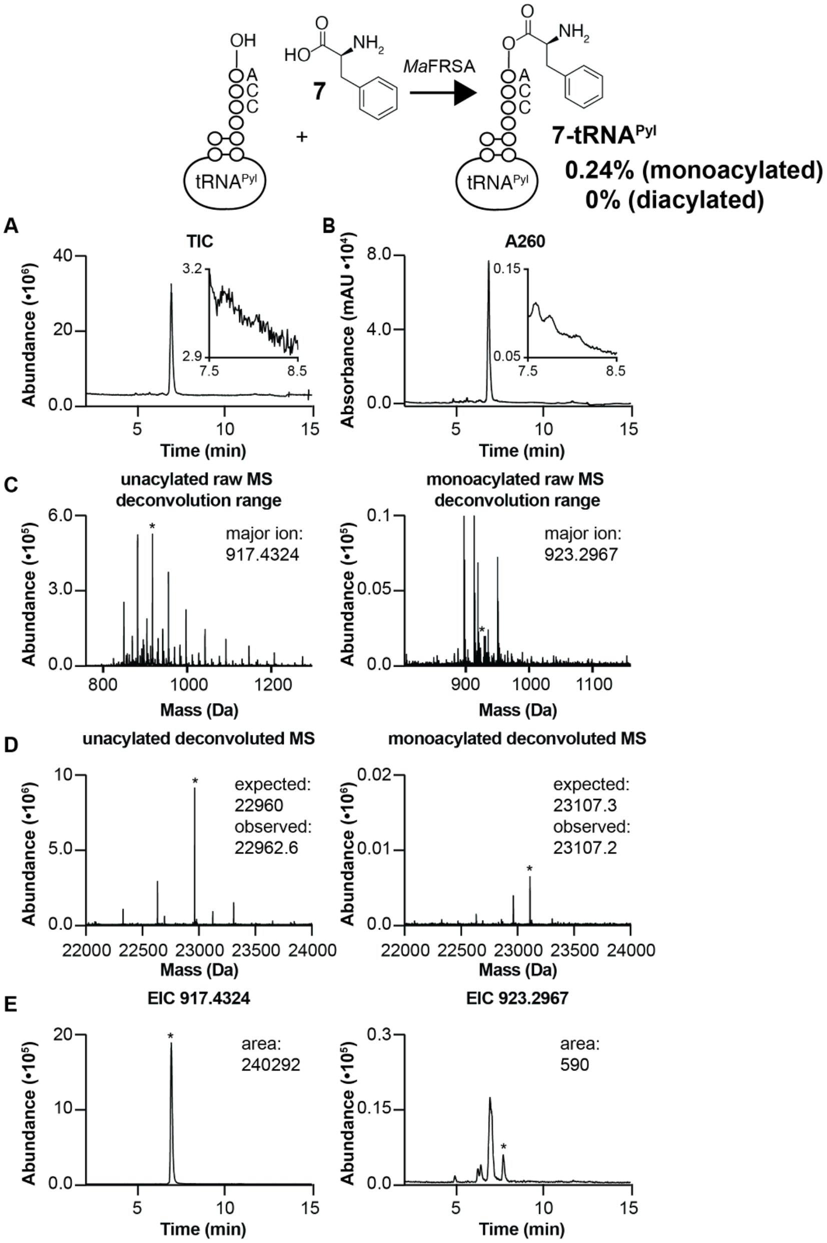

Supplementary Figure 31. Analysis of tRNA product mixtures obtained using *Ma*FRSA, *Ma*-tRNA^{Pyl} and monomer **7** as described. Please refer to the legend for Supplementary Fig. 3 for descriptions of panels **a**-**e**.

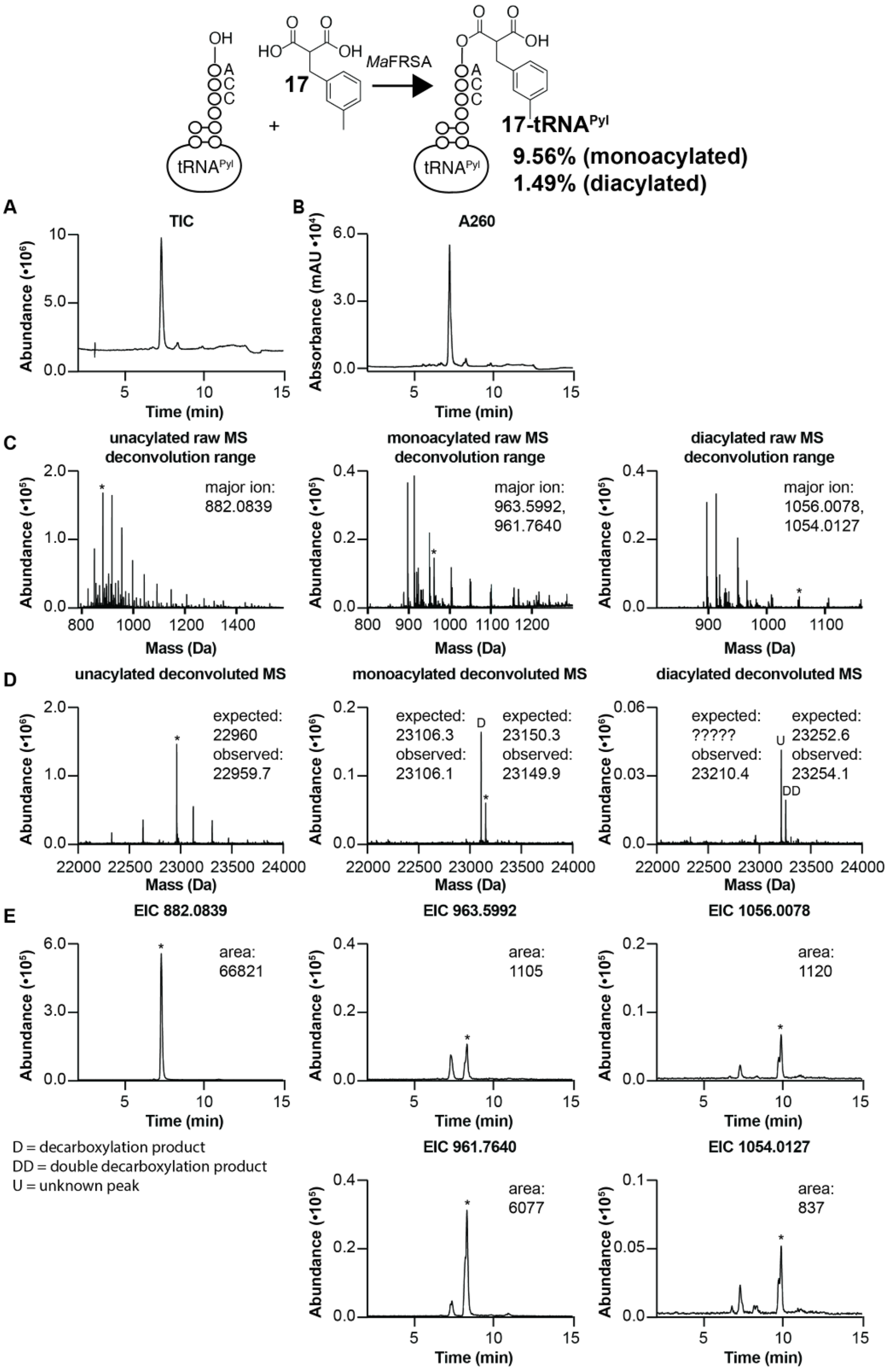

Supplementary Figure 32. Analysis of tRNA product mixtures obtained using *Ma*FRSA, *Ma*-tRNA^{Pyl} and monomer **17** as described. Please refer to the legends for Supplementary Fig. 3 for descriptions of panels **a-e** and Supplementary Fig. 7 for a note on the decarboxylation products observed in the mass spectra.

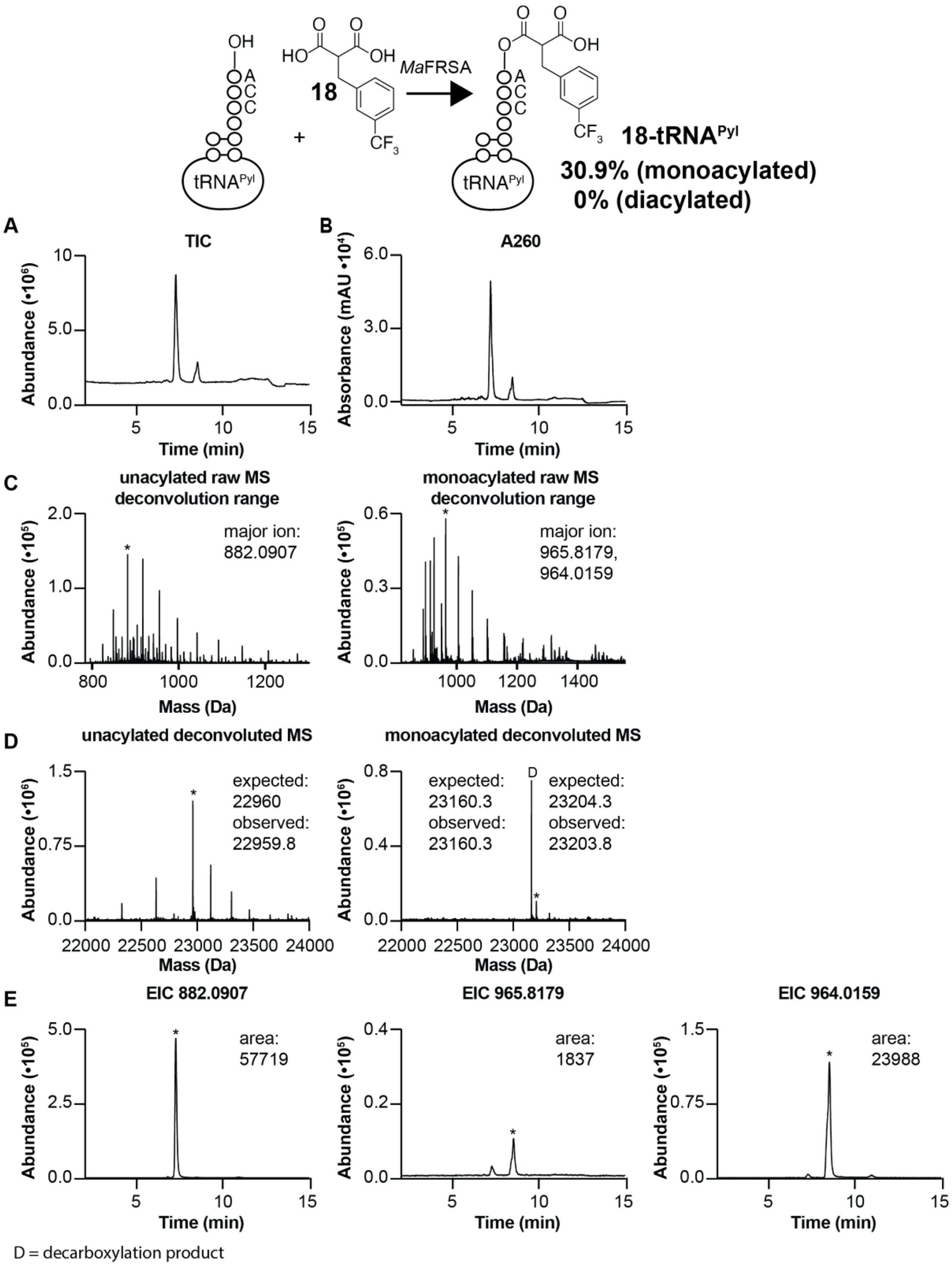

Supplementary Figure 33. Analysis of tRNA product mixtures obtained using *Ma*FRSA, *Ma*-tRNA^{Pyl} and monomer **18** as described. Please refer to the legends for Supplementary Fig. 3 for descriptions of panels **a-e** and Supplementary Fig. 7 for a note on the decarboxylation products observed in the mass spectra.

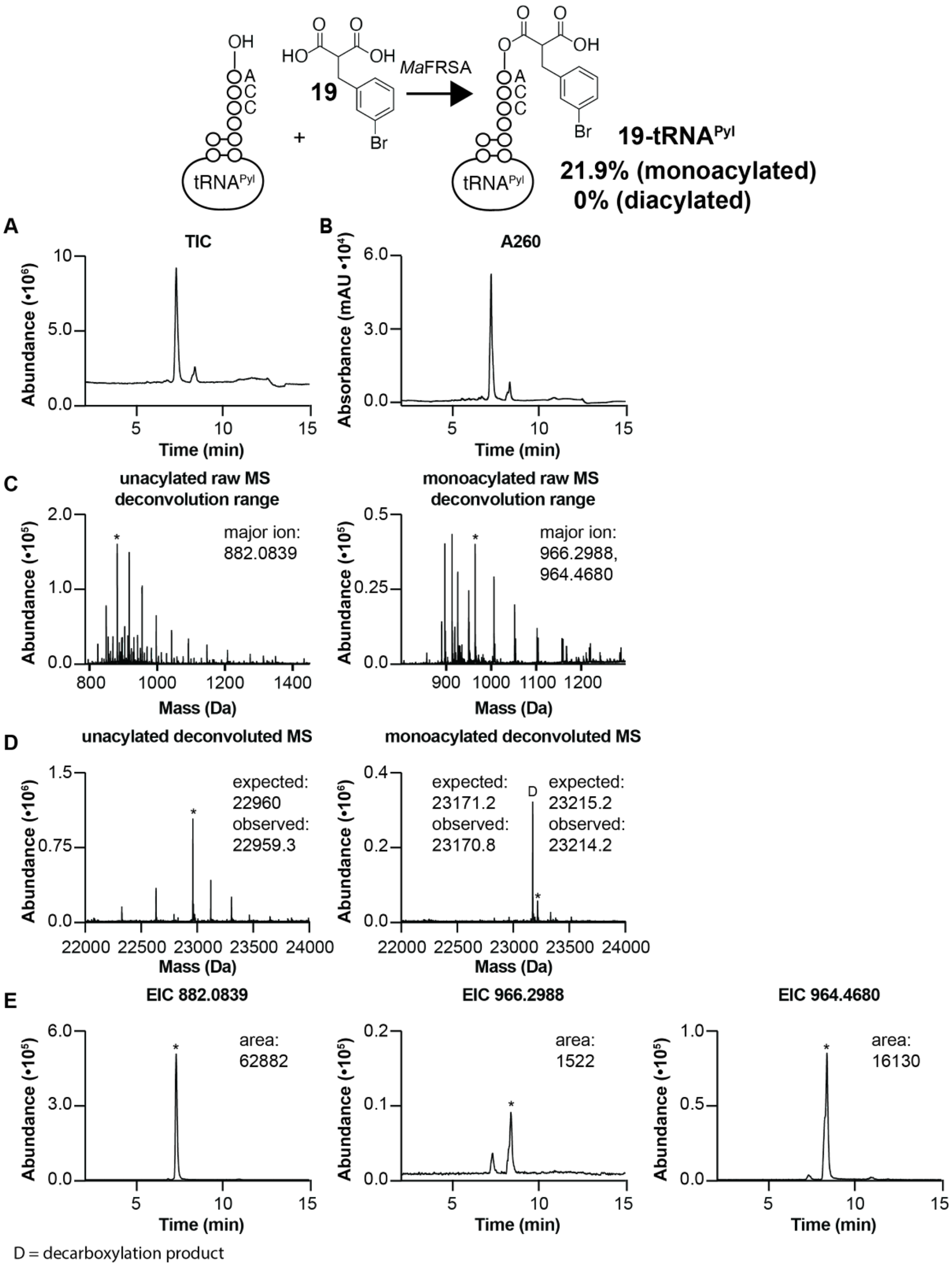

Supplementary Figure 34. Analysis of tRNA product mixtures obtained using *Ma*FRSA, *Ma*-tRNA^{Pyl} and monomer **19** as described. Please refer to the legends for Supplementary Fig. 3 for descriptions of panels **a-e** and Supplementary Fig. 7 for a note on the decarboxylation products observed in the mass spectra.

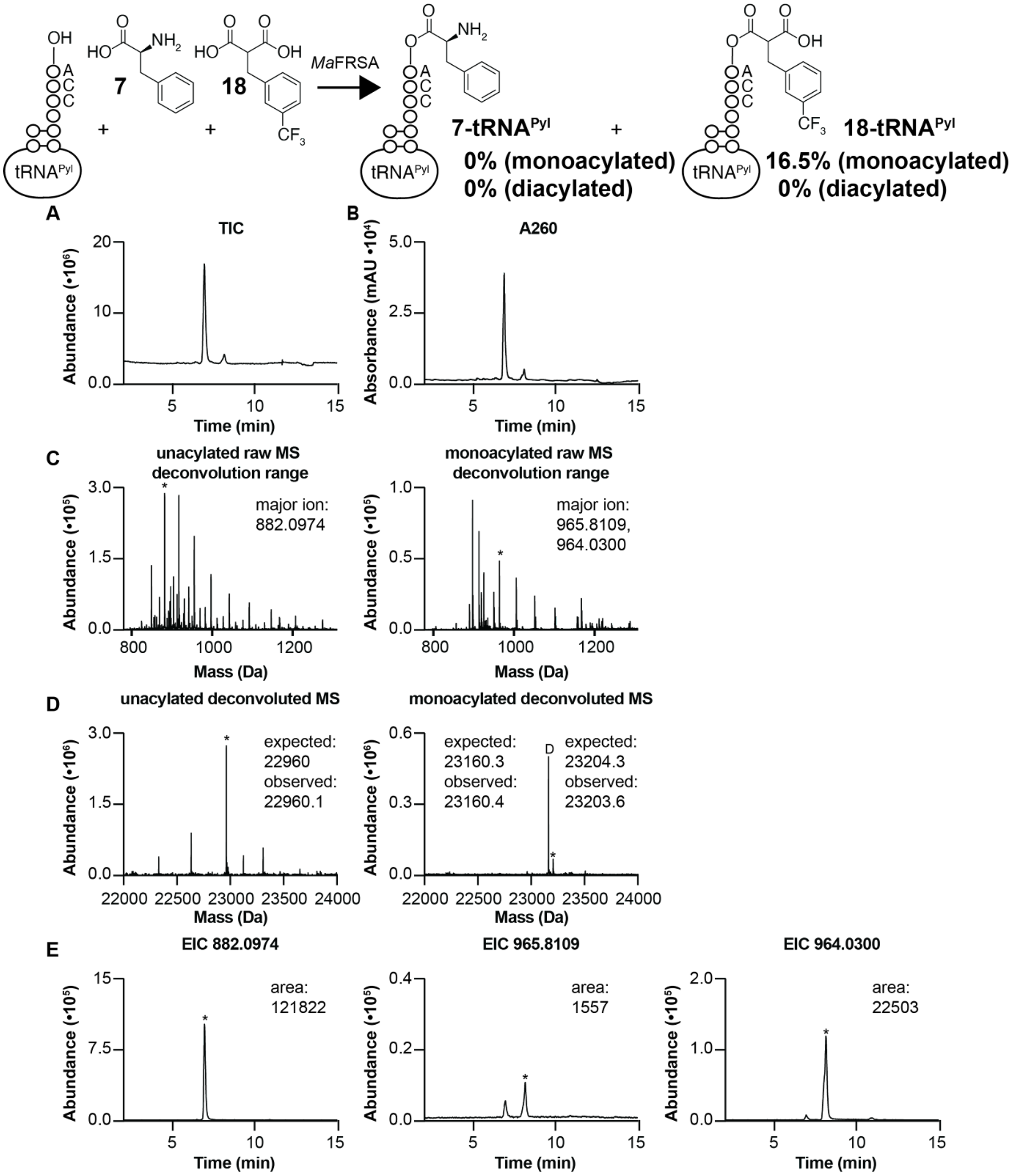

Supplementary Figure 35. Analysis of tRNA product mixtures obtained using *Ma*FRSA, *Ma*-tRNA^{Pyl} and monomers **7** and **18** as described. Please refer to the legends for Supplementary Fig. 3 for descriptions of panels **a-e** and Supplementary Fig. 7 for a note on the decarboxylation products observed in the mass spectra. Only the acylation product of **18** and *Ma*-tRNA^{Pyl} is observed.

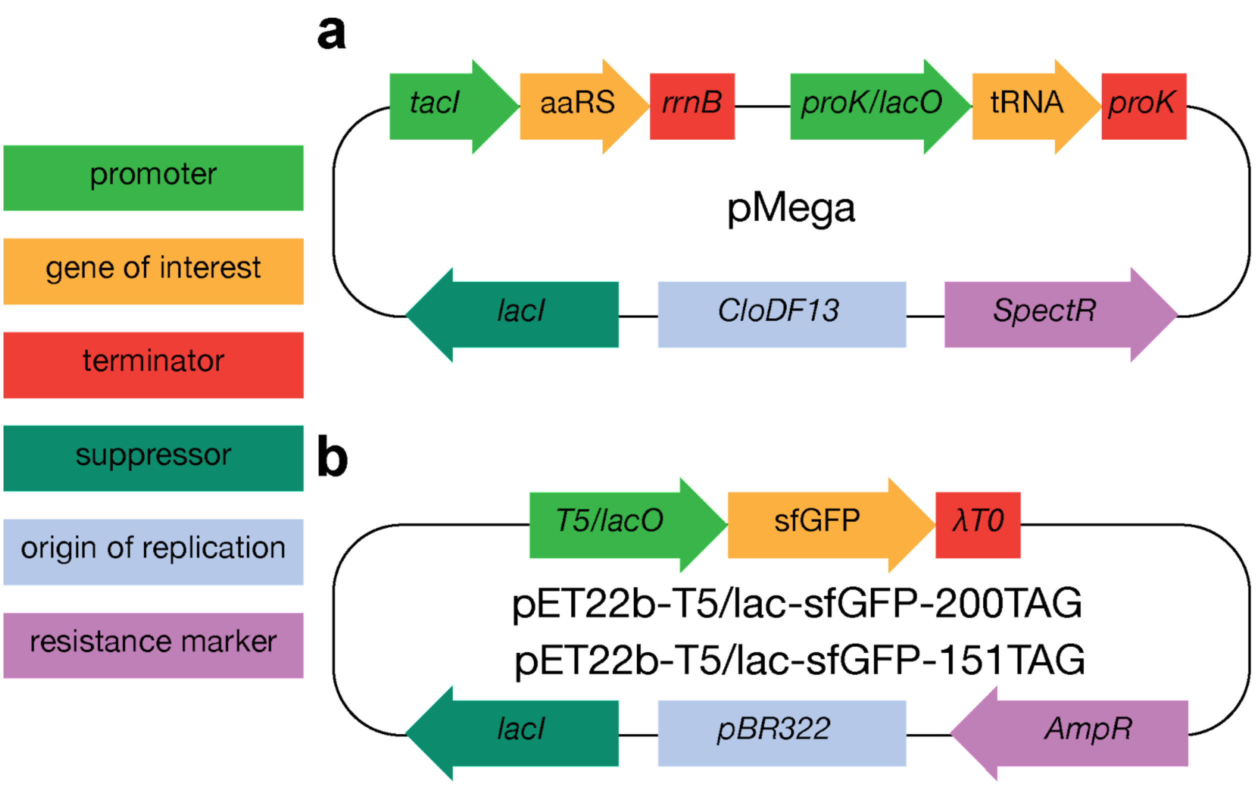

Supplementary Figure 36. Annotated maps of plasmids used for *in vivo* expression of sfGFP (Fig. 6c-e)**. a,** pMega plasmids used for *Ma*PylRS or *Ma*FRSA expression. **b,** Reporter plasmids used for expression of sfGFP with a TAG stop codon at position 200 or 151.

monomer	1	2	20	21	20	21	20	21
aaRS	Pyl	Pyl	FA	FA	FA	FA	FA	FA
cells	WT	WT	WT	WT	Δ	Δ	WT	WT
media	TB	TB	TB	TB	TB	TB	DM	DM

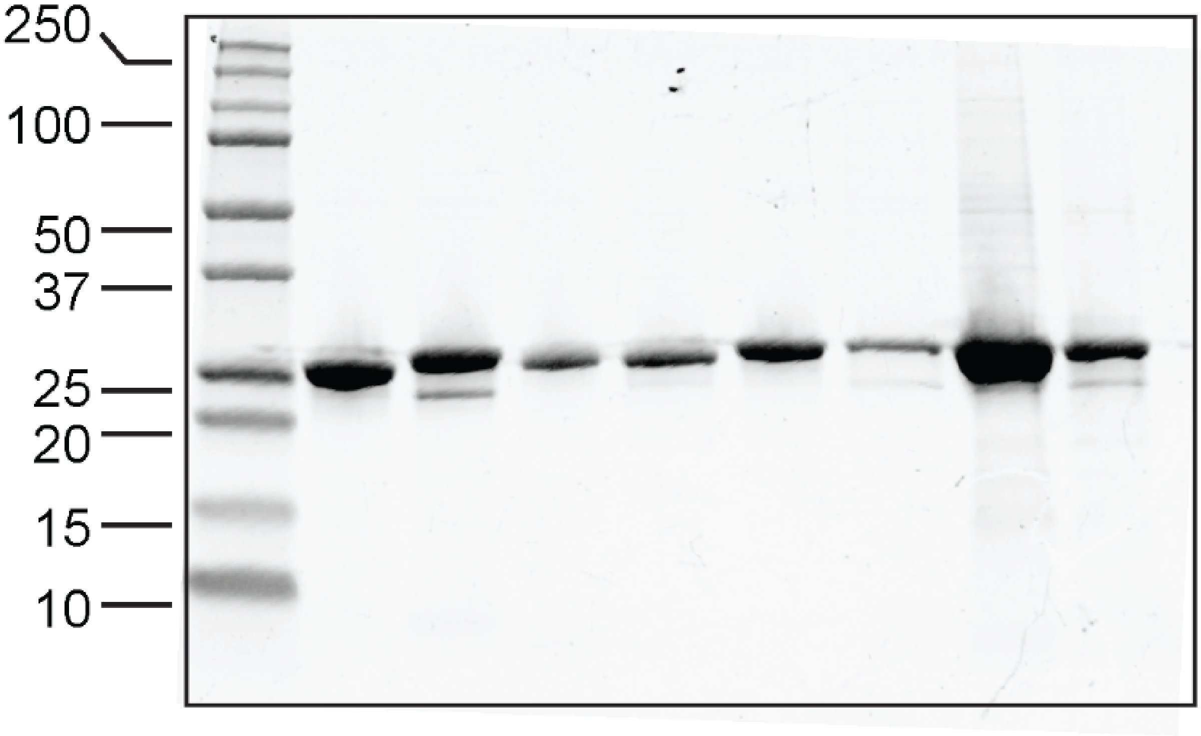

Supplementary Figure 37. Denaturing gel analysis of sfGFP variants. SDS-PAGE analysis of sfGFP variants expressed in DH10B or DH10B Δ*aspC* Δ*tyrB* in the presence of monomers **1, 2, 20,** or **21**. Abbreviations: "Pyl" = *Ma*PylRS, "FA" = *Ma*FRSA, "WT" = DH10b, "Δ" = DH10B Δ*aspC* Δ*tyrB*, "TB" = terrific broth, "DM" = defined media, +/- "OH-" = basic treatment or neutral control. Molecular weight ladder masses indicated at left in kDa.

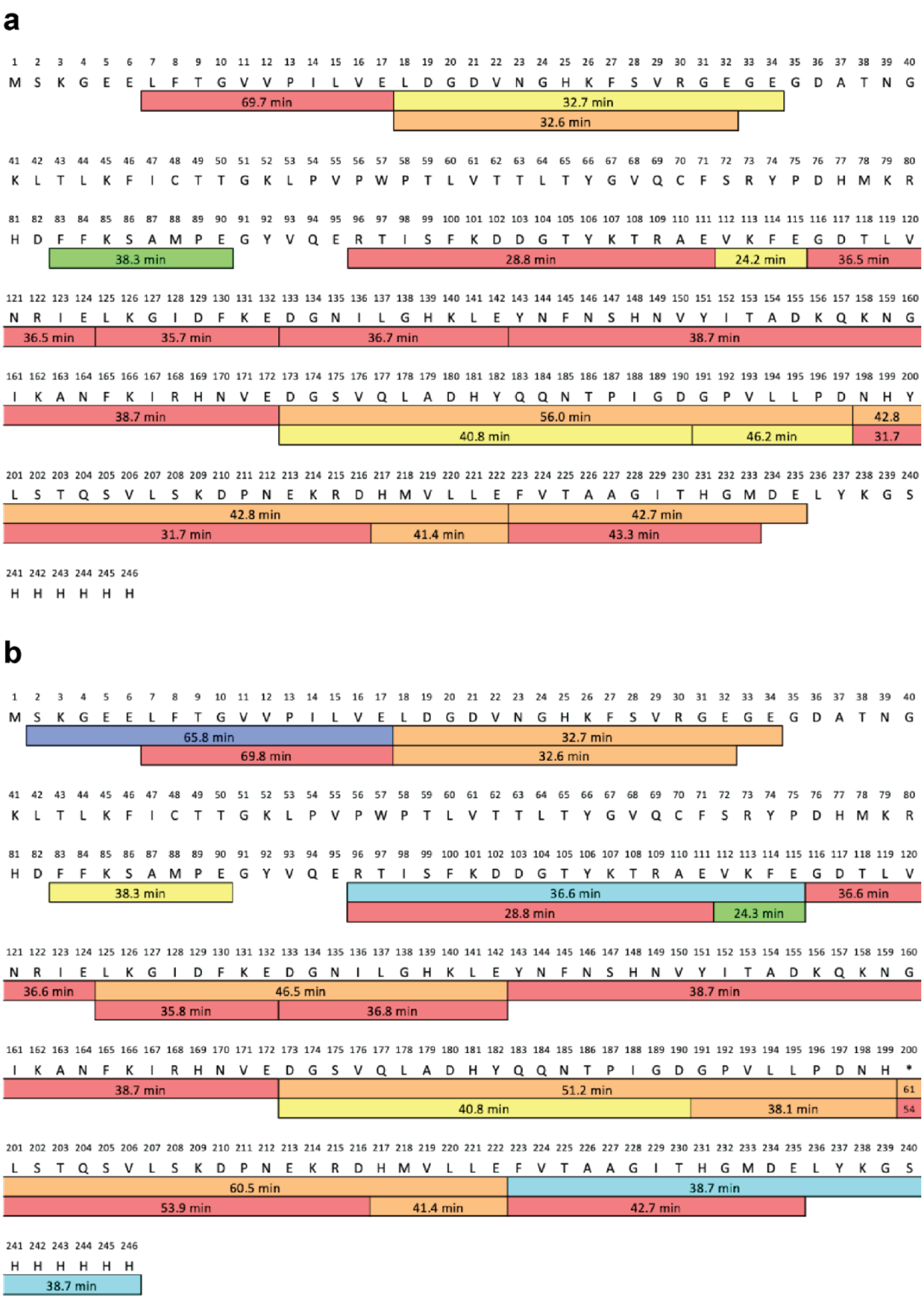
a
1 2 3 4 5 6 7 8 9 10 11 12 13 14 15 16 17 18 19 20 21 22 23 24 25 26 27 28 29 30 31 32 33 34 35 36 37 38 39 40
M S K G E E L F T G V V P I L V E L D G D V N G H K F S V R G E G E G D A T N G
69.7 min
32.7 min
32.6 min
41 42 43 44 45 46 47 48 49 50 51 52 53 54 55 56 57 58 59 60 61 62 63 64 65 66 67 68 69 70 71 72 73 74 75 76 77 78 79 80
K L T L K F I C T T G K L P V P W P T L V T T L T Y G V Q C F S R Y P D H M K R
81 82 83 84 85 86 87 88 89 90 91 92 93 94 95 96 97 98 99 100 101 102 103 104 105 106 107 108 109 110 111 112 113 114 115 116 117 118 119 120
H D F F K S A M P E G Y V Q E R T I S F K D D G T Y K T R A E V K F E G D T L V
38.3 min
28.8 min
24.2 min
36.5 min
121 122 123 124 125 126 127 128 129 130 131 132 133 134 135 136 137 138 139 140 141 142 143 144 145 146 147 148 149 150 151 152 153 154 155 156 157 158 159 160
N R I E L K G I D F K E D G N I L G H K L E Y N F N S H N V Y I T A D K Q K N G
36.5 min
35.7 min
36.7 min
38.7 min
161 162 163 164 165 166 167 168 169 170 171 172 173 174 175 176 177 178 179 180 181 182 183 184 185 186 187 188 189 190 191 192 193 194 195 196 197 198 199 200
I K A N F K I R H N V E D G S V Q L A D H Y Q Q N T P I G D G P V L L P D N H Y
38.7 min
56.0 min
42.8
40.8 min
46.2 min
31.7
201 202 203 204 205 206 207 208 209 210 211 212 213 214 215 216 217 218 219 220 221 222 223 224 225 226 227 228 229 230 231 232 233 234 235 236 237 238 239 240
L S T Q S V L S K D P N E K R D H M V L L E F V T A A G I T H G M D E L Y K G S
42.8 min
42.7 min
31.7 min
41.4 min
43.3 min
241 242 243 244 245 246
H H H H H H
b
1 2 3 4 5 6 7 8 9 10 11 12 13 14 15 16 17 18 19 20 21 22 23 24 25 26 27 28 29 30 31 32 33 34 35 36 37 38 39 40
M S K G E E L F T G V V P I L V E L D G D V N G H K F S V R G E G E G D A T N G
65.8 min
32.7 min
69.8 min
32.6 min
41 42 43 44 45 46 47 48 49 50 51 52 53 54 55 56 57 58 59 60 61 62 63 64 65 66 67 68 69 70 71 72 73 74 75 76 77 78 79 80
K L T L K F I C T T G K L P V P W P T L V T T L T Y G V Q C F S R Y P D H M K R
81 82 83 84 85 86 87 88 89 90 91 92 93 94 95 96 97 98 99 100 101 102 103 104 105 106 107 108 109 110 111 112 113 114 115 116 117 118 119 120
H D F F K S A M P E G Y V Q E R T I S F K D D G T Y K T R A E V K F E G D T L V
38.3 min
36.6 min
36.6 min
28.8 min
24.3 min
121 122 123 124 125 126 127 128 129 130 131 132 133 134 135 136 137 138 139 140 141 142 143 144 145 146 147 148 149 150 151 152 153 154 155 156 157 158 159 160
N R I E L K G I D F K E D G N I L G H K L E Y N F N S H N V Y I T A D K Q K N G
36.6 min
46.5 min
38.7 min
35.8 min
36.8 min
161 162 163 164 165 166 167 168 169 170 171 172 173 174 175 176 177 178 179 180 181 182 183 184 185 186 187 188 189 190 191 192 193 194 195 196 197 198 199 200
I K A N F K I R H N V E D G S V Q L A D H Y Q Q N T P I G D G P V L L P D N H *
38.7 min
51.2 min
61
40.8 min
38.1 min
54
201 202 203 204 205 206 207 208 209 210 211 212 213 214 215 216 217 218 219 220 221 222 223 224 225 226 227 228 229 230 231 232 233 234 235 236 237 238 239 240
L S T Q S V L S K D P N E K R D H M V L L E F V T A A G I T H G M D E L Y K G S
60.5 min
38.7 min
53.9 min
41.4 min
42.7 min
241 242 243 244 245 246
H H H H H H
38.7 min

Supplementary Figure 38. Sequence of sfGFP illustrating the peptide fragments obtained after digestion with GluC and their retention times. **a,** Fragments expected when sfGFP contains Y, BocK (**1**), or *meta*-trifluoromethyl-L-phenylalanine (**20**) at position 200. Digestion with Glu-C generates two overlapping peptides containing position 200, those encompassing residues 198-216 and 198-222. Both were used to quantify the composition at position 200. **b,** Fragments expected when sfGFP contains α-OH BocK (**2**) or α-OH *meta*-trifluoromethyl phenylalanine (**21**) at position 200. In these cases, digestion with GluC exhibited additional cleavages at the proposed ester bond (presumably due to ester hydrolysis during work-up), generating two additional peptides containing position 200, encompassing residues 200-216 and 200-222. These peptides, in addition to those encompassing residues 198-216 and 198-222, were used to quantify the composition at residue 200. Colors from red to blue represent decreasing signal intensity. Retention times are indicated in the boxes illustrating the observed peptide fragments.

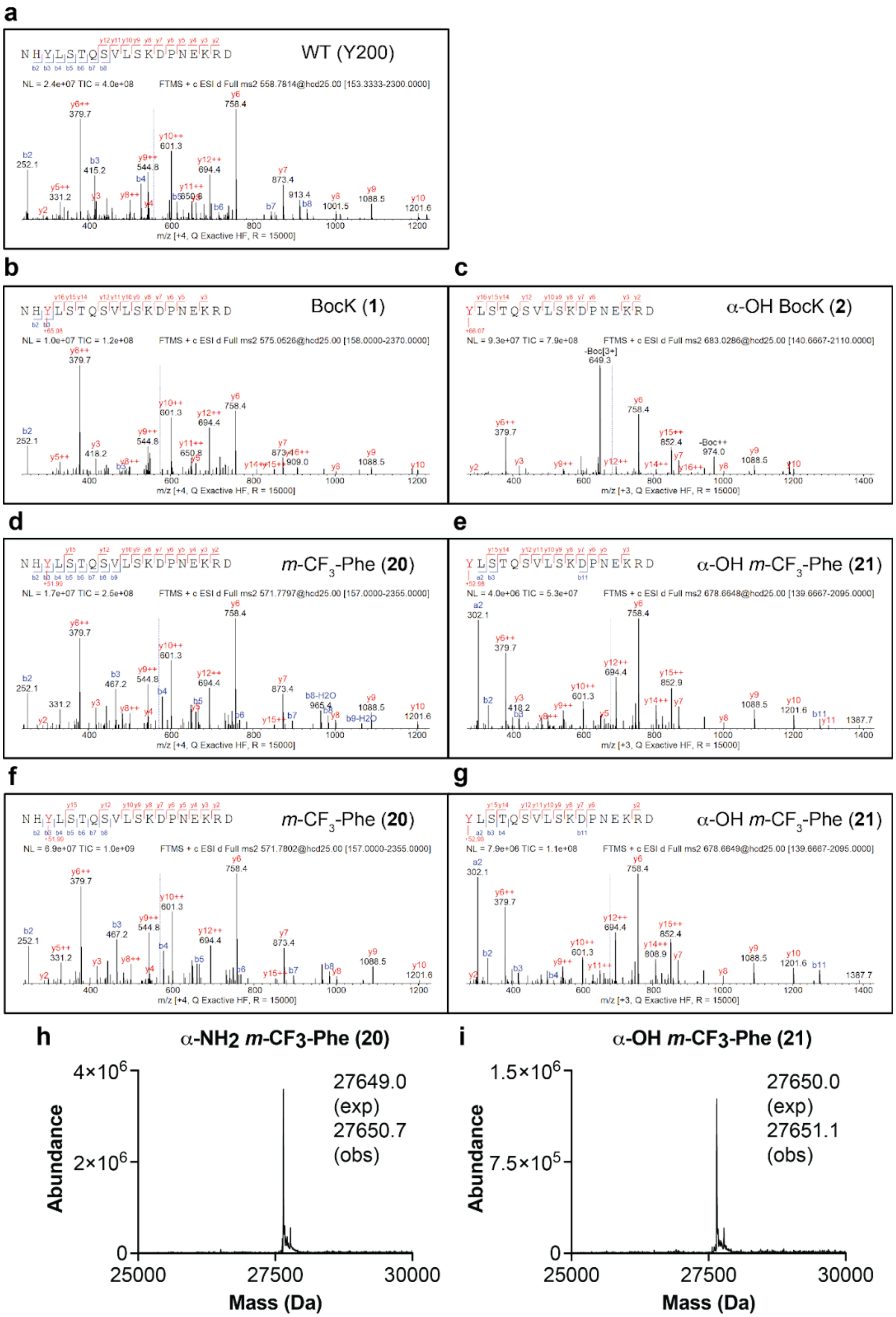

a
WT (Y200)
NHYLSTQSVLSKDPNEKRD
m/z [+4, Q Exactive HF, R = 15000]
b
BocK (1)
NHYLSTQSVLSKDPNEKRD
m/z [+4, Q Exactive HF, R = 15000]
c
α-OH BocK (2)
YLSTQSVLSKDPNEKRD
m/z [+3, Q Exactive HF, R = 15000]
d
m-CF3-Phe (20)
NHYLSTQSVLSKDPNEKRD
m/z [+4, Q Exactive HF, R = 15000]
e
α-OH m-CF3-Phe (21)
YLSTQSVLSKDPNEKRD
m/z [+3, Q Exactive HF, R = 15000]
f
m-CF3-Phe (20)
NHYLSTQSVLSKDPNEKRD
m/z [+4, Q Exactive HF, R = 15000]
g
α-OH m-CF3-Phe (21)
YLSTQSVLSKDPNEKRD
m/z [+3, Q Exactive HF, R = 15000]
h
α-NH2 m-CF3-Phe (20)
Abundance
Mass (Da)
27649.0 (exp)
27650.7 (obs)
i
α-OH m-CF3-Phe (21)
Abundance
Mass (Da)
27650.0 (exp)
27651.1 (obs)

Supplementary Figure 39. Mass spectrometry confirms the presence of an ester at position 200 of sfGFP. MS/MS identification of peptide 198-216, sequence: NHXLSTQSVLSKDPNEKRD from sfGFP expressed in DH10B cells containing **a,** tyrosine (WT); **b,** BocK (**1**); or **c,** α-OH BocK (**2**) at position 200. MS/MS identification of peptide 200-216, sequence: XLSTQSVLSKDPNEKRD resulting from sfGFP expressed in DH10B (**d, e**) or DH10B Δ*aspC* Δ*tyrB* (**f, g**) containing *meta*-trifluoromethyl-L-phenylalanine (**20**) (**d, f**) or α-OH *meta*-trifluoromethyl-L-phenylalanine (**21**) (**e, g**) at position 200. Peptides were generated by endoproteinase Glu-C digestion of sfGFP samples expressed with each indicated substrate. For fragment assignments, position 200 was considered as a tyrosine (in red) modified to have the correct mass. Intact protein mass spectra of sfGFP variants purified from DH10B Δ*aspC* Δ*tyrB* cells co-expressing *Ma*FRSA in the presence of 1 mM *meta*-trifluoromethyl phenylalanine (**20**, **h**), or α-OH *meta*-trifluoromethyl phenylalanine (**21**, **i**).

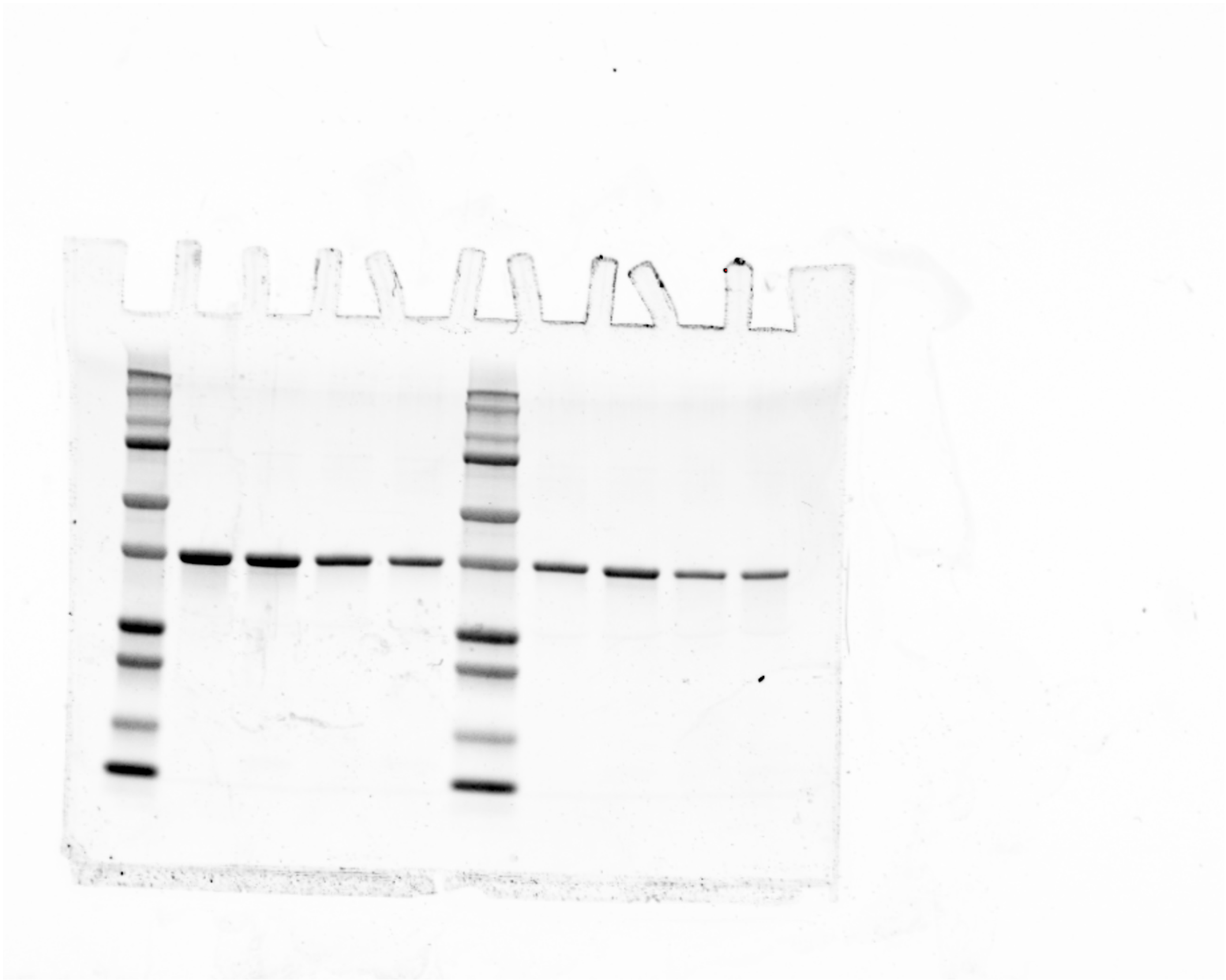

Supplementary Figure 40. Uncropped gel from Supplementary Fig. 2a.

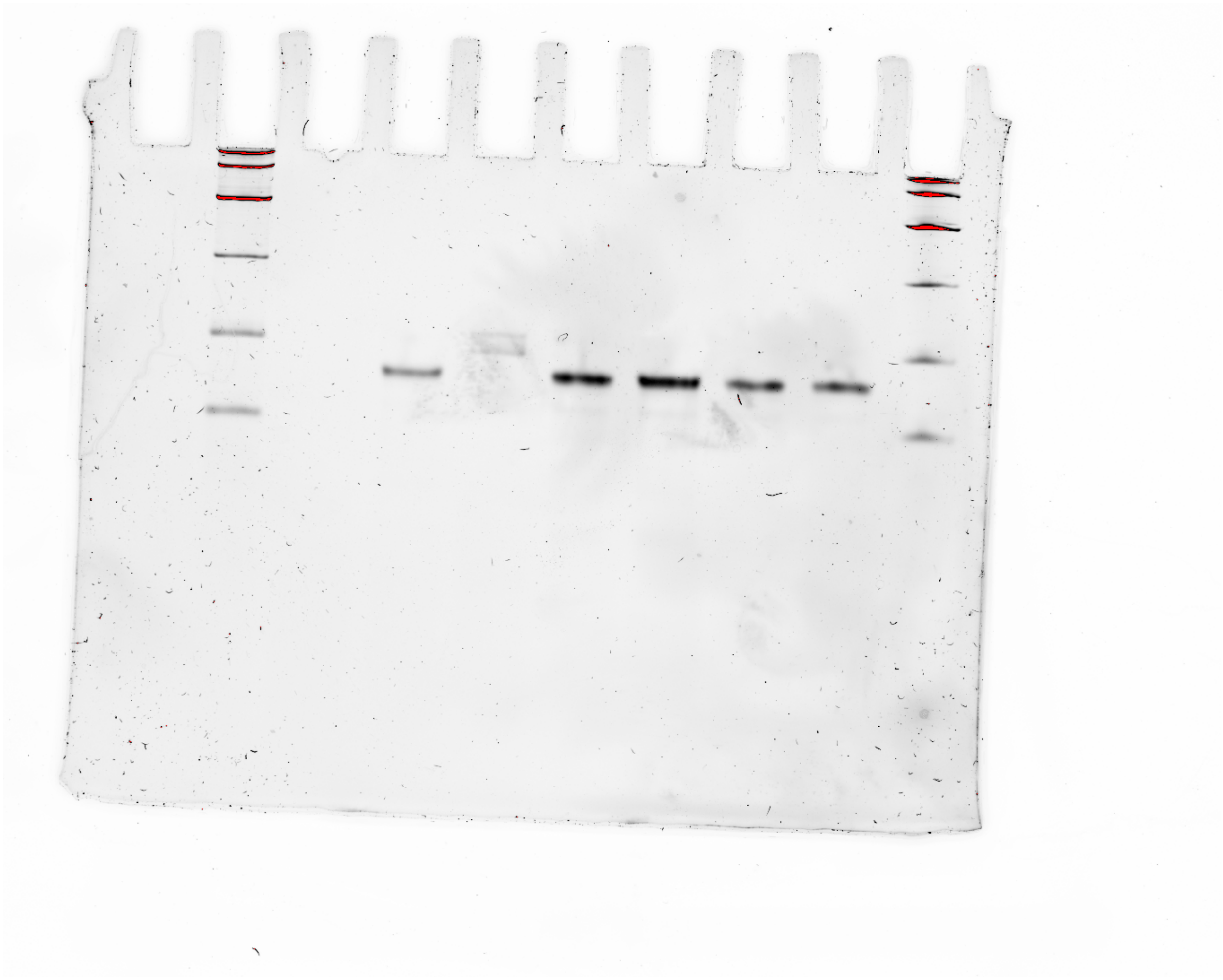

Supplementary Figure 41. Uncropped gel from Supplementary Fig. 2d.

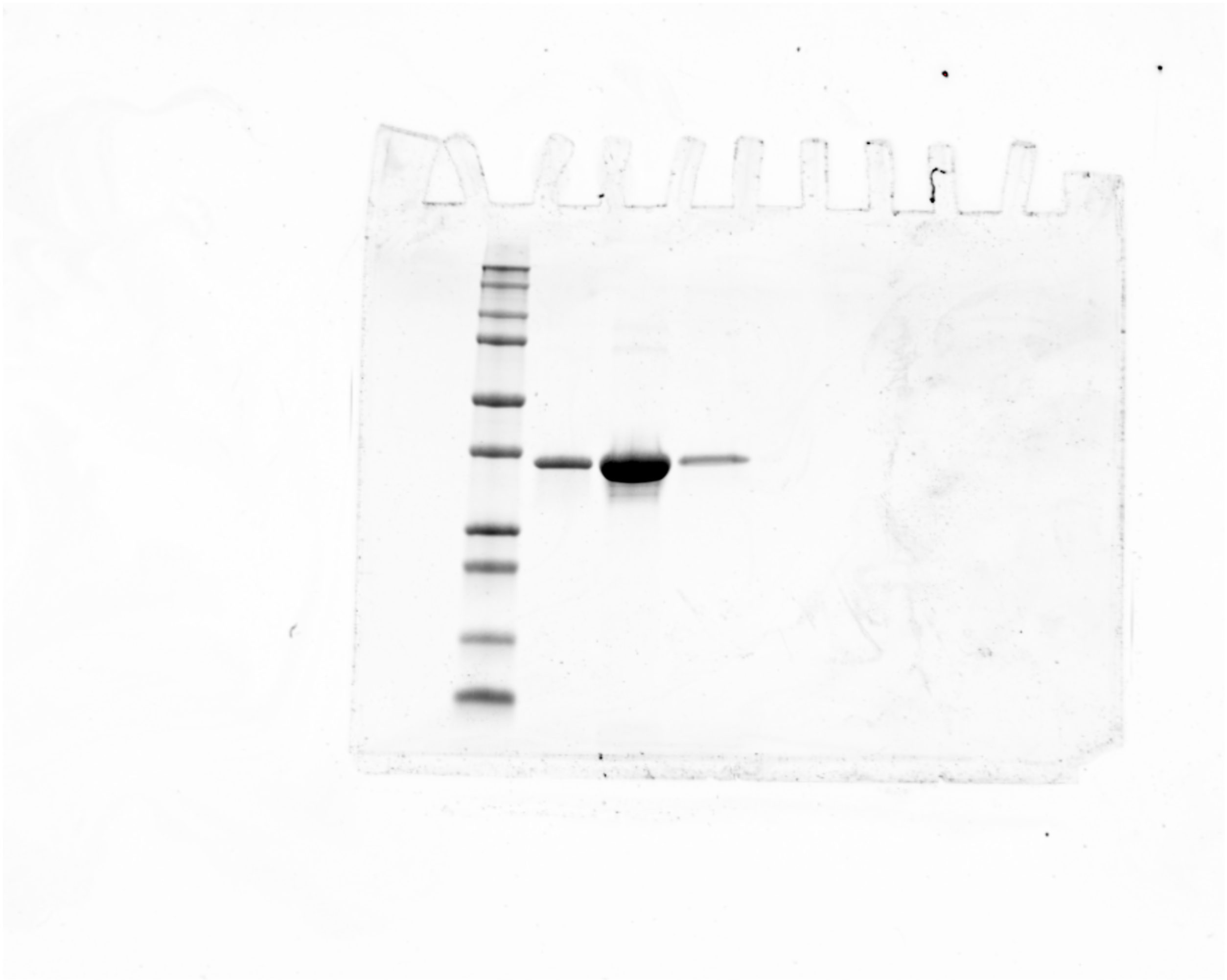

Supplementary Figure 42. Uncropped gel from Supplementary Fig. 2f.

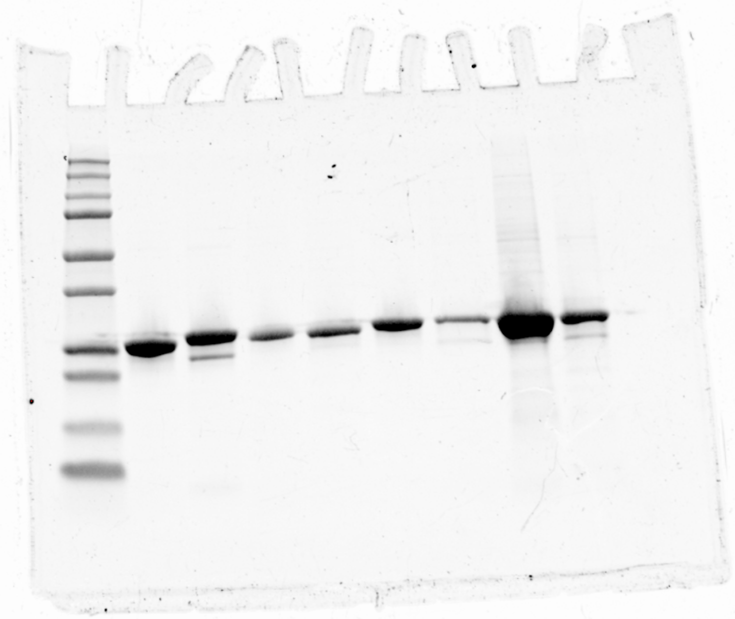

Supplementary Figure 43. Uncropped gel from Supplementary Fig. 37.

Supplementary Tables

Supplementary Table 1. Oligonucleotides used in this study

Name	Sequence (5’-3’)
pET32a<br>*Ma*PylRS<br>gBlock	ATTTTGTTTAACTTTAAGAAGGAGATATACATATGGGCAGCAGCCATCATCAT<br>CATCATCACAGCAGCGGCCTGGTGCCGCGCGGCAGCCATATGACGGTGAAA<br>TACACTGACGCACAGATCCAGCGCCTTCGCGAATATGGGAATGGCACGTATG<br>AACAGAAAGTGTTCGAAGATTTGGCTTCGCGCGACGCAGCCTTTAGCAAAG<br>AAATGAGTGTTGCCTCAACCGACAATGAGAAAAAAATTAAGGGCATGATTG<br>CCAACCCGTCACGTCATGGACTTACGCAACTTATGAACGACATTGCCGACGC<br>ATTAGTCGCTGAGGGATTTATCGAGGTCCGCACGCCAATCTTTATCTCAAAA<br>GACGCGCTTGCCCGTATGACGATTACAGAAGACAAGCCCCTGTTCAAGCAA<br>GTATTCTGGATCGACGAGAAGCGTGCCTTACGCCCAATGTTGGCTCCAAATT<br>TATATTCCGTTATGCGTGATTTGCGTGACCACACCGACGGCCCAGTGAAGAT<br>TTTCGAGATGGGGAGCTGTTTTCGCAAGGAAAGTCACAGTGGCATGCATTT<br>GGAGGAGTTCACGATGCTGAACCTTGTGGATATGGGACCGCGTGGTGATGC<br>GACAGAGGTTTTAAAAAATTACATTAGTGTTGTGATGAAAGCAGCGGGATTG<br>CCCGATTATGATTTAGTCCAGGAAGAGAGTGACGTCTACAAAGAAACTATCG<br>ATGTTGAGATTAACGGGCAAGAAGTATGTAGCGCTGCTGTCGGACCCCATTA<br>TCTGGATGCTGCCCATGATGTGCATGAACCTTGGTCTGGTGCTGGTTTCGGT<br>TTGGAGCGCTTATTAACCATTCGTGAGAAATATTCCACAGTAAAGAAAGGGG<br>GGGCAAGTATCTCGTACCTGAACGGTGCAAAAATTAATTAACATATGCACCA<br>TCACCACCATCATTCTTCTGG
pET32a	ATTTTGTTTAACTTTAAGAAGGAGATATACATATGGGCAGCAGCCATCATCAT

*Ma*FRS1<br>gBlock	CATCATCACAGCAGCGGCCTGGTGCCGCGCGGCAGCCATATGACGGTGAAA<br>TACACTGACGCACAGATCCAGCGCCTTCGCGAATATGGGAATGGCACGTATG<br>AACAGAAAGTGTTCGAAGATTTGGCTTCGCGCGACGCAGCCTTTAGCAAAG<br>AAATGAGTGTTGCCTCAACCGACAATGAGAAAAAAATTAAGGGCATGATTG<br>CCAACCCGTCACGTCATGGACTTACGCAACTTATGAACGACATTGCCGACGC<br>ATTAGTCGCTGAGGGATTTATCGAGGTCCGCACGCCAATCTTTATCTCAAAA<br>GACGCGCTTGCCCGTATGACGATTACAGAAGACAAGCCCCTGTTCAAGCAA<br>GTATTCTGGATCGACGAGAAGCGTGCCTTACGCCCAATGTTGGCTCCAAATT<br>TATATTCCGTTATGCGTGATTTGCGTGACCACACCGACGGCCCAGTGAAGAT<br>TTTCGAGATGGGGAGCTGTTTTCGCAAGGAAAGTCACAGTGGCATGCATTT<br>GGAGGAGTTCACGATGCTGGCGCTTCTGGATATGGGACCGCGTGGTGATGC<br>GACAGAGGTTTTAAAAAATTACATTAGTGTTGTGATGAAAGCAGCGGGATTG<br>CCCGATTATGATTTAGTCCAGGAAGAGAGTGACGTCTACAAAGAAACTATCG<br>ATGTTGAGATTAACGGGCAAGAAGTATGTAGCGCTGCTGTCGGACCCCATTA<br>TCTGGATGCTGCCCATGATGTGCATGAACCTTGGTCTGGTGCTGGTTTCGGT<br>TTGGAGCGCTTATTAACCATTCGTGAGAAATATTCCACAGTAAAGAAAGGGG<br>GGGCAAGTATCTCGTACCTGAACGGTGCAAAAATTAATTAACATATGCACCA<br>TCACCACCATCATTCTTCTGG
*Ma*FRSA<br>fusion tag<br>gblock	TGTGAGCGGATAACAATTCCCCTCTAGAAATAATTTTGTTTAACTTTAAGAAG<br>GAGATATACAATG<u>GGCAGCAGC</u>*CATCATCATCATCATCAC*<u>AGCAGCGGC</u>*CTGG*<br>*TGCCGCGCGGCAGC*CATATGACGGTGAAATACACTGACGCACAGATCCAGC<br>GCCTTCGCGAATATGGGAATGGCACGTA
pMega	CAATTTCACAAAGGAGGTGCGGCCGCATGACAGTCAAATATACCGACGCCC

*Ma*PylRS gBlock	AGATTCAGCGTTTGCGCGAGTACGGCAACGGTACGTATGAGCAAAAGGTTTTTGAAGACCTTGCTAGTCGCGACGCGGCGTTCAGCAAGGAAATGAGTGTTGCCTCCACAGATAATGAAAAGAAAATCAAAGGCATGATCGCCAACCCCTCTCGTCACGGCTTGACCCAGCTGATGAACGATATTGCAGATGCGTTGGTAGCTGAGGGGTTCATTGAGGTGCGTACTCCTATTTTCATTAGTAAGGACGCCCTTGCACGCATGACTATTACGGAGGATAAACCTTTGTTCAAACAGGTCTTCTGGATCGATGAGAAGCGTGCACTGCGCCCCATGCTGGCACCCAACTTATATAGTGTCATGCGCGATTTACGCGATCACACTGACGGTCCGGTGAAAATTTTTGAGATGGGCTCATGCTTCCGCAAAGAGTCGCATTCTGGTATGCACCTGGAAGAGTTTACCATGTTAAACCTGGTAGACATGGGGCCACGCGGGGACGCTACGGAGGTCCTGAAGAACTACATTAGCGTCGTGATGAAAGCAGCCGGGTTACCCGATTACGATCTTGTTCAGGAAGAGAGTGACGTTTACAAAGAAACAATCGATGTTGAAATCAACGGTCAAGAAGTTTGTTCAGCGGCCGTGGGGCCGCATTATTTAGACGCTGCCCACGATGTACATGAGCCGTGGAGTGGTGCAGGCTTCGGTCTTGAGCGCTTGCTGACCATTCGTGAGAAATATAGCACTGTCAAAAAAGGTGGCGCCTCCATCTCTTATCTTAATGGAGCTAAAATCAACTAAGCGGCCGCGTTTAAACGGTCTCCAGCTTGGCTGTTTTGGCGGATGAGAGAAGATTTTCAGCCTGATACAGATTAAATCAGAACGCAGAAGCGGTCTGATAAAACAGAATTTGCCTGGCGGCAGTAGCGCGGTGGTCCCACCTGACCCCATGCCGAACTCAGAAGTGAAACGCCGTAGCGCCGATGGTAGTGTGGGGTCTCCCCATGCGAGAGTAGGGAACTGCCAGGCATCAAATAAAACGAAAGGCTCAGTCGAAAGACTGGGCCTTGTTTGTGAGCTCCCGGTCATCAATCATCCCCATAATCCTTGTTAGCCTGCAGGTAATTCCGCTTCGCAACATGTGAGCACCGGTTTATTGACTACCGGAAGCAGTGTGACCGTGTGCTTCTCAAATGCCTGAGGCCAGTTTGCTCAGGCTCTCCCCGTGGAGGTA

	ATAATTGACGATATGATCAGTGCACGGCTAACTAAGCGGCCTGCTGACTTTC TCGCCGATCAAAAGGCATTTTGCTATTAAGGGATTGACGAGGGCGTATCTGC GCAGTAAGATAATTGTGAGCGGATAACAATTAGCAGACAAGATGGGTCCCTT ATCATGGCAACCATCTGAACGGGGGACGGTCCGGCGACCAGCGGGTCTCTA AAACCTAGCCAGCGGGGTTCGACGCCCCGGTCTCTCGCCAAATTCGAAAAG CCTGCTCAACGAGCAGGCTTTTTTGCATGCTCGAGCAGCTCAGGGTCGAA
RF31	GCTGGCGCTTAAGGATATGGGA
RF32	ATCGTGAACTCCTCCAAATG
RF33	GCTGGCGCTTGCGGATATGGGA
RF48	TGGTATGCACCTGGAAGAGTTTACCATGTTAGCGCTGGCGGACATGGGGCC ACGCGGGGA
RF49	TCCCCGCGTGGCCCCATGTCCGCCAGCGCTAACATGGTAAACTCTTCCAGG TGCATACCA
RF61	GACATGGGGCCACGC
RF62	TAACATGGTAAACTCTTCCAGGTGCA
CS43	AGACAACCATTAGCTGTCGACACAATC
CS44	GGTAAAAGGACAGGGCCA
T7 F	TAATACGACTCACTATAGGG
T7 R	GCTAGTTATTGCTCAGCGG
Ma-PylT-F	CTAATACGACTCACTATAGGGGGACGGTCCGGCGACCAGCGGGTCTCTAAA

	ACCTAGCCA
Ma-PylT-R	TmGGCGAGAGACCGGGGCGTCGAACCCCGCTGGCTAGGTTTTAGAGACCC GCTGGTCGCCG
Ma-tRNA^{Pyl}	GGGGGACGGUCCGGCGACCAGCGGGUCUCUAAAACCUAGCCAGCGGGGU UCGACGCCCCGGUCUCUCGCCA
Ma-PylT-AC C-F	CTAATACGACTCACTATAGGGGGACGGTCCGGCGACCAGCGGGTCTACCAA ACCTAGCCA
Ma-PylT-AC C-R	TmGGCGAGAGACCGGGGCGTCGAACCCCGCTGGCTAGGTTTGGTAGACCC GCTGGTCGCCG
Ma-tRNA^{Pyl}-ACC	GGGGGACGGTCCGGCGACCAGCGGGTCTACCAAACCTAGCCAGCGGGGTT CGACGCCCCGGTCTCTCGCCA
MGV-flag-1	GCGAATTAATACGACTCACTATAGGGTTAACTTTAACAAGGAGAAAAACATG GGTGTCGACTACAAGGACGACGA
MGV-flag-2	AAACCCCTCCGTTTAGAGAGGGGTTATGCTAGTTACTTGTCGTCGTCGTCCT TGTAGTCGACACCCATGTTTTTC

*mG represents 2’-O-methyl-deoxymethylguanosine

Supplementary Table 2. Expected exact masses of acyl-adenosine nucleosides extracted in LC-HRMS analysis of acyl-tRNA products digested by RNAse A.

**Substrate used**	**Exact mass**
N^{ε}-(tert-butoxycarbonyl)-L-lysine (**1**)	496.25142
(S)-6-((tert-butoxycarbonyl)amino)-2-hydroxyhexanoic acid (**2**)	497.23544
6-((tert-butoxycarbonyl)amino)hexanoic acid (**3**)	481.24052
N^{ε}-(tert-butoxycarbonyl)-D-lysine (D-BocK, **5**)	496.25142
2-(4-((tert-butoxycarbonyl)amino)butyl)malonic acid (**16**)	525.23035
L-phenylalanine (**7**)	415.17244
(S)-2-hydroxy-3-phenylpropanoic acid (**8**)	416.15646
3-phenylpropanoic acid (**9**)	400.16155
N-methyl-L-phenylalanine (**10**)	429.18809
D-phenylalanine (**11**)	415.17244
2-mercapto-3-phenylpropanoic acid (**13**)	432.13362
2-benzylmalonic acid (**14**)	444.15137
N-formyl-L-phenylalanine (**15**)	443.16736
2-(3-methylbenzyl)malonic acid (**17**)	458.16702

3-(*meta*-tolyl)propanoic acid (decarboxylation product of **17**)	414.17720
2-(3-trifluoromethylbenzyl)malonic acid (**18**)	512.13876
3-(3-(trifluoromethyl)phenyl)propanoic acid (decarboxylation product of **18**)	468.14893
2-(3-bromobenzyl)malonic acid (**19**)	522.06189
3-(3-bromophenyl)propanoic acid (decarboxylation product of **19**)	478.07206

Supplementary Table 3. Yields of mono- and diacylated *Ma*-tRNA^{Pyl} calculated from intact tRNA analysis as shown in Supplementary Figs. 3-35 and performed as described in Section IV. The number on top in each box represents the average of 3 technical replicates, while the number on the bottom is the standard deviation. ND = not determined.

**Enzyme**	***Ma*PylRS**		***Ma*FRS1**		***Ma*FRS2**		***Ma*FRSA**	
**Substrate**	mono	di	mono	di	mono	di	mono	di
**1**	53.8<br>0.3	0.0<br>0.0	ND	ND	ND	ND	ND	ND
**2**	76.8<br>0.3	13.7<br>0.2	ND	ND	ND	ND	ND	ND
**3**	59.1<br>0.5	2.51<br>0.09	ND	ND	ND	ND	ND	ND
**5**	0<br>0	0<br>0	ND	ND	ND	ND	ND	ND
**7**	ND	ND	36.2<br>1.1	29.9<br>0.7	47.7<br>0.3	17.7<br>0.3	0.24<br>0.01	0<br>0
**8**	ND	ND	26.4<br>1.0	21.0<br>0.3	36.5<br>0.5	2.78<br>0.23	ND	ND
**9**	ND	ND	0.97<br>0.03	0<br>0	1.89<br>0.02	0<br>0	ND	ND
**10**	ND	ND	1.73	0	0.55	0	ND	ND

			0.06	0	0.02	0		
**11**	ND	ND	0<br>0	0<br>0	0<br>0	0<br>0	ND	ND
**13**	ND	ND	0*<br>0	0*<br>0	2.08<br>0.06	0<br>0	ND	ND
**14**	ND	ND	24.4<br>0.8	0<br>0	43.7<br>0.5	0<br>0	ND	ND
**15**	ND	ND	9.52<br>0.28	40.5<br>0.5	29.3<br>0.3	1.80<br>0.15	ND	ND
**16**	0.25<br>0.02	0.0<br>0.0	ND	ND	ND	ND	ND	ND
**17**	ND	ND	49.8<br>0.7	3.64<br>0.35	52.0<br>0.1	0<br>0	9.48<br>0.07	1.54<br>0.10
**18**	ND	ND	59.3<br>1.0	9.24<br>0.60	26.1<br>0.4	0<br>0	31.4<br>0.7	0<br>0
**19**	ND	ND	37.4<br>0.7	10.8<br>1.4	32.7<br>0.7	0<br>0	22.3<br>0.4	0<br>0

*No product was observed when **13** was incubated with 2.5 μM *Ma*FRS1 and 25 μM *Ma*-tRNA^{Pyl}. Increasing [*Ma*FRS1] to 12.5 μM led to 0.72 ± 0.02% and 9.72 ± 0.31% of mono- and diacylated tRNA, respectively.

Supplementary Table 4. Structure refinement statistics.

	*Ma*FRSA, *meta*-CF_3-2-BMA, AMP-PNP
Wavelength (Å)	1.11583
Resolution range (Å)	45.37 - 1.803 (1.867 - 1.803)
Space group	I 4
Unit cell dimensions (Å)	108.958 108.958 112.26 90° 90° 90°
Total reflections	115764 (9210)
Unique reflections	57972 (4659)
Multiplicity	2.0 (2.0)
Completeness (%)	95.02 (66.69)
Mean I/sigma (I)	18.75 (0.43)
Wilson B-factor	39.02
R-merge	0.02241 (1.976)
R-meas	0.03169 (2.794)
R-pim	0.02241 (1.976)
CC1/2	1 (0.106)
CC*	1 (0.437)
Reflections used in refinement	57275 (4008)
Reflections used for R-free	2924 (215)
R-work	0.1800 (0.4105)

R-free	0.2115 (0.4402)
CC(work)	0.969 (0.410)
CC(free)	0.955 (0.270)
Number of non-hydrogen atoms<br><br>macromolecules<br><br>ligands<br><br>solvent	4931<br><br>4367<br><br>111<br><br>453
Protein residues	553
RMS(bonds) (Å)	0.011
RMS(angles) (°)	1.1
Ramachandran favored (%)	98
Ramachandran allowed (%)	2
Ramachandran outliers (%)	0
Rotamer outliers (%)	0.64
Clashscore	5.2
Average B-factor<br><br>macromolecules<br><br>ligands<br><br>solvent	47.8<br><br>47.42<br><br>55.19<br><br>49.59

* Parentheses indicate values for last resolution shell

Supplementary Table 5. LC-MS/MS analysis of sfGFP samples generated in DH10B and DH10B Δ*aspC* Δ*tyrB*. Sample numbers refer to column values shown in Fig. 6e. All values are in %.

**Sample**	***i***	***ii***	***iii***	***iv***	***v***	***vi***	***vii***	***viii***	***ix***
variant/ substrate	**WT**	**1**	**2**	**20**	**21**	**20**	**21**	**20**	**21**
**Y**	100.00	0.36	0.05	0.28	0.59	0.15	0.95	0.60	1.57
**1**	0.00	94.56	0.07	0.00	0.00	0.00	0.00	0.00	0.00
**2**	0.00	4.44	99.82	0.00	0.00	0.00	0.00	0.00	0.00
**20**	0.00	0.00	0.00	98.73	85.35	97.14	51.54	98.19	52.31
**21**	0.00	0.00	0.00	0.32	11.23	0.63	38.48	0.65	44.10
**A**	0.00	0.02	0.00	0.03	0.17	0.14	0.76	0.02	0.00
**F**	0.00	0.14	0.01	0.34	0.61	0.15	0.20	0.34	0.27
**K**	0.00	0.32	0.01	0.02	0.13	0.14	0.90	0.01	0.53
**Q**	0.00	0.08	0.03	0.11	0.81	0.62	3.49	0.07	0.07
**W**	0.00	0.08	0.00	0.17	1.11	1.02	3.68	0.12	0.48

References:

1. Herold, S., Bafaluy, D. & Muñiz, K. Anodic benzylic C(sp3)–H amination: unified access to pyrrolidines and piperidines. *Green Chem.* **20**, 3191–3196 (2018).

2. Matveeva, E. D. *et al.* Syntheses of Compounds Active toward Glutamate Receptors: II. Synthesis of Spiro Hydantoins of the Indan Series. *Russ. J. Org. Chem.* **38**, 1769–1774 (2002).

3. Madeira, F. *et al.* The EMBL-EBI search and sequence analysis tools APIs in 2019. *Nucleic Acids Res.* **47**, W636–W641 (2019).
