# Supplementary material for: Expanding the substrate scope of pyrrolysyl-transfer RNA synthetase enzymes to include non-α-amino acids in vitro and in vivo
Source: Nat Chem. 2023 Jun 1;15(7):960–71. doi: 10.1038/s41557-023-01224-y (PMC10322718; doi:10.1038/s41557-023-01224-y)
Supplement: Supplementary file 2 — Reporting Summary [file 41557_2023_1224_MOESM2_ESM.pdf]

nature research

Corresponding author(s): Alanna Schepartz

Last updated by author(s): Mar 24, 2022

Reporting Summary

Nature Research wishes to improve the reproducibility of the work that we publish. This form provides structure for consistency and transparency in reporting. For further information on Nature Research policies, see our Editorial Policies and the Editorial Policy Checklist.

Statistics

For all statistical analyses, confirm that the following items are present in the figure legend, table legend, main text, or Methods section.

n/a	Confirmed	
☐	☒	The exact sample size (n) for each experimental group/condition, given as a discrete number and unit of measurement
☐	☒	A statement on whether measurements were taken from distinct samples or whether the same sample was measured repeatedly
☒	☐	The statistical test(s) used AND whether they are one- or two-sided<br>*Only common tests should be described solely by name; describe more complex techniques in the Methods section.*
☒	☐	A description of all covariates tested
☒	☐	A description of any assumptions or corrections, such as tests of normality and adjustment for multiple comparisons
☐	☒	A full description of the statistical parameters including central tendency (e.g. means) or other basic estimates (e.g. regression coefficient) AND variation (e.g. standard deviation) or associated estimates of uncertainty (e.g. confidence intervals)
☒	☐	For null hypothesis testing, the test statistic (e.g. F, t, r) with confidence intervals, effect sizes, degrees of freedom and P value noted<br>*Give P values as exact values whenever suitable.*
☒	☐	For Bayesian analysis, information on the choice of priors and Markov chain Monte Carlo settings
☒	☐	For hierarchical and complex designs, identification of the appropriate level for tests and full reporting of outcomes
☒	☐	Estimates of effect sizes (e.g. Cohen's d, Pearson's r), indicating how they were calculated

Our web collection on statistics for biologists contains articles on many of the points above.

Software and code

Policy information about availability of computer code

Data collection	RNA MS spectra were collected with MassLynx V4.2. Peptide, protein, and acyl-adenosine MS spectra were collected with MassHunter B10. Adenylation assays were collected with BioTek Gen5 3.08. Diffraction data were collected with custom software of the ALS Beamline 8.3.1.
Data analysis	Crystallographic data were analyzed using XDS version Feb 5, 2021, CCP4I version 7.1.017, Phenix version 1.19.2, Coot version 0.8.9.2, and Pymol version 2.4.2. RNA MS spectra were deconvoluted with MaxEnt1. Proteolytic peptides were identified and quantified on MassAnalyzer available in Biopharma FinderTM from Thermo Fisher. LC-MS traces and adenylation reactions were plotted in Prism 9. Figures were generated in Adobe Illustrator version 26.0.2. Protein sequence alignment was performed with EMBOSS Needle.

For manuscripts utilizing custom algorithms or software that are central to the research but not yet described in published literature, software must be made available to editors and reviewers. We strongly encourage code deposition in a community repository (e.g. GitHub). See the Nature Research guidelines for submitting code & software for further information.

Data

Policy information about availability of data

All manuscripts must include a data availability statement. This statement should provide the following information, where applicable:

- Accession codes, unique identifiers, or web links for publicly available datasets
- A list of figures that have associated raw data
- A description of any restrictions on data availability

The structural model of MaFRSA is available in the Protein Data Bank with code 7U0R. The source data that support the findings of this study are provided with this paper (Figs. 1-6 and Extended Data Figs. 1-6) or are available at Zenodo DOI:10.5281/zenodo.7754396 (Supplementary Figures).

Field-specific reporting

Please select the one below that is the best fit for your research. If you are not sure, read the appropriate sections before making your selection.

☒ Life sciences ☐ Behavioural & social sciences ☐ Ecological, evolutionary & environmental sciences

For a reference copy of the document with all sections, see nature.com/documents/nr-reporting-summary-flat.pdf

Life sciences study design

All studies must disclose on these points even when the disclosure is negative.

Sample size	A sample size three was used for malachite green assays and tRNA LC-MS assays, and a sample size of four was used for fluorescence plate reader assays. A sample of 57,275 reflections were used for structure solution.
Data exclusions	No data was excluded in this study.
Replication	All acylation reactions were analyzed with two assays: RNAse A assay and intact tRNA LC-MS. Three replicate injections were performed for intact tRNA LC-MS assays and yields were calculated from averages. Adenylation assays were performed in biological triplicate. In vitro translation reactions were performed twice. All attempts at replication were successful.
Randomization	A subset of 5% of reflections (2924) were randomly selected by Phenix for calculating Rfree and were not used in refinement. Randomization was not performed for biochemical assays. Randomization is not relevant because the samples form defined groups.
Blinding	Blinding was not performed for biochemical assays. Blinding is not relevant to this study. The groups were defined and studied by the investigators using standard protocols.

Reporting for specific materials, systems and methods

We require information from authors about some types of materials, experimental systems and methods used in many studies. Here, indicate whether each material, system or method listed is relevant to your study. If you are not sure if a list item applies to your research, read the appropriate section before selecting a response.

Materials & experimental systems

n/a	Involved in the study	
☒	☐	Antibodies
☒	☐	Eukaryotic cell lines
☒	☐	Palaeontology and archaeology
☒	☐	Animals and other organisms
☒	☐	Human research participants
☒	☐	Clinical data
☒	☐	Dual use research of concern

Methods

n/a	Involved in the study	
☒	☐	ChIP-seq
☒	☐	Flow cytometry
☒	☐	MRI-based neuroimaging
